# Milk Exosomes Transfer Oligosaccharides into Macrophages to Modulate Immunity and Attenuate Adherent-Invasive *E. coli* (AIEC) Infection

**DOI:** 10.3390/nu13093198

**Published:** 2021-09-14

**Authors:** Yingying He, Zhicheng He, Serena Leone, Shubai Liu

**Affiliations:** 1 State Key Laboratory of Phytochemistry and Plant Resources in West China, Kunming Institute of Botany, Chinese Academy of Sciences, Kunming 650201, China; yingying.he10@gmail.com (Y.H.); hezhicheng@mail.kib.ac.cn (Z.H.); 2 School of Chemical Science & Technology, Yunnan University, Kunming 650091, China; 3 University of Chinese Academy of Sciences, Beijing 100049, China; 4 Department of Chemical Sciences, University of Naples ‘Federico II’, 80121 Napoli, Italy; serena.leone@unina.it

**Keywords:** HMOs, exosome, mucosal immunity, 2′-FL, macrophages

## Abstract

Exosomes are abundance in human body fluids like urine, milk and blood. They act a critical role in extracellular and intracellular communication, intracellular trafficking and physiological regulation. Multiple immune-modulatory components, such as proteins, RNAs and carbohydrates (glycoproteins), have been found in human milk exosomes, which play immune-regulatory functions. However, little is known about oligosaccharides in milk exosomes, the “free sugars”, which act critical roles in the development of infant’s immature mucosal immune system. In this study, the profile of milk exosomes encapsulated human milk oligosaccharides (HMOs) was calibrated with characteristic oligosaccharides in colostrum and mature milk, respectively. The exosomes containing human milk oligosaccharides were uptaken by macrophages, which were responsible for the establishment of intestinal immunity. Furthermore, mice pretreated with exosome encapsulated HMOs were protected from AIEC infection and had significantly less LPS-induced inflammation and intestinal damage. Exosome encapsulated milk oligosaccharides are regarded to provide a natural manner for milk oligosaccharides to accomplish their critical functions in modifying newborn innate immunity. The understanding of the interaction between a mother’s breastfeeding and the development of an infant’s mucosal immune system would be advantageous. The transport of milk oligosaccharides to its target via exosome-like particles appears to be promising.

## 1. Introduction

Mother’s milk, as the natural food for infants, contains quantitative and proportional nutrients that promote brain, intestinal, and immune develop rapidly in neonates [1]. Human milk, which contains thousands of indigestible bioactive molecules (protein, microRNA, oligosaccharides), has recently been shown to modulate neonate’s intestinal mucosal immunity, protect infants from infection and inflammation damage, and contribute to healthy microbial colonization of the neonatal intestine and promote organs development [2,3]. There are three major proposed manners and mechanisms for breastfeeding mother’s milk to protect their children that have been postulated, including inhibiting pathogen binding, acting as natural prebiotics, and modulating the development and differentiation of mucosal immune system [1,2,4].

Exosomes (40~150 nm), microvesicles and multivesicular endosomes (MVE) (100~1000 nm) are different forms of extracellular vehicles (EVs), which are secreted small envelope particle from cells endosomal lumen [5,6,7]. EVs are abundant in human body fluids, such as blood, urine, milk and saliva. The secretion of exosomes is tightly controlled, and they play a critical role in cell-to-cell communication, intracellular trafficking and physiological regulation [8,9]. The exosomes capsule contains a plenty of bioactive contents (immune regulatory proteins, mRNA and miRNA), and the native method of cargos uptake through target cells endocytosis has been uncovered [8,9,10]. Components that execute biological functions and modulate the development of baby immune system have been discovered in milk exosomes [11,12]. The distribution of milk oligosaccharides that are majority active component in the milk exosomes is poorly understood.

Human milk oligosaccharides (HMOs) are the third most abundant solid component of milk [1,13]. More than 200 individual oligosaccharides have been found and characterized in human milk that have been divided into neutral and acidic oligosaccharides. Colostrum contains more active oligosaccharides than mature milk [1,13]. HMOs have been demonstrated extensive anti-inflammatory activities through modulating cytokine production, protecting against the development of inflammatory bowel diseases [14,15], and regulating the development of intestinal mucosal immunity [2,3]. HMOs that acted in a number of ways by reducing the leukocyte adhesion and specifically binding to dendritic cells via lectin receptor DC-SIGN [16], 2-fucosyllactose (2′-FL) and Sialyl (α2,3) lactose (3-SL) regulating inflammation through CD14/TLR4 [3,17].

The mucosa and deeper layers of the baby intestine contain many immune regulatory cells, such as macrophages and dendritic cells [2,18]. These cells recognize the pathogen associated molecular patterns (PAMPs) that associated with microbial pathogens via pattern-recognition receptors (PRRs) modulate the inflammation response, and transform intestinal mucosal immunity system from innate immunity to adoptive immunity, maintaining immune tolerance and balance [19,20]. Intestinal macrophages are among the most common leukocytes in the body and they play a key role in mucosa homeostasis by facilitating clear apoptotic cells, wound healing and immune regulation [18,19,21]. Aside than the classical inflammation response, intestinal macrophages can be triggered to become anti-inflammatory, phagocytosis neutrophils and apoptotic cell debris [22,23]. Alternative phenotype macrophages express or secrete anti-bacterial and proteolytic enzymes, chemokines and anti-inflammatory cytokines, such as IL-10, IL-4 and IL-6, IL-1R antagonist, and production of growth factors, such as vascular endothelial growth factor (VEGF) and insulin-like growth factor (IGF)-1 by via PRR activation [22,24,25]. This flip represents a shift from inflammation to proliferation throughout the healing process [25].

In this study, the microparticle dynamic tracking technologies were employed to explore the properties of human milk exosomes and the profile of milk exosomes encapsulated oligosaccharides. The oligosaccharide profiles of colostrum and mature milk exosomes encapsulated had been characterized by LC-MS. THP-1 was used as the cell model to explore the immunological regulatory function of milk exosomes encapsulated oligosaccharides using whole transcriptional profile analysis. The AIEC-infected mice model was used to investigate the protective effect of exosomes encapsuled HMOs.

## 2. Materials and Methods

### 2.1. Milk Samples and Subjects

Colostrum and mature milk samples were obtained from 20–50 mothers’ donation for scientific research purpose. Briefly, colostrum (within 4 days) and mature milk samples (within 1–6 month) have been collected from healthy mothers after delivery in local milk bank. All of the samples came from healthy mothers who had vaginal deliveries of healthy, normal-weight infants at full term. The colostrum samples were frozen in maternity unit at −80 °C. The mature milk was got at home and delivered to the laboratory with cold chain (4 °C), and then it was centrifuged (300× *g*, 4 °C, 30 min) to remove cells and kept at −80 °C within 24 h after sampling.

### 2.2. Nanosight Tracking Analysis on Human Milk Microparticles

The milk samples were firstly centrifuged (3000× *g*, 10 min, 4 °C) to remove the supernatant, and spun again (16,500× *g*, 30 min, 4 °C) to remove the cell debris, and passed through a filter (0.8 μm) for next step analysis. The Nanosight^®^LM10 nanoparticle characterization system employed a blue laser (405 nm) to illuminate and real-time characterize the milk vesicles. The filtered samples were diluted (1:1000) in PBS and 200 μL of particle samples were loaded for concentration measurement through NanoSight^®^ LM10.

### 2.3. Dynamic Analysis Human Milk Microparticle Population

NanoView was a Beckman Coulter (MoFlo XDP, Propel Lab, Boston, MA, USA) cell sorter that integrated with forward scatter detector (FSC). The design of NanoView have improved the capability of optical and electrical systems over the standard FSC diode or PMT, and extended the detection range below than 100 nm particles. Briefly, NanoView equipped MoFlo XDP cell sorter firstly calibrated the FSC resolution with the different sized fluorescent beads (Bangs Laboratories Inc., Fishers, IN, USA, Dragon Green Beads) and adjusted FSC and SSC-related settings (voltage and threshold). The Fluoresbrite size range beads (including 100 nm, 200 nm, 500 nm, 750 nm, Polysciences Inc., Warrington, PA, USA, Kit I (catalog # 21636)) were used to calibrate along different size ranges for uniformity and normalize NanoView’s functionality [26]. The beads were diluted with working buffer (PBS/0.1% Tween-20 solution) and prepared standard final concentration (1.29 × 107 beads/mL). Fluorescence was acquired on channel FL1 to calibrate bead populations. The setting of detection parameters was setup according to equipment protocol (Coherent Sapphire 488–200 Laser, 100 mW; FSC threshold: 0.01%; Drop Drive: 15 V approx; 94 K Frequency (dependent on Intellisort II); FSC 270 V, SSC 440 V). Milk samples were prepared as previously mentioned for Nanosight and then diluted with PBS (1:40 ratio, 400 μL/sample) to determine the microparticles size range. About 1 million particles per sample were tracked in each running analysis.

### 2.4. Isolation of Exosome-like Vesicles by Differential Ultracentrifugation

Exocellular vesicles isolation has performed as previously described by serial ultracentrifugation [12] with some modifications. Whole colostrum or mature milk supernatants were sequentially subjected to the first spin (3000× *g*, 4 °C, 15 min, 1.2 μm filters) and followed the second-round centrifuge (13,000× *g*, 4 °C, 30 min, 0.8 μm filters) and filtration (Advantech MFS, Dublin, CA, USA) to remove cell debris. The supernatants were performed the third-round centrifuge (16,500× *g*, 30 min, 4 °C) to further remove cell debris. The final super-centrifuge (120,000× *g*, 90 min, 4 °C) was run to pellet the exocellular vesicles. The pellet was washed twice and re-suspended in PBS and left overnight at 4 °C to dissolve for next step functional assay, or rinsed twice with Milli-Q water and dissolved in Milli-Q water.

### 2.5. Profile of Milk Exosome Encapsulated Oligosaccharides (MECO) Determined by LC-MS

Isolated colostrum/mature milk exosomes (from 1 mL milk) were dissolved in Milli-Q water (1.0 mL) and reduced with an excess of NaBH4 (25 °C, for 16 h). The reaction was terminated by glacial HAc addition in dropwise manner and samples were evaporated under nitrogen. Washed two rounds with MeOH/AcOH 95/5 and rinsed with an additional MeOH (100%) twice, samples were dissolved in Milli-Q water (2 mL) and desalted with DOWEX-50W resin column (25 mL, Sigma, St Louis, MO, USA), which had been prewashed with 1 M HCl. Each fraction (3 mL) was tested by LC-MS (Agilent 1100 HPLC coupled with ESI-TOF 6220 mass spectrometer, Agilent Technologies, Santa Clara, CA, USA). LC separation was performed on a graphitized carbon stationary phase (Hypercarb, 100 × 2.1 mm, Thermo Fisher Scientific, Waltham, MA, USA) and eluted with mobile phase of H_2_O (A) at a gradient of AcCN (B)—(flow rate of 0.20 mL/min, elution procedure listed as follows: 0% B, 0.0~2.0 min; 5% B, 2.0~6.0 min; 5% B, 6.0~9 min; 5~12% B, 9.0~20.0 min; 12% B, 20.0~22.0 min; 12~20%B, 22.0~26.0 min; 20~50% B, 26.0~32.0 min; 50~90% B, 32.0~35.0 min; 90% B, 35.0~40.0 min; 90~100% B, 40.0~41.0 min). Each injection was followed with 10 min equilibration at 0% B. Ion spray voltage was 3500 V and the gas temperature was 350 °C. The fragment of voltage was set at 70 V. Data were acquired and performed in the negative mode starting after 6 min elution, covering the *m*/*z* range 350–2300 at a 1.03 spectrum/s scan rate.

### 2.6. Colostrum Exosomes Labeled with PKH26

The isolated human colostrum exosomes were resuspended in PBS and purified by ultra-centrifugation (120,000× *g*, 70 min, 4 °C). The exosomes were labeled with PKH26 (red, Fluorescent Cell Linker Kit, Sigma-Aldrich, St Louis, MO, USA) for General Cell Membrane Labelling according to the manufacturer’s protocol and modified the washing process. Briefly, the exosomes were diluted in PBS before addition Diluent C (1 mL). PKH26 dye (4 μL) was added into Diluent C (1 mL) and mixed, and then added to the exosomes and the control. The samples were gently mixed (4 min, 4 °C) and 1% BSA (2 mL) was used to block the excess dye. The samples were then transferred to 300-kDa Vivaspin filters (Sartorius Stedim Biotech GmbH, Goettingen, Germany) and centrifuged (4000× *g*, 4 °C). Before being transferred to new 300 kDa Vivaspin filters, the samples were washed 3 times with PBS (5 mL/time) and rinsed twice with IMDM (5 mL, Sigma-Aldrich).

### 2.7. THP-1 Differentiated Macrophages Culture

The THP-1 (human monocytic cell line, American Type Culture Collection, Manassas, VA, USA) was cultured in RPMI 1640 media (contained with 2 mM L-glutamine, 100 U of penicillin/mL, 100 mg of streptomycin/mL, 25 mM HEPES) (C-RPMI) and 5% fetal bovine serum, Gibco BRL, Gaithersburg, MD, USA). THP-1 cells (5 × 105 to 106 per mL) were seeded in serum-free medium (SFM, Gibco BRL) and treated with PMA (200 nM, 48 h) for differentiation [27]. After incubation, washed cells with C-RPMI three times to remove non-attached cells by aspiration, and the adherent cells were cultured with fresh IMDM complete medium (10% FBS, penicillin (100 units/mL), streptomycin (100 μg/mL), L-glutamine (2 mM), sodium pyruvate (110 μg/mL) and GM-CSF (10 ng/mL, R&D Systems) for 2 days (37 °C, 5% CO_2_). To exclude the interference, the FBS was ultra-centrifuged and filtered (0.2 μM) prior to use to eliminate bovine serum exosomes. The well-differentiated macrophages were stained cellular plasma with CM-Dil and observed the morphology by fluorescence microscopy (Figure S2A). The TLR4 expression pattern of THP-1 (unstimulated) and macrophages were determined by immunofluorescence staining by anti-TLR4 antibody. The TLR4 expression in membrane was similar between THP-1 and differentiated macrophages (Figure S2B).

### 2.8. Macrophages Uptake of Labeled Colostrum Exosomes

For analysis the dynamic transport, endocytosis and subcellular co-localization of labeled colostrum exosomes into the macrophages, cells were planted in 6-well plates and Permanox Slides. The PKH26 labeled milk exosomes (from 1 mL colostrum) or PKH26-PBS (1 mL) control was put into THP-1 cells and incubated for 2 h at 37 °C or dynamic tracking the labeled exosomes uptake. The living cell plasma was stained with CellTrackerTM CM-Dil (Molecular Probe, C7000, Revere, MA, USA). The dynamic tracking was visualized by fluorescence microscope (Zeiss Axioplan 2, Carl Zeiss, Dublin, CA, USA). For subcellular co-localization analysis, after 2 h incubation with labeled exosomes, the THP-1 cells were rinsed twice with PBS, and then fixed with 4% formaldehyde (15 min, room temperature), and PBS rinsed twice, mounted with Vectashield (Vector Laboratories Inc., Burlingame, CA, USA), constrained with 3% of 7-ADD (BD Biosciences) for cell nuclei.

### 2.9. Milk Oligosaccharides 2′-FL Labeling

The milk oligosaccharides (2′-FL) were labeled with fluorescent dye 2-AB by reductive amination as described in the previous method with modification [28,29]. Briefly, a proper amount of 2′-FL was labeled with GlycoProfile™ 2-aminobenzamide (2-AB) Labeling Kit (Sigma-Aldrich, St Louis, MO, USA), enriched by GlycoProfile Glycan Clean-up Cartridges (Sigma-Aldrich, St Louis, MO, USA), and calibrated by normal phase HPLC (NP-HPLC) (Shimadzu prominence, Kyoto, Japan) on an Amide 80 column. The purified 2-AB labeled 2′-FL was determined by Waters MALDI MX Tof MS, and using 2-AB labeled dextran ladder standard that containing glucose unit (GU) oligomers and free 2′-FL as control. The correlation standardized curve of 2-AB labeled 2′-FL fluorescence intensity and 2′-FL’s concentration was determined by microplate reader (excitation at 330 nm and emission peak at 420 nm) (Figure S3A). The stability of 2-AB labeled 2′-FL in the buffer is tracking for 64 h in vitro.

### 2.10. Internalization of Labeled 2′-FL into Macrophages and Subcellular Localization

The labeled 2′-FL treated macrophages and dynamic tracked the 2-AB fluorescence intensity in the living macrophages at different time points. The stability of 2′-FL in the cellular was tracking for 64 h by detection the 2-AB fluorescence intensity (Figure S3B). To localize the endocytosis of 2′-FL into subcellular, the macrophage subcellular fractions, including nuclear extract, mitochondrial, cytosol and membrane, were isolated by using the NE-PER Nuclear and Cytoplasmic Extraction Reagents (Pierce). The fluorescence intensities of 2-AB labeled 2′-FL in the subcellular fractions were determined by microplate reader. The line regression standard curve of fluorescence intensity and concentration of 2-AB labeled 2′-FL was built according to the in vitro experiments (Figure S3A).

### 2.11. Colostrum Exosome Encapsulated Oligosaccharides Treated THP-1 Derived Macrophages

The well condition macrophages (about 1 × 106 cell/well) were cultured in 6-well plates, whereupon received the extracted colostrum exosomes encapsulated oligosaccharides (CECO) (from 10 mL original colostrum) or PBS alone. Incubated with an additional 2 days, the designated wells were removed the medium and washed with PBS twice. Cells were harvested for next step of transcriptome expression profile analysis.

### 2.12. Whole Transcriptome Expression Profiling of Macrophage

RNA was extracted from control and colostrum exosomes encapsulated oligosaccharides (CECO) treated macrophages using TRIzol reagent (Invitrogen, Carlsbad, CA, USA). The quality and quantity of extracted RNA was tested by spectrophotometric analysis and Bioanalyzer (Agilent Technologies, Santa Clara, CA, USA). An amount of 1 μg of RNA of each sample was employed for whole transcription profile labeling via a two-round amplification protocol. Expression profiles were determined using 4.5 μg of fragmented, labeled and hybridized with per Chip (Human Gene whole transcript 1.1 ST Arrays, Affymetrix). The expression data were normalized to the median expression of internal control probe set across all samples by RMA preprocessing protocol. The whole transcriptional profiles were calibrated and normalized, background-corrected and log2-transformed for parametric analysis. Raw cell data were annotated as MIAME format and submitted to Gene Expression Omnibus (GEO; http://www.ncbi.nlm.nih.gov/projects/geo/, accessed on 30 June 2021; accession GSE163125). All internal control genes were removed and the remaining genes probe values were imported into the Affymetrix Power Tools software (APT package). Significantly differentially expressed genes were screened by using SAM (significance analysis of microarrays) with the R package ‘samr’ (false discovery rate (FDR) < 0.05; fold change > 2). The significant genes were identified by filtered condition and generated two-dimensional hierarchical clusters map. To identify key differentially expressed networks, the GeneGo MetaCore™ Pathway Analysis was employed to explore the annotation of significant changed genes interactions from the intact repository.

### 2.13. In Vivo Animals Experiments

Briefly, the C57BL/6 mice (eight-week-old) were purchased from Hunan SJA Laboratory Animal Co. Ltd., People’s Republic of China (license no. SCXK (Xiang) 2019-0004, Changsha, Hunan). Mice were raised in temperature-controlled room (24  ±  1 °C, 50~60% relative humidity) with 12-h light-dark period switch, and acclimatized to the laboratory environment for 7 days, feeding with standard diet and water, before the experiment. A murine model of AIEC infection [30,31] was adapted and validated. All experimental protocols were reviewed and approved by the Committee of Institutional Animal Care & Usage, Kunming Institute of Botany, Chinese Academy of Sciences (Permit No. KIB-R-018). Experiments were performed in the animal facility accordance to the research guide of Institutive Lab Research Animal Care & Usage. Mice have treated with 2.5% dextran sodium sulfate (DSS) in their drinking water for 3 days, and administrated with of streptomycin (20 mg, day 4) by gavage to disrupt mouse microbiota. Half were also received colostrum exosomes encapsulated oligosaccharides (CECO) (extracted from 10.0 mL original colostrum) or 2′-FL (100 mg, dissolved in 200 μL) by gavage for each of the 4 days. The two groups of experimental mice were inoculated with AIEC (109 colony forming unit (CFU), 200 μL) via gavage on the 5th day and sacrificed after another 4 days. In the control group, DSS and antibiotics were given, but only a sham PBS inoculation, to the mice. Body weight was monitored daily. After experiments, animals were sacrificed and the length of the colon was measured.

### 2.14. Hematoxylin & Eosin Staining

The mice colons were fixed by formalin (4%), paraffin-embedded and sectioned as 5 mm slices by standard immunohistochemistry (IHC) protocol. The slices were stained with H&E and mounted. The histology inflammation score of section were evaluated according to the standard (value from 0 to 3.0 represents the without inflammatory; 1 means increased present inflammation; 2 represents infiltrates in submucosa; 3 means transmural) [32]. The results of stain slides were observed and evaluated.

### 2.15. Statistical Analysis

Data are presented as Means ± SEM. The significance of differences for the experimental values were compared using the student *t*-test through Prism software (GraphPad Software, Inc., San Diego, CA, USA). *p*-value less than 0.05 was defined as a significant difference.

## 3. Results

### 3.1. Characterized the Dynamic Profile of Human Milk Exosomes

**Figure 1:**
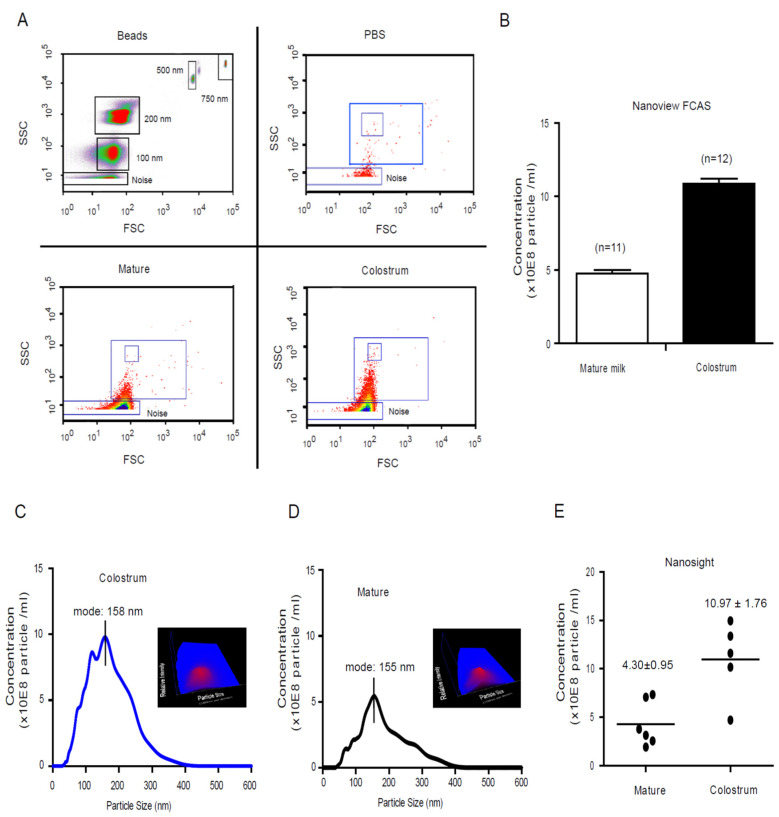
The dynamic profile of human milk Exosome. (**A**) NanoView identified the milk exosome size distribution area with standard beads. With need for the separation of a mixture containing 200 and 500 nm latex beads, the NanoView is capable of separating a mixture of 100–500 nm beads into distinct populations. The gating strategy for these experiments to determine instrument and background noise are described in the methods section. (**B**) The dynamic profile of vesicle size and concentration of human colostrum and mature milk were determined by NanoSight^®^ LM10. Size mode: 100 nanometers (nm; 3 technical replicates). The results represent the exosomes concentration by NanoSight^®^ analysis showing the number of exosomes per 1 mL of milk derived from different batch healthy donors’ colostrum (**C**) (*n* = 6) and mature milk (**D**) (*n* = 5). (**E**) The comparison of microparticle concentration of colostrum and mature milk.

The natural features of milk exosomes were discovered using two novel exosomal dynamic tracking technologies: NanoSight^®^ nanoparticle tracking analysis system and Nanoscale flow cytometry analysis (termed NanoView module). These technologies had the advantage of detecting live particles with diameter ranging from 100 nm to 750 nm with minimal impact on the EVs size and distribution of EVs. The size of milk exosomes arrange was recognized and sorted into a gated area corresponding to a size of 100–200 nm (Figure 1A), with particle concentrations of 10.65 ± 0.65 × 10^8^ particle/mL (*n* = 12, colostrum) and 4.87 ± 0.35 × 10^8^ particle/mL (*n* = 11, mature milk), respectively (Figure 1B). Using NanoSight to investigate the exact size distribution of the milk vesicle, the size range of colostrum and mature milk exosomes was determined to be 158 ± 65 nm and 155 ± 71 nm in diameter, respectively (Figure 1C,D). The exosomes concentrations in colostrum and mature human milk are 10.97 ± 1.76 (*n* = 6) and 4.30 ± 0.96 (*n* = 5) (×10^8^ particle/mL, *p* < 0.001), respectively (Figure 1E). Microparticle concentrations in colostrum were significantly higher than that in mature milk. The diameter of live milk exosomes are about 160 nm, bigger than previously determined milk exosomes (diameter sizes around 50–80 nm), which may be caused by the multiple steps filtering, higher ultracentrifuge g-force and measured size under packed pellets [33].

### 3.2. Characterization Profile of Human Milk Exosome Encapsulated Oligosaccharides by LC-MS

**Figure 2:**
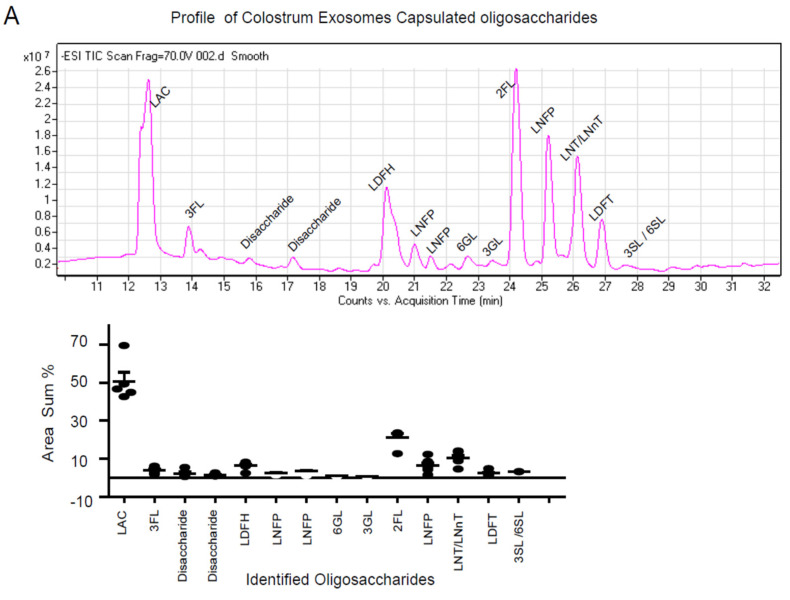

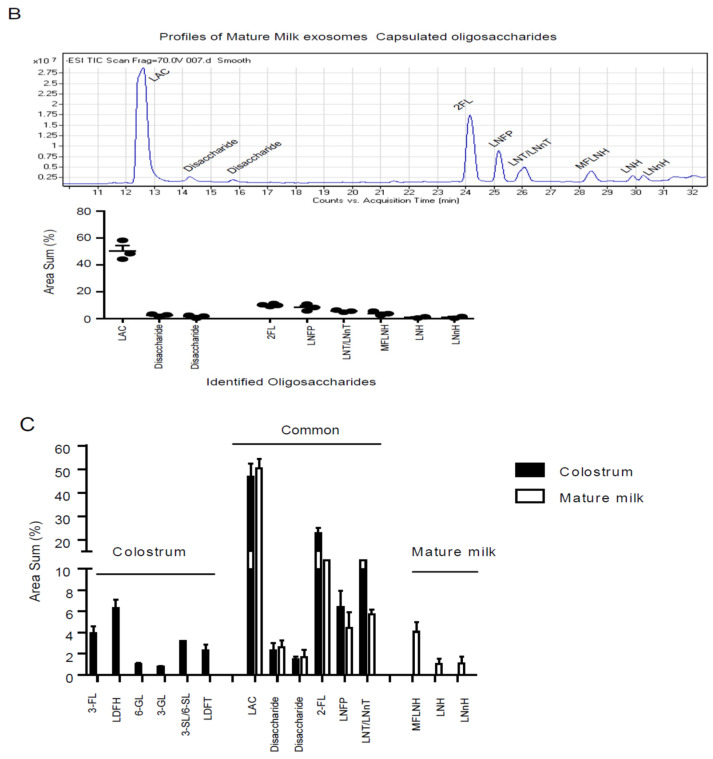
Characterization of human colostrum exosome oligosaccharide profile determined by LC-MS (high-performance liquid chromatography with tandem mass spectrometry). Representative exosome contained profile of HMOs from colostrum (**A**) and mature milk (**B**) were determined by LC-MS. Major peaks were identified by comparing retention times and mass spectra with authentic isolated standards. The variations in relative amounts (%) of the major individual oligosaccharides isolated from colostrum (*n* = 5) were determined. The comparison of exosomes encapsulated oligosaccharide profile between colostrum and mature milk (**C**). Annotations for oligosaccharides abbreviations: Lactose (LAC); 2-fucosyllactose (2′-FL); 3-fucosyllactose (3-FL); Lacto-N-difucohexaose (LDFH); Lacto-N-fucopentaose I (LNFP I); Lacto-N-fucopentaose II (LNFP II); Lacto-N-Tetraose (LNT); Lacto-N-Fucopentasose (LNFP); Lacto-Difucotetraose (LDFT); 3-galactosyllactose (3-GL); 6-galactosyllactose (6-GL); 3-Sialyllactose (3-SL); 6-Sialyllactose (6-SL); Lacto-N-Fucopentaose I (LNFP I); Monofucosyl-lacto-N-hexaose (M FLNH); Lacto-N-Hexaose (LNH); Lacto-N-neohexaose (LNnH).

To characterize the profile of encapsulated oligosaccharides in milk exosomes, super-centrifugation was used to isolate milk exosomes (Figure S1), and oligosaccharides were extracted and identified by LC-MS. The characteristic pattern of HMOs in the colostrum exosomes was investigated and 14 typical oligosaccharides were discovered (Figure 2A), including 2-fucosyllactose (2′-FL), 3-fucosyllactose (3-FL), lacto-N-difucohexaose (LDFH), 6-galactosyllactose (6-GL) and 3-Sialyllactose (3-SL) (Figure 2C, Table 1). Individual oligosaccharides variation percentages were determined based on the levels of individual oligosaccharides each of the five batches (Figure 2A). In parallel, the oligosaccharides profile of mature milk exosomes was also investigated (Figure 2B, Table 1), and it was discovered that there were less varieties of individual oligosaccharide (Figure 2C). When comparing the oligosaccharides profiles of colostrum and mature milk exosomes, colostrum exosomes encapsulated more types of oligosaccharides (14 types) as well as six galactosyl-oligosaccharides peaks (3-FL, LDFN, 6-GL, 3-GL, 3-SL/6SL and LDFT) (Figure 2C) that had previously been found only in colostrum [2], which are characterized with immunomodulatory function [34,35]. Colostrum milk exosomes contained these immunomodulation oligosaccharides provide a unique molecular basis for colostrum’s immunomodulation function. Six subtypes of oligosaccharides have been identified as common encapsulated oligosaccharides in exosomes of colostrum and mature milk, including LAC, Disacharide, 2′-FL, LNFP and LNT (Figure 2C).

### 3.3. Colostrum Exosomes Capsulated Oligosaccharides Phagocytosis into Macrophages

**Figure 3:**
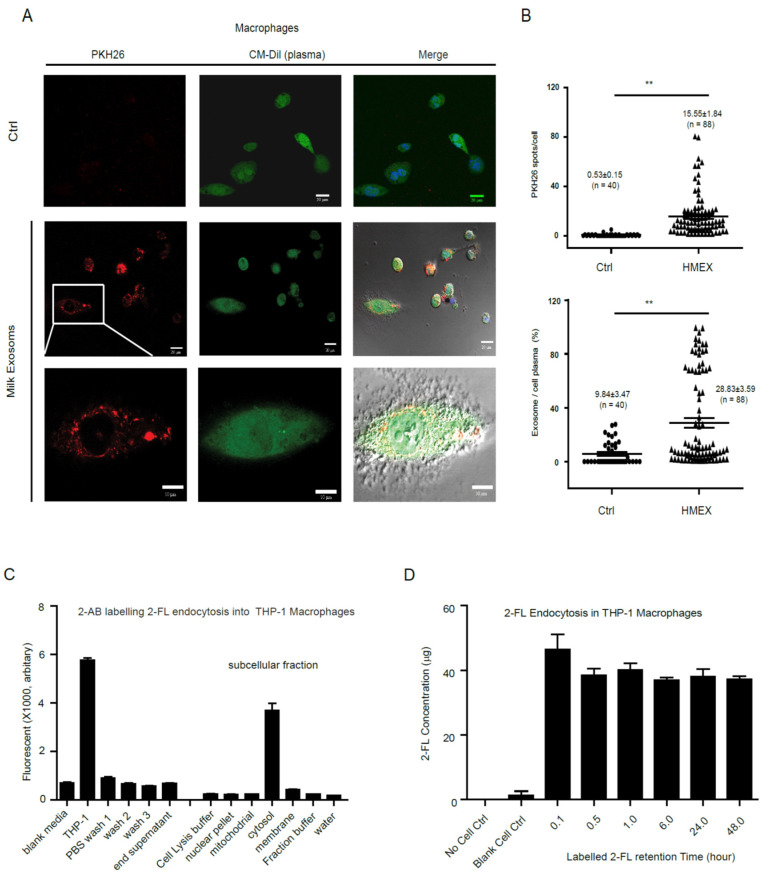
Human milk exosomes endocytosis into cytosol of macrophages. Isolated milk exosomes were labeled with PKH26 (from 1 mL of colostrum) and added into THP-1 differentiated macrophages at 37 °C. The macrophages cellular plasma was stained with CM-Dil and cells were dynamic tracking by fluorescence microscopy (**A**). The 2-AB labeled 2′-FL mainly distributed in the cytosol fraction of macrophages (**B**). The 2-AB’s fluorescence intensity of 2′-FL distributed in the cytosol fraction of macrophages (**C**) and maintained stable over 48 h (**D**). ** *p* ≤ 0.01.

To explore how colostrum exosomes encapsulated oligosaccharides were transferred into recipient cells, colostrum exosomes were isolated and labeled with PKH26 (red), and the macrophages were live stained with CM-Dil (plasma, green) to track the dynamic process of exosomes phagocytosis into cells. The labeled colostrum exosomes were rapidly endocytosed into macrophages (15.55 ± 1.84 PKH26 spot/cell, *n* = 88, **, *p* < 0.001), and mainly distributed into cytosol and well co-localized with plasma area (ratio of exosomes/cell plasma, 28.83 ± 3.59 %, *n* = 88, **, *p* < 0.001) (Figure 3A,B), and stable over 48 h, as shown in the images and video (Support video). At the same time, macrophages were treated with specific glycan dye 2-AB labeled 2′-FL, the major active component of oligosaccharides and contained in the exosomes, and the distribution of 2-AB’s fluorescence intensity in different subcellular fractions was detected at different time points. The results indicated that the 2-AB fluorescence intensity was mostly concentrate in the cytosol fraction (Figure 3C). Furthermore, the fluorescence intensity of 2-AB’s labeled 2′-FL was primarily distributed in the cytoplasm fraction of macrophages and remained constant for 48 h (Figure 3D).

### 3.4. Colostrum Exosomes Capsulated Oligosaccharides (CECO) Modulate Macrophages Alternative Activation and Mucosal Immunity Development 

**Figure 4:**
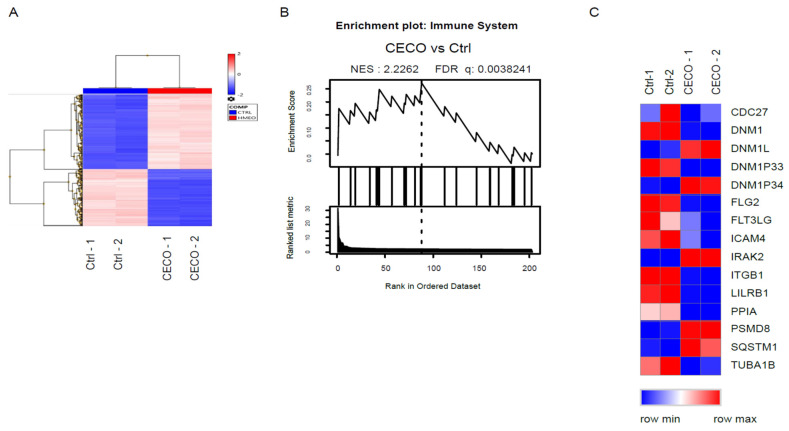

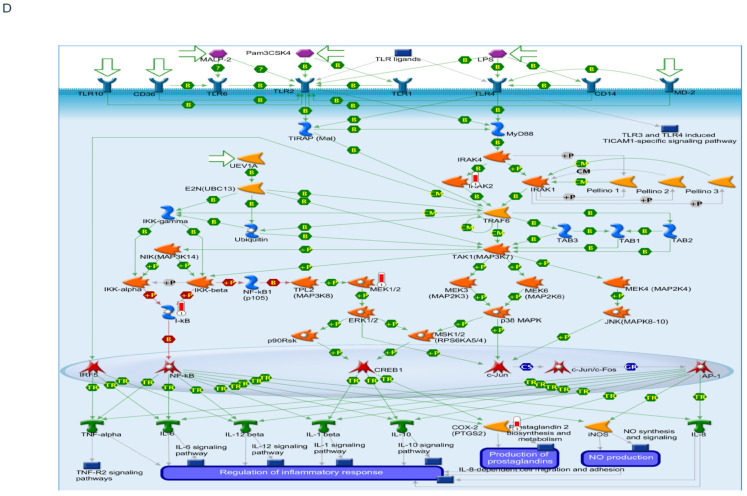
The Canonical process networks and signaling pathways in macrophages significantly modulated by colostrum exosome encapsulated oligosaccharides. The hierarchical cluster of significantly changed genes in macrophages modulated by colostrum exosome encapsulated oligosaccharides (**A**). The significantly changed genes in macrophages modulated by colostrum exosome encapsulated oligosaccharides were enriched in immune system function, as accessed by GSEA (Enrichment Score (ES)), Normalized Enrichment Score (NES), False Discovery Rate (FDR) (**B**). Heat maps comparing expression of significantly genes that involving immune system function (3-fold cutoff, *p* < 0.001) (**C**). Colors represent genes up-regulated (red) and down-regulated key genes (blue). The scored network of “Immune response_TLR2 and TLR4 signaling pathways” modulated by colostrum exosome contained oligosaccharides on macrophages (**D**). It is based on the enrichment distribution sorted by ‘Statistically significant Maps’ set. Related experimental data is linked to and visualized on the maps as thermometer-like figures. Upward thermometers have red color and indicate significantly up-regulated signals and downward (blue) ones indicate down-regulated expression levels of the genes.

To investigate the immunological regulatory effect of milk exosomes encapsulated oligosaccharides on macrophages, CECOs were extracted from the collected colostrum exosomes and treated macrophages for 48 h. The significantly regulated gene expression profile and key pathways were discovered using whole transcriptome microarray. After data normalization and significant Analysis filtering (Fold change > 3.0 or <−3.0, *p* < 0.001), a total of 2753 genes were identified as significantly changed (up-regulated 1186 genes; down-regulated 1567 genes, Table S1) and computationally clustered (Figure 4A). To explore the key involving functions that CECOs regulated, the significant changed genes were calculated and evaluated through gene set enrichment analysis (GSEA). The top ten functional pathways that were significantly regulated (*p* < 0.05, Table 1) have been summarized. The immune system was highlighted as the most highly regulated function (Table 1), with up-regulated (DNM1L, DNM1P34, IRAK2, PSMD8 and SQSTM1) and down-regulated key genes (CDC27, DNM1, ITGB1, FLT3LG, ICAM4, PPIA, TUBA1B) clustering together (Figure 4B,C). The toll-like receptor signaling associated with MyD88 and chemokine signaling was also highlighted (Table 2), which involved the innate immunity transformation to adoptive immunity, and matched the immune modulated functions of HMOs discovered in previous study [2].

Furthermore, using MetaCore GeneGo, which examines within known canonical pathways and functional networks, the functional process networks affected by colostrum exosomes encapsuled oligosaccharides were computationally grouped. The top ten process networks were summarized, and the major components of process networks and regulatory aims of macrophages changed by CECOs were discovered (Supplemental Table S2). The biological process networks primarily focused on inflammation and immune response included TREM1 signaling, NK cell cytotoxicity, IL-4 signaling, Neutrophil activation, IL-6 signaling, and Amphoterin signaling. 

The top ten of canonical pathway maps were summarized, including the pathways of apoptosis and survival _TNFR1 signaling, TNF-family pathway, Granzyme B signaling, anti-apoptotic of TNFs/NF-kB/Bcl-2, role of IAP-protein in apoptosis, IGF family signaling in colorectal cancer, cytoskeleton remodeling, immune response_TLR2 and TLR4 signaling. These pathways primarily involve the innate immune response, producing a large number of pro- and anti-inflammatory cytokines and chemokines, activating selective apoptosis, and mediating antigen presentation to the adaptive immune system (Supplemental Table S2). In immune regulatory cells such as macrophages, neutrophils and monocytes, the immunological response of phagocytosis represents a central component of innate immune response, and it involved numerous essential signaling molecules such as NF-kBIE, ILT2, I-kB in the regulatory network (Supplemental Table S3). The GeneGo analysis revealed a critical immune regulatory functional network, which includes the immune response TLR2 and TLR4 signaling pathway in the macrophages that are strongly modified by CECO (Figure 4D). This network includes TLR2, TLR4, I-kB, COX-2 and MEK1/2, which not only play a role in immune response related inflammation, but also activate the external particle internalization and phagosome formation through a variety of signaling pathways that together orchestrate re-arrangement of the actin cytoskeleton, alterations in membrane trafficking and blebbing. Significantly changed genes were sorted and visualized on the network as thermometer-like figures. Upward thermometers (red) demonstrate up-regulated signals, whereas downward (blue) imply down-regulated genes expression levels.

### 3.5. Colostrum Exosome Enapsulated Oligosaccharides Attenuate AIEC Infection and Inflammation In Vivo

**Figure 5:**
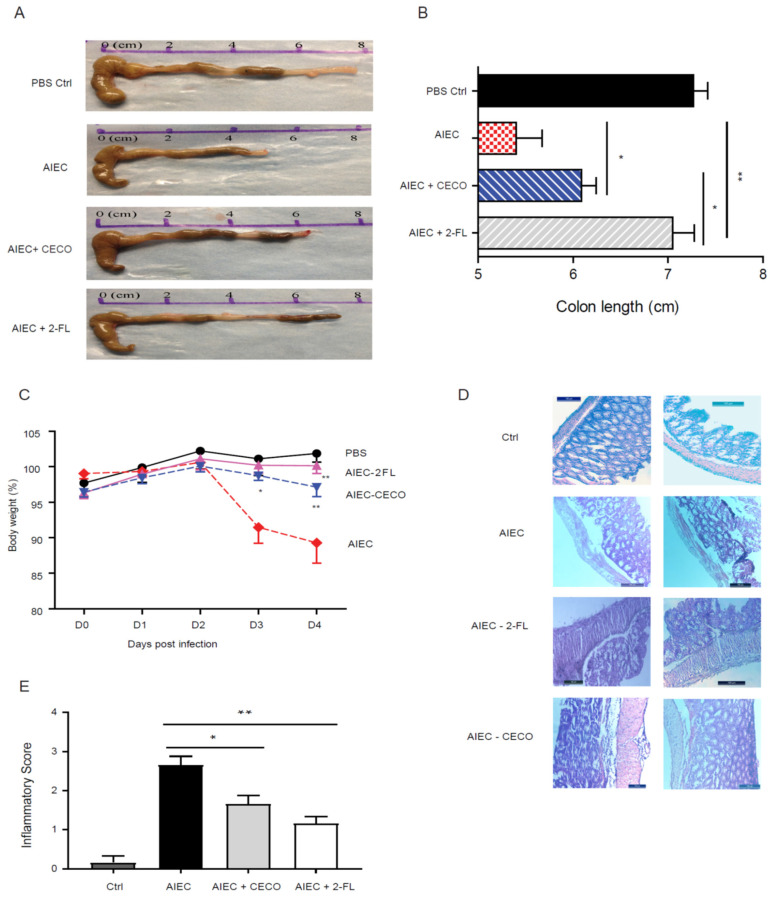
Human colostrum exosome oligosaccharides (CECO) attenuate inflammation in adherent-invasive *E. coli* (AIEC) infected mice. C57BL/6 mice received 0.25% dextran sodium sulfate (DSS) in their drinking water for 3 days, and were given 20 mg of streptomycin by gavage on day 4; two group mice also received 100 mg of 2′-FL/CECOs in 200 μL by gavage for each of the 4 days, respectively. On the 5th day, the two groups of experimental mice were inoculated with 10^9^ colony forming unit (CFU) AIEC via gavage and sacrificed after 4 days; a control group received DSS and antibiotic, but only a sham PBS inoculum. The comparison of reduction in colon length caused by AIEC infection did not occur with CECOs pretreatment (**A**,**B**). The body weight loss that follows AIEC infection did not occur with 2′-FL and CECOs pretreatment (**C**). Comparison of H&E stained colon sections image (**D**) and inflammation score (**E**) from AIECs infected mice without or with CECOs and 2′-FL pretreatments. Representative images from six mice. Bars = 100 μm. Mean ± SEM, *n* = 6 for all groups; * *p* ≤ 0.05; ** *p* ≤ 0.01 by analysis of variance.

The immunological regulatory effect of colostrum exosomes encapsulated oligosaccharides on the intestinal mucosal immune system was verified using the DSS-mice model of AIEC infection. Extracted colostrum exosomes encapsulated oligosaccharides (from 10 mL colostrum) and 2′-FL (100 mg, positive control) were gavaged once per day for 4 days before to inoculation and significantly prevented the mice’s colon shortening (Figure 5A,B) and body weight loss (Figure 5C). In parallel, the AIEC infected mice had significantly reduced colons and a sharp drop of body weight in D3 & D4. Colon lengths were similar to normal in AIEC inoculated mice that were pretreated with 2′-FL, while colostrum exosomes encapsulated oligosaccharides pretreatment significantly recovered the colon length from AIEC inoculated mice (Figure 5B). Furthermore, H&E colon staining results revealed epithelial cell sloughing, immune cell infiltration and muscularis mucosa hyperplasia, whereas exosomes HMOs pretreated mice’s colons showed fewer of these inflammatory symptoms (Figure 5D,E).

## 4. Discussion

In this study, the arm is to test the hypothesis that exosomes are the natural manner for HMOs to get into immune cells and regulate infant’s mucosal immunity development. Mother’s milk has been approved as natural nutritional resource for newborns as well as active immunological regulatory components that protect against bacteria and virus infection and modify the development of mucosal immunology system [1,2,14]. Mother’s colostrum and mature milk contains rich microparticles (exosomes), whereas colostrum contains more concentrated active substances [2,12]. Exosomes are involved in a wide range of physiological processes and activities, including immunological regulation, nutrition transportation, and so on [9,36]. Many active components, proteins (MHC classes I and II, CD63, CD81, and CD86), mRNA and miRNA have been characterized in the milk’s exosomes [12,37]. Human milk oligosaccharides (HMOs) are non-digestible glycans that contain glucose, galactose, N-acetyl-glucosamine, fucose and sialic acid and are hypothesized to have with prebiotics and immune regulatory effects [2,13]. HMOs are soluble and free in milk, and they are absorbable and/or translocatable into infant’s cells or gastrointestinal lumen and tract, and subsequently circulate into plasma and urine, according to a widely accepted hypothetical model [1,13]. Little direct evidence exists for the natural state of oligosaccharides in milk, as well as their absorption or translocation into cells. Milk exosomes’ dynamic features, as well as the profiles of milk exosomes encapsuled oligosaccharides profiles were characterized in this study. It was discovered that colostrum exosomes contained more active milk oligosaccharides than mature milk exosomes when the profiles of HMOs encapsulated in exosomes from colostrum and mature milks were compared. Some distinct fingerprint oligosaccharides were discovered in exosomes from colostrum and mature milks (Figure 2C). Colostrum contains more types of oligosaccharides than mature milks, which is consistent with previous research [2]. It is possible that colostrum oligosaccharides play an important function in immunological regulation. 

**Figure 6:**
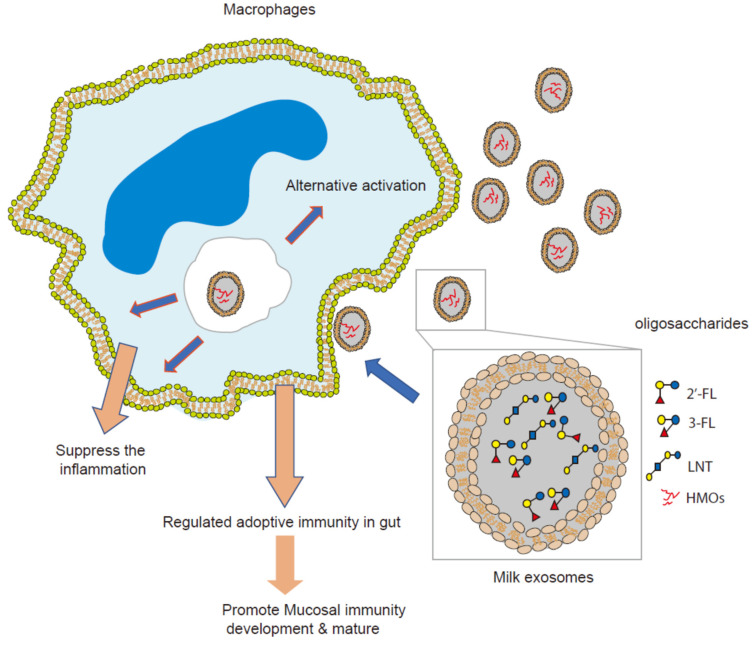
A graphic of HMOs via exosomes as vector to modulate macrophages’ alternative activation and help to remodel the infant’s mucosal immunity.

The intestinal macrophages play an increasingly important role in intestinal homeostasis and immune system development. Intestinal macrophages are heterogeneous in the gut wall and phagocytic to eliminate invading microorganisms while avoiding an overt inflammatory reaction in both humans and mice [38,39]. On their surfaces and in vacuolar and cytosolic compartments, intestinal macrophages express typical PRR, such as Toll-like receptors (TLRs) (TLR2, TLR4), RIG-I-like and Nod-like sensing receptors [22,23]. Alternative phenotype macrophages are characterized by expression or secretory mediators via PRRs activated, including chemokines, anti-bacterial and proteolytic enzymes, and anti-inflammatory cytokines, such as IL-4 and IL-6, IL-10, IL-1R antagonist, and production of growth factors, such as vascular endothelial growth factor (VEGF) and insulin-like growth factor (IGF)-1 [22,24,25]. The immune regulatory effect of HMOs on infant interest mucosal immunity is widely accepted and verified by multiple independent investigations [1,2,3,4]. In previous studies, the role of HMOs in interest mucosal immunity regulation and development were systematically investigated through the immature human intestinal ex vivo model. The essential signaling regulatory networks controlled by colostrum oligosaccharides and enriched on the growth and maturation of an infant’s intestinal mucosal immunity have been identified. For neonatal intestinal mucosa, the HMOs immunological regulation model is well known [2]. HMOs could reduce LPS-induced inflammation in intestinal epithelium via deregulated the expression CD14, with 2′–fucosyllactose (2′-FL) identified as the major contributor and protecting the mice from AIEC infection [17]. However, it is unclear that the contribution of intestinal macrophages in HMOs immune regulatory effect in the intestinal mucosa. In this study, THP1 differentiated macrophages were employed as a model to assess the immunological regulation mechanism of HMOs on intestinal macrophages. Colostrum exosomes containing oligosaccharides were phagocytosed by macrophages and altered the whole transcriptome, allowing macrophages to switch to the alternate activation phenotype. Several common feature process networks have been engaged, including the IL-4, IL-6, and TREM1 signaling pathways (Supplemental Table S2). When combined with previous cytokine profile analysis of colostrum HMOs stimulated on infant intestine tissue, which showed that HMOs via exosomes significantly increased the secretion of several cytokines involved in macrophage alternative activation, such as IL-10, IL-13, and IFA2 [2], it is strongly supported that HMOs via exosomes as vector to activate macrophages’ alternative activation and help to remodel the infant’s mucosal immunity (Figure 6). Dysbiosis of intestinal microbiome has been linked to inflammation bowel disease (IBD [40]. *Escherichia coli*, particularly adherent-invasive *E. coli* (AIEC), has been approved to associate to the pathogenesis of IBD [41,42]. We previously discovered that HMOS and 2-fucosyllactose (2′-FL) have anti-infective and anti-inflammation effect of in AIEC-infected mice model via suppressed CD14 [17]. In this study, mice infected with adherent-invasive *E. coli* (AIEC) as a model to evaluate the anti-infective and anti-inflammation activity of milk exosomes encapsulated oligosaccharides. Because 2-fucosyllactose was discovered to be a key component in the milk exosomes, it was employed as positive control. In the mice model, pretreatment with colostrum exosomes encapsuled oligosaccharides significantly reduced the inflammation generated by AIEC, significantly recovered the shorter colon length induced by inflammation, and significantly protected the colon tissue from inflammation-related damage. Exosomes from colostrum have been found to contain certain milk oligosaccharides, such as 2-fucosyllactose (2’-FL) and 3-Sialyllactose (3-SL), which have been proven to help reduce inflammation, modulate adoptive immunity, facilitate bacterial colonization and establish mucosal immunity [3,17]. These active oligosaccharides work together to decrease inflammation and protecting mice from AIEC infection.

## 5. Conclusions

In summary, we used dynamic technology to comprehensively characterize the properties of milk exosomes and the profile of human milk exosomes encapsulated oligosaccharides in this study. Furthermore, our results directly demonstrated that HMEOs delivered into macrophages via exosomes phagocytose and activate the alternative activation model to modulate signaling in intestinal mucosa immunity development. These findings revealed the natural transport of HMOs from mother to newborn and shed light on the role of the milk oligosaccharides in the development and maturation of infant’s mucosal protection and immunity.

## Figures and Tables

**Table 1:** The oligosaccharide profile of those capsulated in colostrum and mature milk exosomes.

Sample	Peak	Abbreviation	Full Name	Area Sum (%)
Colostrum exosomes	1	LAC	Lactose	46.85 ± 5.50
Colostrum exosomes	2	3-FL	3-Fucosyllactose	2.29 ± 0.71
Colostrum exosomes	3	Disaccharide	Disaccharide	1.45 ± 0.28
Colostrum exosomes	4	Disaccharide	Disaccharide	3.93 ± 0.66
Colostrum exosomes	5	LDFH	Lacto-N-difucohexaose	6.29 ± 0.82
Colostrum exosomes	6	LNFP II	Lacto-N-Fucopentaose II	2.55 ± 0.34
Colostrum exosomes	7	LNFP	Lacto-N-Fucopentaose	3.38 ± 0.63
Colostrum exosomes	8	6-GL	6′-galactosyllactose	0.91 ± 0.09
Colostrum exosomes	9	3-GL	3′-galactosyllactose	0.72 ± 0.07
Colostrum exosomes	10	2’-FL	2-Fucosyllactose	21.08 ± 2.09
Colostrum exosomes	11	LNFP I	Lacto-N-Fucopentaose I	7.43 ± 1.52
Colostrum exosomes	12	LNT/LNnT	Lacto-N-Tetraose	6.44 ± 1.43
Colostrum exosomes	13	LDFT	Lacto-Difucotetraose	2.32 ± 0.56
Colostrum exosomes	14	3SL/6SL	3′/6-Sialyllactose	3.18 ± 0.04
Mature milk exosomes	1	LAC	Lactose	50.30 ± 4.15
Mature milk exosomes	2	Disaccharide	Disaccharide	2.61 ± 0.65
Mature milk exosomes	3	Disaccharide	Disaccharide	1.69 ± 0.67
Mature milk exosomes	4	2-FL	2-Fucosyllactose	12.24 ± 0.47
Mature milk exosomes	5	LNFP I	Lacto-N-Fucopentaose I	8.43 ± 1.03
Mature milk exosomes	6	LNT/LNnT	Lacto-N-tetraose/Neotetraose	5.70 ± 0.45
Mature milk exosomes	7	M FLNH	Monofucosyl-lacto-N-hexaose	4.06 ± 0.90
Mature milk exosomes	8	LNH	Lacto-N-hexaose	1.05 ± 0.48
Mature milk exosomes	9	LNnH	Lacto-N-neohexaose	1.08 ± 0.64

**Table 2:** The top ten functional pathways significantly regulated in macrophages by colostrum exosome encapsulated oligosaccharides.

Description	Size	Leading Edge Number	ES	NES	*p*-Value	FDR
Immune System	22	13	0.4598	2.2262	0.0038241	0.11357
Human Thyroid Stimulating Hormone (TSH) signaling pathway	5	5	0.72976	1.9786	0.0039761	0.30352
Toll-like Receptor Signaling related to MyD88	5	5	−0.65919	−1.8344	0.012848	0.64332
Neural Crest Cell Migration during Development	7	6	0.55526	1.7762	0.015009	0.4129
Apoptosis Modulation and Signaling	7	7	−0.53998	−1.8065	0.018908	0.38316
Neural Crest Differentiation	11	7	0.45688	1.8374	0.018939	0.49888
Alzheimer’s Disease	7	7	0.5241	1.6724	0.025688	0.51279
Chemokine signaling pathway	14	14	0.36888	1.6563	0.033582	0.43128
Angiopoietin Like Protein 8 Regulatory Pathway	11	10	−0.39309	−1.5854	0.042735	0.78929
AMP-activated Protein Kinase (AMPK) Signaling	8	8	0.45753	1.5804	0.04717	0.50515
RAC1/PAK1/p38/MMP2 Pathway	6	4	0.53976	1.6085	0.048319	0.48671

## Data Availability

The microarray data generated or analyzed during this study were deposited in Gene Expression Omnibus (GEO; http://www.ncbi.nlm.nih.gov/projects/geo/, accessed on 30 June 2021; accession GSE163125), including original files and normalized data for next step analysis. Expression data were normalized, background-corrected and log2-transformed for parametric analysis.

## Supplementary Materials

The following are available online at https://www.mdpi.com/article/10.3390/nu13093198/s1, Figure S1: The workflow of human milk exosome preparation and isolation; Figure S2: Normalization of THP-1 differentiated macrophages: Figure S3: Calibration of fluorescent intensity and the 2-AB labelled 2-FL concentration; Table S1: The significantly regulated genes by colostrum exosome capsulated oligosaccharides in macrophage; Table S2: Enrichment results of top ten Process Networks regulated by colostrum exosome capsulated; Table S3. Enrichment results of top ten Pathway Maps regulated by colostrum exosome capsulated oligosaccharides.

## Abbreviations

HMOs, human milk oligosaccharides; 2′-FL, 2-fucosyllactose; 3-SL, 3-Sialyllactose; AIEC, adherent-invasive E. coli; MECO, Milk Exosome Capsulated Oligosaccharides; CECO, Colostrum Exosome Capsulated Oligosaccharides; 2-AB, 2-aminobenzamide; LAC, lactose; LDFH, lacto-N-difucohexaose; 6-GL, 6-galactosyllactose.
