# Supplementary material for: Milk Exosomes Transfer Oligosaccharides into Macrophages to Modulate Immunity and Attenuate Adherent-Invasive E. coli (AIEC) Infection
Source: Nutrients. 2021 Sep 14;13(9):3198. doi: 10.3390/nu13093198 (PMC8472098; doi:10.3390/nu13093198)
Supplement: Supplementary file 1 [file nutrients-13-03198-s001.zip › nutrients-1299600-supplementary.pdf]

Milk exosomes deliver oligosaccharides into macrophages to modulate immunity and attenuate adherent-invasive E. coli (AIEC) infection

YingYing He[2], Zhicheng He[1,3], Serena Leone[4], Shubai Liu[1,*]

[1] State Key Laboratory of Phytochemistry and Plant Resources in West China, Kunming Institute of Botany, Chinese Academy of Sciences, Kunming, 650201 Yunnan, P. R. China;

[2] School of Chemical Science & Technology, Yunnan University, Kunming, Yunnan 650091, China;

[3] University of Chinese Academy of Sciences, Beijing 100049, China.

[4] Department of Chemical Sciences, University of Naples 'Federico II', Napoli, Italy.

***Corresponding author:**

Dr. Shubai Liu, State Key Laboratory of Phytochemistry and Plant Resources in West China, Kunming Institute of Botany, the Chinese Academy of Sciences, #132 Lanhei Road, Panlong District, Kunming, Yunnan 650201, China.

E-mail: liushubai@mail.kib.ac.cn

Supplementary Information

Supplementary Figure Legends

Figure. S1. The workflow of human milk exosome preparation and isolation.

Figure.S2. Normalization of THP-1 differentiated macrophages. The THP-1 differentiated macrophages by PMA (200 nM) was stained cellular plasma with CM-Dil dynamic tracking by fluorescence microscopy (A). Characterization of TLR4 expression in THP-1 differentiated macrophages (B).

Figure. S3 Calibration of fluorescent intensity and the 2-AB labelled 2-FL concentration. The standard curve of fluorescent intensity and 2-AB labelled 2-FL concentration in vitro assay system (A). The 2-AB's fluorescence intensity of 2-FL was stable over 64 hours in buffer (B).

Table S1. The significantly regulated genes by colostrum exosome capsulated oligosaccharides in macrophages.

Table. S2. Enrichment results of top ten Process Networks regulated by colostrum exosome capsulated oligosaccharides.

Table. S3. Enrichment results of top ten Pathway Maps regulated by colostrum exosome capsulated oligosaccharides.

Sup. Fig 1

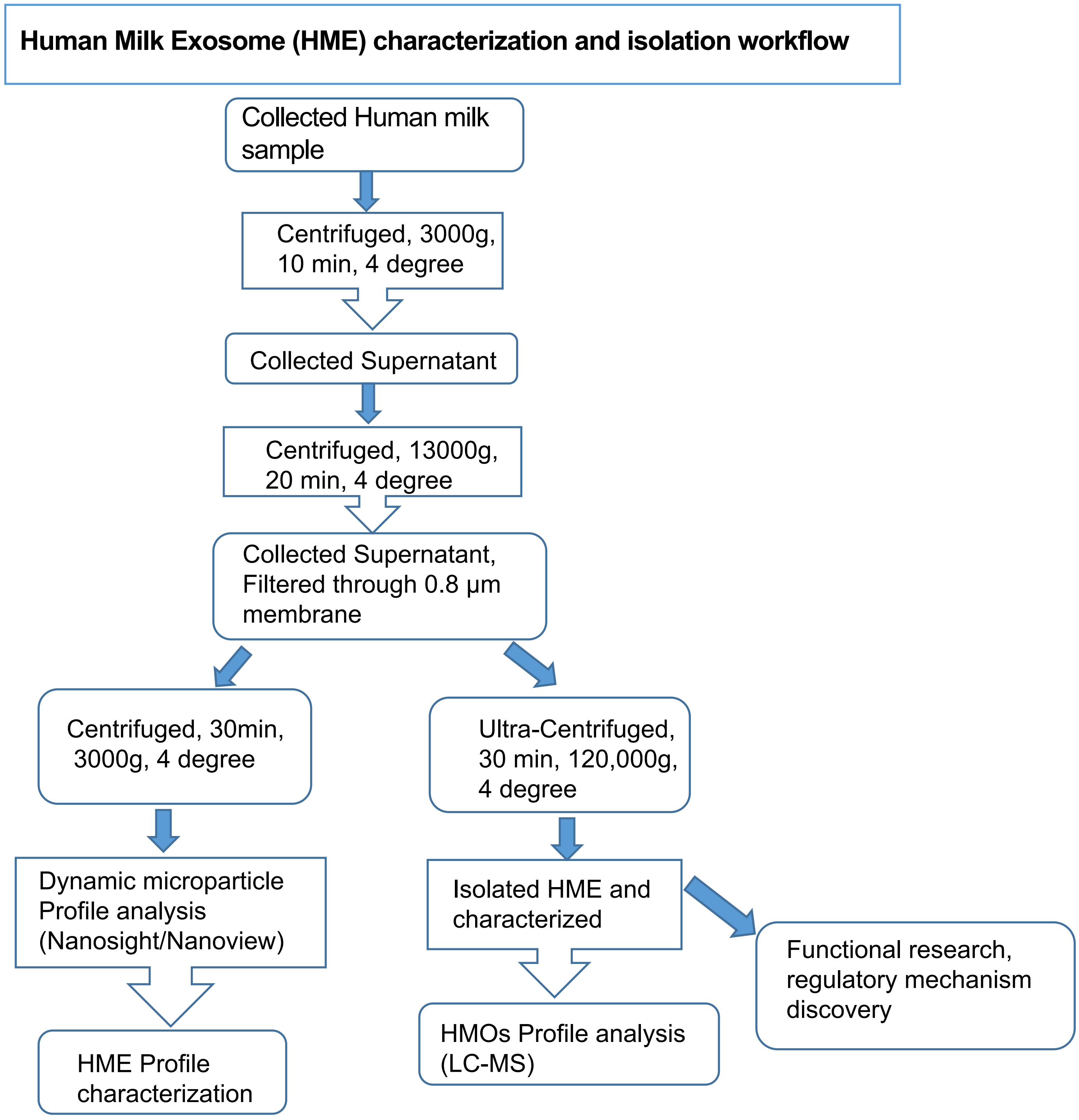
Human Milk Exosome (HME) characterization and isolation workflow
Collected Human milk sample
Centrifuged, 3000g, 10 min, 4 degree
Collected Supernatant
Centrifuged, 13000g, 20 min, 4 degree
Collected Supernatant, Filtered through 0.8 μm membrane
Centrifuged, 30min, 3000g, 4 degree
Ultra-Centrifuged, 30 min, 120,000g, 4 degree
Dynamic microparticle Profile analysis (Nanosight/Nanoview)
Isolated HME and characterized
Functional research, regulatory mechanism discovery
HME Profile characterization
HMOs Profile analysis (LC-MS)

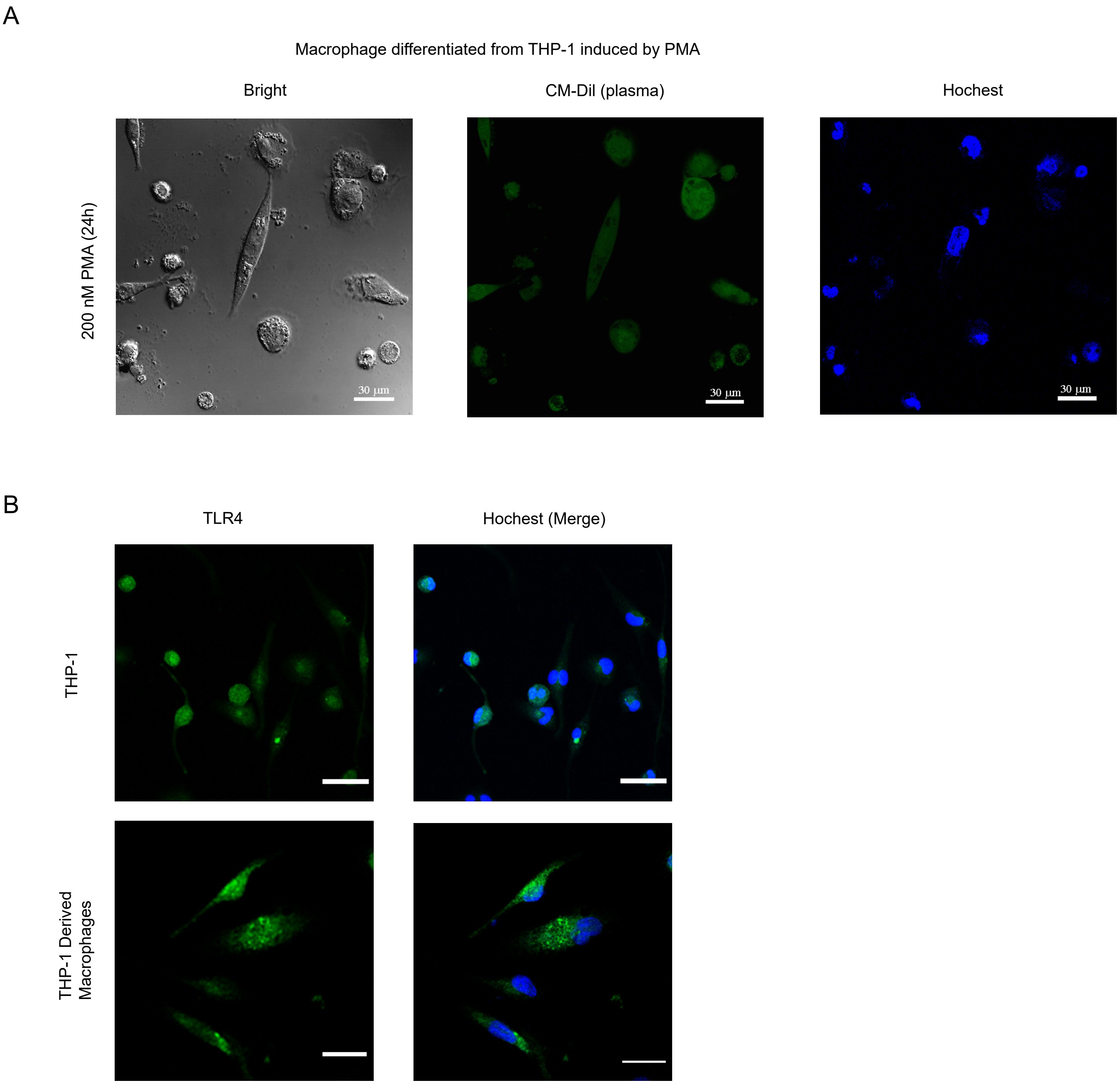
A
Macrophage differentiated from THP-1 induced by PMA
Bright
CM-Dil (plasma)
Hochest
200 nM PMA (24h)
30 μm
30 μm
30 μm
B
TLR4
Hochest (Merge)
THP-1
THP-1 Derived Macrophages

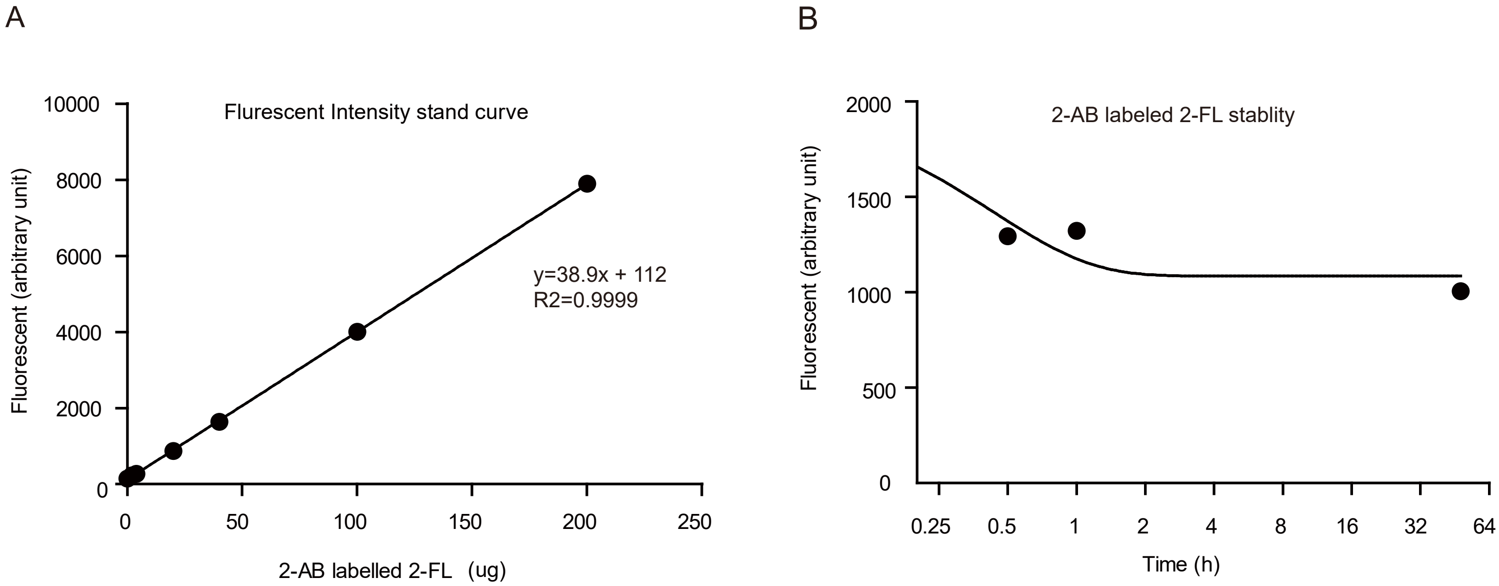
A
Flurescent Intensity stand curve
Fluorescent (arbitrary unit)
10000
8000
6000
4000
2000
0
0
50
100
150
200
250
2-AB labelled 2-FL (ug)
y=38.9x + 112
R2=0.9999
B
2-AB labeled 2-FL stablity
Fluorescent (arbitrary unit)
2000
1500
1000
500
0
0.25
0.5
1
2
4
8
16
32
64
Time (h)

Table S1. The significantly regulated genes by colostrum exosome capsulated oligosaccharides in macrophages.

Gene Symbol	Fold Change	CTRL Avg (log2)	HMEO Avg (log2)	P-val	FDR P-val	Description
A2M	-3.01	-1.31	0.28	5.24E-06	0.0002	alpha-2-macroglobulin
AAMP	-3.09	-1.28	0.35	0.0002	0.0007	angio-associated migratory cell protein
AASDHPPT	-3.12	-1.37	0.27	3.35E-05	0.0003	aminoadipate-semialdehyde dehydrogenase-phosphopantetheinyl transferase
ABCB8	-3.05	-1.36	0.25	2.67E-05	0.0003	ATP binding cassette subfamily B member 8
ABCC10	-3.12	-1.38	0.26	5.16E-05	0.0004	ATP binding cassette subfamily C member 10
ABCC6P2	3.03	0.2	-1.4	1.01E-05	0.0002	ATP binding cassette subfamily C member 6 pseudogene 2
ABCG5	-3.09	-1.28	0.35	7.65E-06	0.0002	ATP binding cassette subfamily G member 5
ABCG8	-3.13	-1.3	0.35	1.12E-05	0.0002	ATP binding cassette subfamily G member 8
ABHD17AP1	3.12	0.26	-1.38	2.87E-05	0.0003	abhydrolase domain containing 17A pseudogene 1
ABHD17AP1	3.12	0.26	-1.38	2.87E-05	0.0003	abhydrolase domain containing 17A pseudogene 1
ABHD17C	3.12	0.26	-1.38	5.70E-06	0.0002	abhydrolase domain containing 17C
ABO	-3.03	-1.31	0.29	9.58E-05	0.0005	ABO blood group (transferase A, alpha 1-3-N-acetylgalactosaminyltransferase; transferase B, alpha 1-3-galactosyltransferase)
ABRA	-3.06	-1.33	0.28	0.0001	0.0006	actin binding Rho activating protein
ABT1	3.48	0.34	-1.46	7.62E-06	0.0002	activator of basal transcription 1
ACAN	3.04	0.4	-1.2	5.49E-06	0.0002	aggrecan
ACER2	-3.14	-1.37	0.28	2.94E-05	0.0003	alkaline ceramidase 2
ACER3	3.65	0.43	-1.44	2.67E-05	0.0003	alkaline ceramidase 3
ACO2	-3.01	-1.3	0.29	7.18E-06	0.0002	aconitase 2, mitochondrial
ACOT13	3.37	0.32	-1.43	2.42E-06	0.0002	acyl-CoA thioesterase 13
ACP5	-3.07	-1.35	0.27	0.0001	0.0006	acid phosphatase 5, tartrate resistant
ACSF3	3.4	0.32	-1.44	4.76E-06	0.0002	acyl-CoA synthetase family member 3
ACSM2B; ACSM2A	3.24	0.32	-1.38	2.95E-05	0.0003	acyl-CoA synthetase medium-chain family member 2B; acyl-CoA synthetase medium-chain family member 2A
ACTL7A	3.31	0.33	-1.4	2.59E-05	0.0003	actin-like 7A
ACTL8	-3.29	-1.31	0.4	1.50E-05	0.0002	actin-like 8
ACTN1	-3.23	-1.36	0.34	9.43E-06	0.0002	actinin, alpha 1
ACTN3	-3.15	-1.38	0.27	4.17E-05	0.0003	actinin, alpha 3 (gene/pseudogene)
ACTR8	-3.03	-1.32	0.28	2.40E-05	0.0002	ARP8 actin-related protein 8 homolog (yeast)
ACVR1B	-3.01	-1.31	0.28	3.95E-05	0.0003	activin A receptor type IB
ADAM7	-3.04	-1.38	0.22	0.0001	0.0006	ADAM metallopeptidase domain 7
ADAM8	-3.09	-1.28	0.35	2.72E-05	0.0003	ADAM metallopeptidase domain 8
ADAMTS5	3.07	0.17	-1.44	1.14E-05	0.0002	ADAM metallopeptidase with thrombospondin type 1 motif 5
ADAT2	-3.29	-1.31	0.4	2.44E-05	0.0002	adenosine deaminase, tRNA-specific 2
ADCK2	3.01	0.21	-1.38	5.28E-05	0.0004	aarF domain containing kinase 2
ADCY10P1	3.51	0.34	-1.47	0.0003	0.0012	adenylate cyclase 10 (soluble) pseudogene 1

ADCY2	3.26	0.28	-1.43	9.17E-06	0.0002	adenylate cyclase 2 (brain)
ADCY5	3.23	0.4	-1.29	1.34E-05	0.0002	adenylate cyclase 5
ADD2	3.14	0.36	-1.29	1.12E-05	0.0002	adducin 2 (beta)
ADGRB2	3.05	0.23	-1.38	3.81E-05	0.0003	adhesion G protein-coupled receptor B2
ADGRG6	-3.05	-1.31	0.29	2.20E-05	0.0002	adhesion G protein-coupled receptor G6
ADGRL2	-3.05	-1.3	0.31	7.13E-06	0.0002	adhesion G protein-coupled receptor L2
ADH7	-3.05	-1.31	0.3	2.11E-05	0.0002	alcohol dehydrogenase 7 (class IV), mu or sigma polypeptide
ADIRF; AGAP11	-3.04	-1.28	0.32	9.50E-05	0.0005	adipogenesis regulatory factor; ankyrin repeat and GTPase domain Arf GTPase activating protein 11
ADM	3.29	0.41	-1.31	4.08E-05	0.0003	adrenomedullin
SPECC1L-ADORA2	-3.19	-1.31	0.36	2.77E-05	0.0003	adenosine A2a receptor; SPECC1L-ADORA2A readthrough (NMD candidate); sperm antigen with calponin homology and coiled-coil domains 1-like
ADPRHL1	3.07	0.22	-1.4	1.14E-05	0.0002	ADP-ribosylhydrolase like 1
ADRB2	-3.16	-1.38	0.28	4.14E-05	0.0003	adrenoceptor beta 2, surface
ADRBK1	-3.33	-1.33	0.4	0.0001	0.0006	adrenergic, beta, receptor kinase 1
ADRBK2	-3.2	-1.31	0.36	5.44E-05	0.0004	adrenergic, beta, receptor kinase 2
ADRM1	3.51	0.43	-1.38	2.93E-06	0.0002	adhesion regulating molecule 1
AFF2	3	0.06	-1.53	5.80E-05	0.0004	AF4/FMR2 family, member 2
AP5; AGAP9; AGAP	-3.03	-1.32	0.28	2.90E-05	0.0003	ArfGAP with GTPase domain, ankyrin repeat and PH domain 6; ArfGAP with GTPase domain, ankyrin repeat and PH domain 5; ArfGAP with GTPase domain, ankyrin repeat and PH domain 9; ankyrin repeat and GTPase domain Arf GTPase activating protein 11; ArfGAP with GTPase domain, ankyrin repeat and PH domain 7, pseudogene
AGAP6; AGAP9	-3.07	-1.36	0.25	1.20E-05	0.0002	ArfGAP with GTPase domain, ankyrin repeat and PH domain 6; ArfGAP with GTPase domain, ankyrin repeat and PH domain 9
AP6; AGAP9; AGAP	-3.01	-1.31	0.28	9.90E-06	0.0002	ArfGAP with GTPase domain, ankyrin repeat and PH domain 6; ArfGAP with GTPase domain, ankyrin repeat and PH domain 9; ankyrin repeat and GTPase domain Arf GTPase activating protein 11
GAP6; AGAP9; AGA	-3.03	-1.33	0.27	7.49E-06	0.0002	ArfGAP with GTPase domain, ankyrin repeat and PH domain 6; ArfGAP with GTPase domain, ankyrin repeat and PH domain 9; ArfGAP with GTPase domain, ankyrin repeat and PH domain 4
; AGAP9; AGAP4; A	-3.01	-1.32	0.27	9.46E-06	0.0002	ArfGAP with GTPase domain, ankyrin repeat and PH domain 6; ArfGAP with GTPase domain, ankyrin repeat and PH domain 9; ArfGAP with GTPase domain, ankyrin repeat and PH domain 4; ankyrin repeat and GTPase domain Arf GTPase activating protein 11
; AGAP9; AGAP4; A	-3.06	-1.33	0.28	2.83E-05	0.0003	ArfGAP with GTPase domain, ankyrin repeat and PH domain 6; ArfGAP with GTPase domain, ankyrin repeat and PH domain 9; ArfGAP with GTPase domain, ankyrin repeat and PH domain 4; ArfGAP with GTPase domain, ankyrin repeat and PH domain 7, pseudogene
AGAP9	-3.01	-1.31	0.28	1.21E-05	0.0002	ArfGAP with GTPase domain, ankyrin repeat and PH domain 9
AGAP9	-3.01	-1.31	0.28	1.21E-05	0.0002	ArfGAP with GTPase domain, ankyrin repeat and PH domain 9
AGBL2	-3.12	-1.28	0.36	3.70E-06	0.0002	ATP/GTP binding protein-like 2
AGXT	-3.2	-1.31	0.36	8.56E-06	0.0002	alanine-glyoxylate aminotransferase
AHDC1	3	0.16	-1.43	5.76E-06	0.0002	AT hook, DNA binding motif, containing 1
AIF1	3.25	0.5	-1.2	0.0002	0.0010	allograft inflammatory factor 1
AIF1	3.25	0.5	-1.2	0.0002	0.0010	allograft inflammatory factor 1
AIF1	3.25	0.5	-1.2	0.0002	0.0010	allograft inflammatory factor 1
AKIRIN1	-3.16	-1.38	0.28	1.67E-05	0.0002	akirin 1
AKR1C4	-3.14	-1.38	0.27	1.50E-05	0.0002	aldo-keto reductase family 1, member C4
AKR7A2	3.21	0.29	-1.39	4.12E-05	0.0003	aldo-keto reductase family 7, member A2
AKR7A2P1	3.46	0.39	-1.4	0.0002	0.0009	aldo-keto reductase family 7, member A2 pseudogene 1
ALDH1A2	3.06	0.32	-1.29	7.70E-06	0.0002	aldehyde dehydrogenase 1 family, member A2
ALDH5A1	3.02	0.39	-1.2	9.54E-06	0.0002	aldehyde dehydrogenase 5 family, member A1
ALDH8A1	-3.2	-1.42	0.26	2.33E-05	0.0002	aldehyde dehydrogenase 8 family, member A1
ALG10	-3.14	-1.36	0.29	3.60E-05	0.0003	ALG10, alpha-1,2-glucosyltransferase

ALG10B; ALG10	-3.07	-1.33	0.29	4.50E-05	0.0003	ALG10B, alpha-1,2-glucosyltransferase; ALG10, alpha-1,2-glucosyltransferase
ALKBH2	-3.12	-1.36	0.28	3.56E-05	0.0003	alkB homolog 2, alpha-ketoglutarate-dependent dioxygenase
LKBH3; SEC14L1P	3.1	0.43	-1.2	3.19E-05	0.0003	alkB homolog 3, alpha-ketoglutarate-dependent dioxygenase; SEC14-like 1 pseudogene 1
ALKBH5	3.26	0.32	-1.38	9.20E-06	0.0002	alkB homolog 5, RNA demethylase
ALS2CR12	-3.12	-1.36	0.28	1.67E-05	0.0002	amyotrophic lateral sclerosis 2 chromosome region candidate 12
AMER3	-3.12	-1.36	0.28	2.64E-05	0.0003	APC membrane recruitment protein 3
AMHR2	-3.02	-1.33	0.26	0.0001	0.0006	anti-Mullerian hormone receptor, type II
AMOTL1	3.26	0.32	-1.38	4.05E-06	0.0002	angiomotin like 1
AMPD3	-3.37	-1.43	0.32	3.84E-05	0.0003	adenosine monophosphate deaminase 3
AMT; NICN1	-3.08	-1.31	0.31	0.0001	0.0007	aminomethyltransferase; nicolin 1
AMZ1	3.2	0.25	-1.43	1.34E-05	0.0002	archaelysin family metallopeptidase 1
ANAPC10	-3.33	-1.39	0.35	4.36E-05	0.0003	anaphase promoting complex subunit 10
ANAPC13	3.26	0.32	-1.38	2.96E-05	0.0003	anaphase promoting complex subunit 13
ANGPT4	3.37	0.32	-1.43	3.11E-06	0.0002	angiopoietin 4
ANKRD13B	-3.23	-1.32	0.37	2.38E-05	0.0002	ankyrin repeat domain 13B
ANKRD2	-3.04	-1.28	0.32	2.87E-05	0.0003	ankyrin repeat domain 2 (stretch responsive muscle)
ANKRD29	-3	-1.28	0.31	4.17E-06	0.0002	ankyrin repeat domain 29
ANKRD30BL	-3.35	-1.41	0.33	0.0001	0.0007	ankyrin repeat domain 30B-like
ANKRD33	3.35	0.36	-1.39	4.07E-06	0.0002	ankyrin repeat domain 33
ANKRD53	-3.03	-1.3	0.3	2.80E-05	0.0003	ankyrin repeat domain 53
ANKRD6	3.47	0.32	-1.47	3.35E-06	0.0002	ankyrin repeat domain 6
ANO8	-3.01	-1.31	0.28	6.06E-05	0.0004	anoctamin 8
ANTXR2	3.26	0.32	-1.38	0.0001	0.0006	anthrax toxin receptor 2
ANXA11	-3.01	-1.31	0.28	3.35E-05	0.0003	annexin A11
ANXA2	-3.37	-1.44	0.31	1.16E-05	0.0002	annexin A2
ANXA9	3.3	0.29	-1.44	2.74E-06	0.0002	annexin A9
AP3S1	-3.11	-1.31	0.32	1.28E-05	0.0002	adaptor-related protein complex 3, sigma 1 subunit
AP5Z1	-3.2	-1.44	0.24	9.63E-05	0.0005	adaptor-related protein complex 5, zeta 1 subunit
APBB1	3.37	0.32	-1.43	7.67E-06	0.0002	amyloid beta (A4) precursor protein-binding, family B, member 1 (Fe65)
API5	3.97	0.51	-1.48	3.47E-06	0.0002	apoptosis inhibitor 5
APOBEC2	-3.16	-1.35	0.31	5.63E-05	0.0004	apolipoprotein B mRNA editing enzyme, catalytic polypeptide-like 2
OC2; APOC4-APO	3.26	0.32	-1.38	3.04E-05	0.0003	apolipoprotein C-II; APOC4-APOC2 readthrough (NMD candidate)
APOH	3.44	0.34	-1.44	6.46E-06	0.0002	apolipoprotein H (beta-2-glycoprotein I)
AQP1	-3.1	-1.38	0.25	1.98E-05	0.0002	aquaporin 1 (Colton blood group)
ARF1	3.42	0.39	-1.38	5.89E-06	0.0002	ADP-ribosylation factor 1
ARF4	-3.2	-1.31	0.36	4.32E-06	0.0002	ADP-ribosylation factor 4
ARFGAP2	3.06	0.21	-1.4	5.60E-05	0.0004	ADP-ribosylation factor GTPase activating protein 2
ARFRP1	-3.05	-1.37	0.24	3.77E-05	0.0003	ADP-ribosylation factor related protein 1

ARGFX	-3.18	-1.38	0.29	2.24E-05	0.0002	arginine-fifty homeobox
ARHGAP23	-3.15	-1.43	0.22	2.42E-05	0.0002	Rho GTPase activating protein 23
ARHGAP25	-3.07	-1.34	0.28	5.37E-06	0.0002	Rho GTPase activating protein 25
ARHGAP27	3.56	0.43	-1.4	3.89E-06	0.0002	Rho GTPase activating protein 27
ARHGEF15	-3.14	-1.37	0.28	4.44E-05	0.0003	Rho guanine nucleotide exchange factor 15
ARHGEF16	-3.06	-1.31	0.3	1.85E-05	0.0002	Rho guanine nucleotide exchange factor 16
RHGEF25; SLC26A1	-3.02	-1.35	0.24	3.34E-05	0.0003	Rho guanine nucleotide exchange factor 25; solute carrier family 26, member 10
RHGEF35; ARHGEF	3.19	0.28	-1.39	2.03E-05	0.0002	Rho guanine nucleotide exchange factor 35; Rho guanine nucleotide exchange factor 5
ARHGEF37	3.31	0.32	-1.4	1.85E-05	0.0002	Rho guanine nucleotide exchange factor 37
ARID3C	-3.16	-1.38	0.28	3.36E-05	0.0003	AT rich interactive domain 3C (BRIGHT-like)
ARL6IP1	-3.09	-1.28	0.35	5.70E-06	0.0002	ADP-ribosylation factor like GTPase 6 interacting protein 1
ARL6IP4	-3.2	-1.31	0.36	4.25E-05	0.0003	ADP-ribosylation factor like GTPase 6 interacting protein 4
ARL9	-3.06	-1.33	0.28	6.43E-05	0.0004	ADP-ribosylation factor like GTPase 9
ARMC1	-3.04	-1.38	0.22	1.32E-05	0.0002	armadillo repeat containing 1
ARMC4	-3.33	-1.32	0.41	5.68E-05	0.0004	armadillo repeat containing 4
ARPC2	-3.09	-1.28	0.35	8.30E-05	0.0005	actin related protein 2/3 complex subunit 2
ARPC3	3.61	0.47	-1.38	9.14E-05	0.0005	actin related protein 2/3 complex subunit 3
ARPC5	-3.15	-1.33	0.32	1.02E-05	0.0002	actin related protein 2/3 complex subunit 5
3MT; BORCS7-ASN	3.26	0.32	-1.38	2.08E-05	0.0002	arsenite methyltransferase; BORCS7-ASMT readthrough (NMD candidate)
ASAP1	-3.01	-1.38	0.21	2.97E-05	0.0003	ArfGAP with SH3 domain, ankyrin repeat and PH domain 1
ASAP3	-3.09	-1.28	0.35	4.08E-06	0.0002	ArfGAP with SH3 domain, ankyrin repeat and PH domain 3
ASB16	-3.32	-1.38	0.35	2.92E-05	0.0003	ankyrin repeat and SOCS box containing 16
ASB18	-3.04	-1.36	0.25	2.81E-05	0.0003	ankyrin repeat and SOCS box containing 18
ASB7	3.07	0.42	-1.2	1.74E-05	0.0002	ankyrin repeat and SOCS box containing 7
ASCC2	-3.32	-1.38	0.35	3.05E-06	0.0002	activating signal cointegrator 1 complex subunit 2
ASF1B	-3.11	-1.31	0.32	9.85E-06	0.0002	anti-silencing function 1B histone chaperone
ASTN1	3.02	0.39	-1.2	1.41E-05	0.0002	astrotactin 1
ATF5; MIR4751	3.07	0.24	-1.38	8.23E-06	0.0002	activating transcription factor 5; microRNA 4751
ATF6B; TNXB	3.48	0.42	-1.38	6.48E-06	0.0002	activating transcription factor 6 beta; tenascin XB
ATF6B; TNXB	3.39	0.37	-1.39	7.65E-06	0.0002	activating transcription factor 6 beta; tenascin XB
ATL2	3.43	0.35	-1.43	8.47E-06	0.0002	atlastin GTPase 2
ATP1B4	-3.15	-1.36	0.3	3.24E-05	0.0003	ATPase, Na+/K+ transporting, beta 4 polypeptide
ATP2A3	3.07	0.42	-1.2	4.17E-06	0.0002	ATPase, Ca++ transporting, ubiquitous
ATP5I	-3.12	-1.32	0.32	2.53E-05	0.0003	ATP synthase, H+ transporting, mitochondrial Fo complex subunit E
ATP5L2	3.02	0.39	-1.2	1.16E-05	0.0002	ATP synthase, H+ transporting, mitochondrial Fo complex subunit G2
ATP6V0A1	3.28	0.29	-1.43	0.0001	0.0007	ATPase, H+ transporting, lysosomal V0 subunit a1
ATP6V0E2-AS1	-3.23	-1.35	0.34	6.15E-05	0.0004	ATP6V0E2 antisense RNA 1
ATP6V1E2	3.3	0.32	-1.4	3.31E-06	0.0002	ATPase, H+ transporting, lysosomal 31kDa, V1 subunit E2

ATXN2L	3.45	0.39	-1.4	6.67E-06	0.0002	ataxin 2-like
ATXN3L	-3.01	-1.31	0.28	4.11E-05	0.0003	ataxin 3-like
ATXN7L1	3.04	0.22	-1.38	4.30E-06	0.0002	ataxin 7-like 1
ATXN7L3	-3.07	-1.28	0.34	3.14E-05	0.0003	ataxin 7-like 3
AUNIP	3.1	0.32	-1.31	1.03E-05	0.0002	aurora kinase A and ninein interacting protein
AURKB	-3.01	-1.31	0.28	3.46E-06	0.0002	aurora kinase B
AVPI1	3.02	0.39	-1.2	4.41E-06	0.0002	arginine vasopressin-induced 1
AZGP1	3.01	0.19	-1.4	5.28E-05	0.0004	alpha-2-glycoprotein 1, zinc-binding
AZIN2	-3.01	-1.31	0.28	2.09E-05	0.0002	antizyme inhibitor 2
B3GALT5-AS1	3.42	0.39	-1.38	2.77E-05	0.0003	B3GALT5 antisense RNA 1
B3GLCT	-3.16	-1.38	0.28	9.11E-05	0.0005	beta 3-glucosyltransferase
B3GNT9	3.3	0.32	-1.4	2.20E-05	0.0002	UDP-GlcNAc:betaGal beta-1,3-N-acetylglucosaminyltransferase 9
B4GALNT2	-3.08	-1.36	0.26	1.74E-05	0.0002	beta-1,4-N-acetyl-galactosaminyl transferase 2
B4GALT6	3.26	0.32	-1.38	0.0001	0.0007	UDP-Gal:betaGlcNAc beta 1,4- galactosyltransferase, polypeptide 6
BAAT	-3.16	-1.38	0.28	5.45E-05	0.0004	bile acid-CoA:amino acid N-acyltransferase
BABAM1	-3.13	-1.28	0.36	2.38E-05	0.0002	BRISC and BRCA1 A complex member 1
BAIAP3	3.16	0.28	-1.38	8.77E-06	0.0002	BAI1-associated protein 3
BARD1	3.41	0.37	-1.4	2.98E-06	0.0002	BRCA1 associated RING domain 1
BARHL1	3.21	0.3	-1.38	2.65E-05	0.0003	BarH-like homeobox 1
BARX1	-3.1	-1.36	0.27	1.72E-05	0.0002	BARX homeobox 1
BATF3	3.4	0.38	-1.38	0.0002	0.0009	basic leucine zipper transcription factor, ATF-like 3
BCAN	-3.11	-1.31	0.32	1.09E-05	0.0002	brevican
BCAT2	3.27	0.33	-1.38	2.65E-06	0.0002	branched chain amino-acid transaminase 2, mitochondrial
BCKDK	3.3	0.32	-1.4	7.63E-06	0.0002	branched chain ketoacid dehydrogenase kinase
BCL11A	-3.03	-1.32	0.28	2.75E-05	0.0003	B-cell CLL/lymphoma 11A (zinc finger protein)
BCL2L1	-3.05	-1.31	0.29	6.50E-05	0.0004	BCL2-like 1
BCLAF1	-3.33	-1.38	0.35	0.0002	0.0007	BCL2-associated transcription factor 1
BCRP2; BCR	3.46	0.39	-1.4	1.59E-05	0.0002	breakpoint cluster region pseudogene 2; breakpoint cluster region
BEGAIN	3.52	0.43	-1.38	7.14E-06	0.0002	brain-enriched guanylate kinase-associated
BEX4	-3.07	-1.3	0.32	7.24E-05	0.0004	brain expressed X-linked 4
BHLHB9	3.11	0.24	-1.4	5.51E-05	0.0004	basic helix-loop-helix domain containing, class B, 9
BIRC8	3.25	0.3	-1.4	3.39E-05	0.0003	baculoviral IAP repeat containing 8
BLOC1S1	3.06	0.32	-1.29	1.99E-05	0.0002	biogenesis of lysosomal organelles complex-1, subunit 1
BLVRB	-3.15	-1.38	0.27	2.34E-05	0.0002	biliverdin reductase B
BMP5	-3.01	-1.31	0.28	1.19E-05	0.0002	bone morphogenetic protein 5
BMP7	3.59	0.43	-1.41	2.34E-05	0.0002	bone morphogenetic protein 7
BOP1	-3.13	-1.35	0.3	1.00E-05	0.0002	block of proliferation 1
BRD2	-3.03	-1.32	0.28	8.59E-05	0.0005	bromodomain containing 2

BRD2	-3.12	-1.36	0.28	0.0001	0.0006	bromodomain containing 2
BRD7	-3.08	-1.28	0.34	1.17E-05	0.0002	bromodomain containing 7
BRD9	-3.39	-1.41	0.35	4.63E-05	0.0003	bromodomain containing 9
BRK1	-3.04	-1.36	0.24	5.03E-05	0.0003	BRICK1, SCAR/WAVE actin-nucleating complex subunit
BRS3	-3.05	-1.41	0.2	5.84E-06	0.0002	bombesin-like receptor 3
BTBD1	-3.11	-1.31	0.33	2.41E-05	0.0002	BTB (POZ) domain containing 1
BTG4	-3.14	-1.37	0.28	7.22E-05	0.0004	B-cell translocation gene 4
BTK	3.31	0.33	-1.4	7.92E-06	0.0002	Bruton agammaglobulinemia tyrosine kinase
BTRC	3.55	0.36	-1.47	2.34E-05	0.0002	beta-transducin repeat containing E3 ubiquitin protein ligase
C11orf31	3.52	0.43	-1.38	7.14E-06	0.0002	chromosome 11 open reading frame 31
C11orf40	-3.01	-1.28	0.31	3.57E-05	0.0003	chromosome 11 open reading frame 40
C11orf53	3.04	0.4	-1.2	2.77E-05	0.0003	chromosome 11 open reading frame 53
C11orf80; RCE1	-3.01	-1.31	0.28	5.26E-06	0.0002	chromosome 11 open reading frame 80; Ras converting CAAX endopeptidase 1
C11orf88	3.28	0.31	-1.4	2.14E-05	0.0002	chromosome 11 open reading frame 88
C12orf76	-3.02	-1.31	0.28	2.49E-05	0.0002	chromosome 12 open reading frame 76
C15orf39	3.05	0.31	-1.3	5.33E-06	0.0002	chromosome 15 open reading frame 39
C15orf43	-3	-1.33	0.25	2.11E-05	0.0002	chromosome 15 open reading frame 43
C15orf52	-3.04	-1.28	0.32	6.40E-06	0.0002	chromosome 15 open reading frame 52
C16orf13	-3.13	-1.31	0.34	3.08E-06	0.0002	chromosome 16 open reading frame 13
C16orf59	3.28	0.32	-1.4	2.12E-05	0.0002	chromosome 16 open reading frame 59
C16orf70	-3.03	-1.32	0.28	5.89E-06	0.0002	chromosome 16 open reading frame 70
C16orf71	-3.01	-1.31	0.28	4.44E-06	0.0002	chromosome 16 open reading frame 71
C18orf12	-3.16	-1.3	0.36	5.76E-06	0.0002	chromosome 18 open reading frame 12
C19orf60	-3.1	-1.36	0.27	1.24E-05	0.0002	chromosome 19 open reading frame 60
PRAMEF12	3.09	0.25	-1.38	4.05E-06	0.0002	chromosome 1 open reading frame 158; PRAME family member 12
C1orf186	3.41	0.32	-1.44	1.35E-05	0.0002	chromosome 1 open reading frame 186
C1orf216	3.33	0.53	-1.2	2.91E-05	0.0003	chromosome 1 open reading frame 216
C1orf74	-3.06	-1.39	0.23	1.21E-05	0.0002	chromosome 1 open reading frame 74
C1orf94	-3.1	-1.3	0.34	7.99E-06	0.0002	chromosome 1 open reading frame 94
C1RL-AS1	-3.06	-1.38	0.23	4.69E-05	0.0003	C1RL antisense RNA 1
C1S	-3.08	-1.31	0.31	3.59E-06	0.0002	complement component 1, s subcomponent
C2orf15	3.37	0.33	-1.43	8.74E-06	0.0002	chromosome 2 open reading frame 15
C2orf50	-3.09	-1.28	0.35	7.31E-06	0.0002	chromosome 2 open reading frame 50
C2orf70	-3.01	-1.31	0.28	4.80E-05	0.0003	chromosome 2 open reading frame 70
STK19	3.53	0.37	-1.44	2.29E-05	0.0002	complement component 4A (Rodgers blood group); complement component 4B (Chido blood group); complement component 4B (Chido blood group), copy 2; serine/threonine kinase 19
STK19	3.53	0.37	-1.44	2.29E-05	0.0002	complement component 4A (Rodgers blood group); complement component 4B (Chido blood group); complement component 4B (Chido blood group), copy 2; serine/threonine kinase 19
STK19	3.53	0.37	-1.44	2.29E-05	0.0002	complement component 4A (Rodgers blood group); complement component 4B (Chido blood group); complement component 4B (Chido blood group), copy 2; serine/threonine kinase 19
C4orf3	-3.09	-1.36	0.26	7.29E-06	0.0002	chromosome 4 open reading frame 3

C6orf1	-3.01	-1.31	0.28	1.29E-05	0.0002	chromosome 6 open reading frame 1
C6orf118	-3.23	-1.41	0.28	2.25E-05	0.0002	chromosome 6 open reading frame 118
C6orf203	3.58	0.41	-1.43	3.05E-06	0.0002	chromosome 6 open reading frame 203
C6orf222	3.54	0.44	-1.38	7.23E-05	0.0004	chromosome 6 open reading frame 222
C7	-3.01	-1.33	0.26	2.89E-05	0.0003	complement component 7
C7orf34	-3.14	-1.35	0.3	3.21E-05	0.0003	chromosome 7 open reading frame 34
C7orf55	3.31	0.34	-1.39	1.81E-05	0.0002	C7orf55-LUC7L2 readthrough; chromosome 7 open reading frame 55
TSC22D4	-3.15	-1.35	0.31	9.22E-06	0.0002	chromosome 7 open reading frame 61; TSC22 domain family, member 4
C7orf72	-3.09	-1.36	0.26	3.79E-05	0.0003	chromosome 7 open reading frame 72
C8orf74	3.12	0.25	-1.39	7.00E-06	0.0002	chromosome 8 open reading frame 74
S1PR3	-3.07	-1.28	0.34	2.69E-05	0.0003	chromosome 9 open reading frame 47; sphingosine-1-phosphate receptor 3
CA11	3.02	0.39	-1.2	5.65E-06	0.0002	carbonic anhydrase XI
CA6	-3.22	-1.41	0.28	3.16E-05	0.0003	carbonic anhydrase VI
CABP1	3.1	0.43	-1.2	7.73E-06	0.0002	calcium binding protein 1
CADM3	-3.12	-1.36	0.28	1.68E-05	0.0002	cell adhesion molecule 3
CADPS2	-3.22	-1.36	0.32	3.18E-05	0.0003	Ca++-dependent secretion activator 2
CALR	3.52	0.43	-1.38	1.58E-05	0.0002	calreticulin
CAND1	-3.06	-1.32	0.29	3.81E-06	0.0002	cullin-associated and neddylation-dissociated 1
CANX	-3.01	-1.31	0.28	1.03E-05	0.0002	calnexin
CAPN15	-3.11	-1.31	0.32	0.0002	0.0008	calpain 15
CAPN2	3.11	0.34	-1.29	1.14E-05	0.0002	calpain 2, (m/II) large subunit
CAPN5	3.1	0.43	-1.2	7.62E-05	0.0004	calpain 5
CAPNS1	-3.04	-1.38	0.22	1.04E-05	0.0002	calpain, small subunit 1
CAPRIN1	-3.12	-1.3	0.34	5.18E-06	0.0002	cell cycle associated protein 1
CAPZA1	3.47	0.32	-1.47	7.60E-06	0.0002	capping protein (actin filament) muscle Z-line, alpha 1
CAV1	3.3	0.31	-1.41	3.17E-05	0.0003	caveolin 1
CBARP	3.16	0.36	-1.3	3.95E-06	0.0002	calcium channel, voltage-dependent, beta subunit associated regulatory protein
CBS; CBSL	-3.02	-1.31	0.28	1.08E-05	0.0002	cystathionine-beta-synthase; cystathionine-beta-synthase like
CBY1	-3.01	-1.31	0.28	1.24E-05	0.0002	chibby homolog 1 (Drosophila)
CC2D1B	-3.01	-1.31	0.28	1.09E-05	0.0002	coiled-coil and C2 domain containing 1B
CCDC115	3.56	0.43	-1.4	2.77E-05	0.0003	coiled-coil domain containing 115
CCDC120	-3.16	-1.38	0.28	6.72E-06	0.0002	coiled-coil domain containing 120
CCDC129	3.06	0.23	-1.38	8.88E-06	0.0002	coiled-coil domain containing 129
CCDC134	-3.11	-1.31	0.32	2.53E-05	0.0003	coiled-coil domain containing 134
CCDC14	-3.21	-1.43	0.25	0.0001	0.0007	coiled-coil domain containing 14
CCDC17	-3	-1.28	0.31	8.99E-06	0.0002	coiled-coil domain containing 17
CCDC189	3.39	0.32	-1.44	1.18E-05	0.0002	coiled-coil domain containing 189
CCDC22	3.26	0.32	-1.38	1.47E-05	0.0002	coiled-coil domain containing 22

CCDC27	3.01	0.18	-1.41	9.98E-06	0.0002	coiled-coil domain containing 27
CCDC36	-3.1	-1.36	0.27	3.40E-05	0.0003	coiled-coil domain containing 36
CCDC58	3.37	0.32	-1.43	4.56E-05	0.0003	coiled-coil domain containing 58
CCDC61	3.02	0.39	-1.2	1.75E-05	0.0002	coiled-coil domain containing 61
CCDC63	-3.12	-1.36	0.28	8.94E-06	0.0002	coiled-coil domain containing 63
CCDC78	-3.05	-1.3	0.31	0.0002	0.0008	coiled-coil domain containing 78
CCER1	3.02	0.21	-1.38	2.78E-05	0.0003	coiled-coil glutamate rich protein 1
CCL1	-3.05	-1.35	0.26	4.84E-05	0.0003	chemokine (C-C motif) ligand 1
CCL2	3.3	0.32	-1.4	3.89E-06	0.0002	chemokine (C-C motif) ligand 2
CCL8	3.3	0.32	-1.4	7.95E-06	0.0002	chemokine (C-C motif) ligand 8
CCNA1	3.16	0.37	-1.29	2.88E-05	0.0003	cyclin A1
CCR5	-3.09	-1.35	0.28	1.41E-05	0.0002	chemokine (C-C motif) receptor 5 (gene/pseudogene)
CCR5	-3.09	-1.35	0.28	1.41E-05	0.0002	chemokine (C-C motif) receptor 5 (gene/pseudogene)
CCT8L2	-3.21	-1.36	0.32	9.37E-05	0.0005	chaperonin containing TCP1, subunit 8 (theta)-like 2
CD200R1L	3.42	0.33	-1.44	1.33E-05	0.0002	CD200 receptor 1 like
CD247	3.01	0.3	-1.29	8.72E-06	0.0002	CD247 molecule
CD320	-3.01	-1.31	0.28	4.19E-06	0.0002	CD320 molecule
CD74	3.02	0.39	-1.2	9.34E-06	0.0002	CD74 molecule, major histocompatibility complex, class II invariant chain
CD82	3.37	0.32	-1.43	1.68E-05	0.0002	CD82 molecule
CD84	-3.01	-1.28	0.31	5.75E-05	0.0004	CD84 molecule
CD8B	-3.21	-1.37	0.31	2.27E-05	0.0002	CD8b molecule
CD99L2	3.4	0.38	-1.38	8.21E-06	0.0002	CD99 molecule-like 2
CDC20	3.28	0.42	-1.29	3.76E-05	0.0003	cell division cycle 20
CDH7	3.42	0.39	-1.38	1.60E-05	0.0002	cadherin 7, type 2
CDK10	3.21	0.3	-1.38	6.24E-06	0.0002	cyclin-dependent kinase 10
CDK16	-3.37	-1.37	0.38	7.38E-06	0.0002	cyclin-dependent kinase 16
CDK18	-3.05	-1.28	0.33	1.03E-05	0.0002	cyclin-dependent kinase 18
CDK2AP2	3.3	0.32	-1.4	3.11E-06	0.0002	cyclin-dependent kinase 2 associated protein 2
CDK4	3.22	0.37	-1.31	5.51E-05	0.0004	cyclin-dependent kinase 4
CDK7	-3.01	-1.28	0.31	4.44E-06	0.0002	cyclin-dependent kinase 7
CDK7	-3.01	-1.28	0.31	4.44E-06	0.0002	cyclin-dependent kinase 7
CDKN1A	-3.01	-1.32	0.27	0.0004	0.0016	cyclin-dependent kinase inhibitor 1A (p21, Cip1)
CDS2	3.16	0.28	-1.38	0.0001	0.0007	CDP-diacylglycerol synthase 2
CDSN	-3.06	-1.34	0.27	7.86E-06	0.0002	corneodesmosin
CDV3	3.02	0.39	-1.2	6.20E-05	0.0004	CDV3 homolog (mouse)
CDY2A	3.42	0.39	-1.38	2.96E-06	0.0002	chromodomain protein, Y-linked, 2A; chromodomain protein, Y-linked, 2B
CDY2B	3.42	0.39	-1.38	4.40E-06	0.0002	chromodomain protein, Y-linked, 2A; chromodomain protein, Y-linked, 2B
CEACAM3	3.49	0.38	-1.43	6.42E-06	0.0002	carcinoembryonic antigen-related cell adhesion molecule 3

CEACAM4	-3.01	-1.31	0.28	2.69E-05	0.0003	carcinoembryonic antigen-related cell adhesion molecule 4
CEACAM8	-3.13	-1.3	0.35	0.0001	0.0007	carcinoembryonic antigen-related cell adhesion molecule 8
CEBPD	-3.01	-1.28	0.31	1.02E-05	0.0002	CCAAT/enhancer binding protein (C/EBP), delta
CELF1	-3.1	-1.31	0.32	7.21E-06	0.0002	CUGBP, Elav-like family member 1
CELP; CEL	-3.13	-1.38	0.26	5.74E-05	0.0004	carboxyl ester lipase pseudogene; carboxyl ester lipase
CEMP1	-3.17	-1.4	0.27	5.47E-05	0.0004	cementum protein 1
CEP164	-3.01	-1.31	0.28	2.97E-05	0.0003	centrosomal protein 164kDa
CEP170B	-3.16	-1.38	0.28	8.00E-06	0.0002	centrosomal protein 170B
CER1	3.1	0.43	-1.2	1.32E-05	0.0002	cerberus 1, DAN family BMP antagonist
CERCAM	3.19	0.47	-1.2	0.0002	0.0007	cerebral endothelial cell adhesion molecule
CERS5	3.4	0.38	-1.38	1.68E-05	0.0002	ceramide synthase 5
CES1P1; CES1	-3.06	-1.31	0.3	8.76E-05	0.0005	carboxylesterase 1 pseudogene 1; carboxylesterase 1
CES5A	-3.06	-1.33	0.28	1.30E-05	0.0002	carboxylesterase 5A
CETP	-3.22	-1.36	0.32	2.39E-05	0.0002	cholesteryl ester transfer protein, plasma
CFAP100	-3.01	-1.31	0.28	3.38E-05	0.0003	cilia and flagella associated protein 100
CFAP45	-3.15	-1.33	0.32	9.40E-06	0.0002	cilia and flagella associated protein 45
CFAP61	-3.01	-1.31	0.28	4.11E-05	0.0003	cilia and flagella associated protein 61
CFAP77	3.4	0.35	-1.41	2.59E-05	0.0003	cilia and flagella associated protein 77
CFAP97	3	0.25	-1.34	1.46E-05	0.0002	cilia and flagella associated protein 97
CFHR1	-3.36	-1.37	0.38	3.74E-05	0.0003	complement factor H-related 1
CFHR3	-3.14	-1.37	0.28	2.58E-05	0.0003	complement factor H-related 3
CFHR4	3.62	0.43	-1.43	0.0002	0.0010	complement factor H-related 4
CGB2	-3.01	-1.28	0.31	0.0003	0.0011	chorionic gonadotropin, beta polypeptide 2
CGB5	-3.04	-1.31	0.29	0.0002	0.0008	chorionic gonadotropin, beta polypeptide 5; chorionic gonadotropin, beta polypeptide 1; chorionic gonadotropin, beta polypeptide 2
CGB7	-3.02	-1.28	0.32	6.53E-05	0.0004	chorionic gonadotropin, beta polypeptide 5; chorionic gonadotropin, beta polypeptide 7; chorionic gonadotropin, beta polypeptide 2; neurotrophin 4; chorionic gonadotropin, beta polypeptide
CGRRF1	3.23	0.29	-1.4	9.22E-06	0.0002	cell growth regulator with ring finger domain 1
MORC3	-3.15	-1.38	0.27	2.33E-05	0.0002	chromatin assembly factor 1, subunit B (p60); MORC family CW-type zinc finger 3
CHGB	3.47	0.32	-1.47	6.56E-06	0.0002	chromogranin B
CHORDC1	3.51	0.32	-1.49	1.48E-05	0.0002	cysteine and histidine rich domain containing 1
CHPF	-3.19	-1.37	0.3	3.13E-05	0.0003	chondroitin polymerizing factor
CHRM3	3.55	0.39	-1.44	7.11E-06	0.0002	cholinergic receptor, muscarinic 3
CHST12	-3.06	-1.28	0.34	0.0007	0.0026	carbohydrate (chondroitin 4) sulfotransferase 12
CHST5	-3.55	-1.36	0.47	0.0003	0.0013	carbohydrate (N-acetylglucosamine 6-O) sulfotransferase 5
CHST6	-3.41	-1.36	0.4	3.73E-05	0.0003	carbohydrate (N-acetylglucosamine 6-O) sulfotransferase 6
CHST8	3.01	0.3	-1.29	1.19E-05	0.0002	carbohydrate (N-acetylgalactosamine 4-0) sulfotransferase 8
CHTF18	-3.13	-1.31	0.33	4.64E-06	0.0002	chromosome transmission fidelity factor 18
CKAP2L	3.08	0.32	-1.3	4.93E-06	0.0002	cytoskeleton associated protein 2-like
CKS1B	-3.09	-1.28	0.35	1.73E-05	0.0002	CDC28 protein kinase regulatory subunit 1B

CLDN15	-3.15	-1.34	0.32	1.77E-05	0.0002	claudin 15
CLEC18A	-3.01	-1.31	0.28	1.19E-05	0.0002	C-type lectin domain family 18, member A; C-type lectin domain family 18, member B; C-type lectin domain family 18, member C
CLEC2B	3.52	0.36	-1.46	8.26E-06	0.0002	C-type lectin domain family 2, member B
EXOSC7	3.04	0.19	-1.41	4.28E-06	0.0002	C-type lectin domain family 3, member B; exosome component 7
CLECL1	-3.01	-1.31	0.28	2.69E-05	0.0003	C-type lectin-like 1
CLIP2	3.06	0.41	-1.2	1.67E-05	0.0002	CAP-GLY domain containing linker protein 2
CLIP4	-3.2	-1.43	0.25	6.41E-05	0.0004	CAP-GLY domain containing linker protein family, member 4
PPIL3	-3.17	-1.36	0.3	2.18E-05	0.0002	CDC like kinase 1; peptidylprolyl isomerase (cyclophilin)-like 3
CLLU1	-3.07	-1.38	0.23	2.81E-05	0.0003	chronic lymphocytic leukemia up-regulated 1
CLN8	-3.16	-1.4	0.26	0.0001	0.0007	ceroid-lipofuscinosis, neuronal 8
CLPTM1	3.12	0.44	-1.2	0.0001	0.0006	cleft lip and palate associated transmembrane protein 1
CLRN2	-3.08	-1.28	0.34	8.76E-06	0.0002	clarin 2
CLU	-3.23	-1.38	0.31	5.37E-05	0.0004	clusterin
CLYBL	-3.06	-1.38	0.23	9.86E-05	0.0005	citrate lyase beta like
CMA1	-3.11	-1.31	0.32	3.12E-06	0.0002	chymase 1, mast cell
CNTNAP5	-3.22	-1.35	0.34	1.17E-05	0.0002	contactin associated protein-like 5
COL13A1	-3.06	-1.33	0.28	1.31E-05	0.0002	collagen, type XIII, alpha 1
COL18A1	-3.03	-1.28	0.32	4.12E-05	0.0003	collagen, type XVIII, alpha 1
COL21A1	-3.01	-1.31	0.28	1.28E-05	0.0002	collagen, type XXI, alpha 1
COL6A2	3.22	0.4	-1.29	1.10E-05	0.0002	collagen, type VI, alpha 2
COLEC11	3.02	0.39	-1.2	6.44E-05	0.0004	collectin subfamily member 11
COPA	3.04	0.2	-1.4	2.62E-05	0.0003	coatomer protein complex subunit alpha
COQ7	-3.12	-1.36	0.28	7.02E-06	0.0002	coenzyme Q7 homolog, ubiquinone (yeast)
CORO1A	-3.15	-1.39	0.27	4.24E-05	0.0003	coronin, actin binding protein, 1A
CORO2A	-3.12	-1.36	0.28	8.37E-06	0.0002	coronin, actin binding protein, 2A
CORO2B	3.06	0.32	-1.29	0.0001	0.0006	coronin, actin binding protein, 2B
CORO6	-3.16	-1.3	0.37	4.58E-06	0.0002	coronin 6
COX11	-3.03	-1.33	0.27	1.96E-05	0.0002	COX11 cytochrome c oxidase copper chaperone
COX7B2	-3.33	-1.41	0.32	4.64E-05	0.0003	cytochrome c oxidase subunit VIIb2
CPA2	-3.36	-1.38	0.36	1.39E-05	0.0002	carboxypeptidase A2 (pancreatic)
CPEB1	-3.01	-1.34	0.25	1.79E-05	0.0002	cytoplasmic polyadenylation element binding protein 1
CPLX1	-3.01	-1.31	0.28	1.19E-05	0.0002	complexin 1
CPLX3	-3.11	-1.31	0.32	1.07E-05	0.0002	complexin 3; lectin, mannose-binding, 1 like
CPO	-3.17	-1.38	0.28	4.36E-05	0.0003	carboxypeptidase O
CPSF3L	-3.33	-1.41	0.32	7.10E-05	0.0004	cleavage and polyadenylation specific factor 3-like
CPSF7	-3.11	-1.32	0.32	6.94E-05	0.0004	cleavage and polyadenylation specific factor 7
CR2	3.42	0.39	-1.38	3.28E-05	0.0003	complement component (3d/Epstein Barr virus) receptor 2
CREBRF	-3.15	-1.3	0.36	2.78E-05	0.0003	CREB3 regulatory factor

CRELD1	3.08	0.32	-1.3	1.10E-05	0.0002	cysteine rich with EGF-like domains 1
CRLF1	-3.2	-1.33	0.35	0.0001	0.0006	cytokine receptor-like factor 1
CRLF2	3.3	0.32	-1.4	1.09E-05	0.0002	cytokine receptor-like factor 2
CRMP1	-3.06	-1.31	0.3	1.16E-05	0.0002	collapsin response mediator protein 1
CROCCP3	-3.01	-1.31	0.28	7.70E-05	0.0005	ciliary rootlet coiled-coil, rootletin pseudogene 3
CRTC3	-3.14	-1.38	0.27	2.27E-05	0.0002	CREB regulated transcription coactivator 3
CRY1	3.03	0.16	-1.44	1.41E-05	0.0002	cryptochrome circadian clock 1
CRYAA	-3.01	-1.32	0.27	2.57E-05	0.0003	crystallin alpha A
CRYAB	3.01	0.39	-1.2	1.35E-05	0.0002	crystallin alpha B
CRYBB2	-3.02	-1.31	0.28	2.95E-05	0.0003	crystallin beta B2
CSAG1	-3.16	-1.36	0.3	5.44E-06	0.0002	chondrosarcoma associated gene 1
CSF2RB	-3.13	-1.28	0.36	3.48E-05	0.0003	colony stimulating factor 2 receptor, beta, low-affinity (granulocyte-macrophage)
CSNK1A1P1	3.37	0.32	-1.43	4.28E-06	0.0002	casein kinase 1, alpha 1 pseudogene 1
CSNK1E	-3.06	-1.33	0.28	3.81E-05	0.0003	casein kinase 1, epsilon
CSNK1G2	3.41	0.39	-1.38	8.84E-06	0.0002	casein kinase 1, gamma 2
CSNK2B	-3.04	-1.31	0.29	2.88E-05	0.0003	casein kinase 2, beta polypeptide; lymphocyte antigen 6 complex, locus G5B
LY6G5B	-3.04	-1.31	0.29	2.88E-05	0.0003	casein kinase 2, beta polypeptide; lymphocyte antigen 6 complex, locus G5B
CSNK2B	-3.08	-1.32	0.3	3.12E-05	0.0003	casein kinase 2, beta polypeptide; lymphocyte antigen 6 complex, locus G5B
CSPG4P5	3.67	0.43	-1.44	6.33E-05	0.0004	chondroitin sulfate proteoglycan 4 pseudogene 5
CSPG5	3.35	0.45	-1.29	4.74E-05	0.0003	chondroitin sulfate proteoglycan 5 (neuroglycan C)
CST5	3.44	0.34	-1.44	4.25E-05	0.0003	cystatin D
CSTB	3.36	0.28	-1.47	1.41E-05	0.0002	cystatin B (stefin B)
CTAGE10P	-3.22	-1.35	0.34	3.84E-05	0.0003	CTAGE family, member 10, pseudogene
CTAGE7P	3.74	0.43	-1.47	0.0001	0.0007	CTAGE family, member 7, pseudogene
CTGF	3.3	0.52	-1.2	9.56E-06	0.0002	connective tissue growth factor
CTRB1	3.25	0.32	-1.38	8.77E-06	0.0002	chymotrypsinogen B1
CTRB2	3.17	0.29	-1.38	7.40E-06	0.0002	chymotrypsinogen B2; chymotrypsinogen B1
CTSE	-3.01	-1.31	0.28	1.36E-05	0.0002	cathepsin E
CTSH	3.51	0.43	-1.38	3.07E-05	0.0003	cathepsin H
CTSZ	3.55	0.42	-1.4	2.01E-05	0.0002	cathepsin Z
CTXN1	-3.45	-1.38	0.4	0.0004	0.0017	cortexin 1
CUL7	3.2	0.39	-1.29	2.98E-05	0.0003	cullin 7
CWH43	-3.12	-1.36	0.28	1.21E-05	0.0002	cell wall biogenesis 43 C-terminal homolog
CXCL17	3.18	0.38	-1.29	5.40E-06	0.0002	chemokine (C-X-C motif) ligand 17
CXCL2	-3.03	-1.31	0.29	1.17E-05	0.0002	chemokine (C-X-C motif) ligand 2
CXorf56	-3.07	-1.35	0.27	9.61E-05	0.0005	chromosome X open reading frame 56
CYB5B	-3.23	-1.35	0.34	1.55E-05	0.0002	cytochrome b5 type B (outer mitochondrial membrane)
CYBA	-3.14	-1.33	0.32	3.28E-05	0.0003	cytochrome b-245, alpha polypeptide

CYP11A1	3.49	0.41	-1.39	2.31E-06	0.0002	cytochrome P450, family 11, subfamily A, polypeptide 1
CYP2B7P	3.5	0.36	-1.45	2.76E-06	0.0002	cytochrome P450, family 2, subfamily B, polypeptide 7, pseudogene
CYP4F11	-3	-1.32	0.26	0.0001	0.0006	cytochrome P450, family 4, subfamily F, polypeptide 11
CYP4F29P	3.44	0.36	-1.43	4.33E-05	0.0003	cytochrome P450, family 4, subfamily F, polypeptide 29, pseudogene
CYP4Z2P	3.35	0.36	-1.38	1.48E-05	0.0002	cytochrome P450, family 4, subfamily Z, polypeptide 2, pseudogene
CYP7B1	3.11	0.21	-1.43	5.17E-05	0.0004	cytochrome P450, family 7, subfamily B, polypeptide 1
CYTL1	3	0.18	-1.4	3.46E-05	0.0003	cytokine like 1
DAAM1	-3.22	-1.31	0.38	2.50E-05	0.0002	dishevelled associated activator of morphogenesis 1
DACT2	3.51	0.4	-1.41	1.19E-05	0.0002	dishevelled-binding antagonist of beta-catenin 2
DAO	3.3	0.32	-1.4	1.49E-05	0.0002	D-amino-acid oxidase
DAW1	-3.14	-1.3	0.36	3.42E-05	0.0003	dynein assembly factor with WDR repeat domains 1
DBF4B	3.04	0.16	-1.44	5.09E-06	0.0002	DBF4 zinc finger B
DBH	3.1	0.23	-1.4	1.03E-05	0.0002	dopamine beta-hydroxylase (dopamine beta-monooxygenase)
DBN1	-3.01	-1.31	0.28	1.45E-05	0.0002	drebrin 1
DBX2	-3.09	-1.38	0.24	4.99E-05	0.0003	developing brain homeobox 2
DCAF8	3.1	0.25	-1.38	5.83E-06	0.0002	DDB1 and CUL4 associated factor 8
DCD	-3.04	-1.38	0.22	0.0002	0.0007	dermcidin
DCDC1	-3.04	-1.28	0.32	1.06E-05	0.0002	doublecortin domain containing 1
DCTN4	-3.01	-1.31	0.28	6.21E-06	0.0002	dynactin 4 (p62)
ERI2	-3.15	-1.38	0.27	1.26E-05	0.0002	DCN1, defective in cullin neddylation 1, domain containing 3; ERI1 exoribonuclease family member 2
DCUN1D4	3.26	0.32	-1.38	1.13E-05	0.0002	DCN1, defective in cullin neddylation 1, domain containing 4
DCXR	-3.06	-1.33	0.28	6.30E-05	0.0004	dicarbonyl/L-xylulose reductase
DDB2	-3.1	-1.31	0.32	0.0001	0.0007	damage-specific DNA binding protein 2
DDC-AS1	-3.04	-1.31	0.29	1.31E-05	0.0002	DDC antisense RNA 1
DDI1	-3.06	-1.33	0.28	3.76E-05	0.0003	DNA-damage inducible 1 homolog 1
DDX51	-3.01	-1.31	0.28	7.91E-06	0.0002	DEAD (Asp-Glu-Ala-Asp) box polypeptide 51
DEFB123	-3.31	-1.28	0.45	0.0002	0.0010	defensin, beta 123
DEFB128	-3.08	-1.36	0.26	3.80E-05	0.0003	defensin, beta 128
DEFB4A	-3.12	-1.36	0.28	0.0002	0.0007	defensin, beta 4A; defensin, beta 4B
DEFB4A; DEFB4B	-3.12	-1.36	0.28	0.0002	0.0007	defensin, beta 4A; defensin, beta 4B
DENND1C	-3.01	-1.31	0.28	8.41E-05	0.0005	DENN/MADD domain containing 1C
DESI2	-3.06	-1.31	0.3	6.22E-06	0.0002	desumoylating isopeptidase 2
DGAT2L6	-3.38	-1.41	0.34	1.08E-05	0.0002	diacylglycerol O-acyltransferase 2-like 6
DGCR6	3.63	0.43	-1.43	2.34E-06	0.0002	DiGeorge syndrome critical region gene 6
DGKA	-3.16	-1.38	0.28	1.01E-05	0.0002	diacylglycerol kinase alpha
DGUOK	3.41	0.32	-1.44	4.58E-05	0.0003	deoxyguanosine kinase
DHRS2	3.52	0.43	-1.38	0.0003	0.0014	dehydrogenase/reductase (SDR family) member 2
DHRS3	-3.13	-1.3	0.35	9.90E-06	0.0002	dehydrogenase/reductase (SDR family) member 3

DHTKD1	3.49	0.42	-1.38	9.99E-06	0.0002	dehydrogenase E1 and transketolase domain containing 1
DHX16	-3.04	-1.28	0.32	9.13E-06	0.0002	DEAH (Asp-Glu-Ala-His) box polypeptide 16
DHX29	-3.03	-1.28	0.32	1.07E-05	0.0002	DEAH (Asp-Glu-Ala-His) box polypeptide 29
DHX58	3.03	0.4	-1.2	4.05E-06	0.0002	DEXH (Asp-Glu-X-His) box polypeptide 58
DIP2A	3.04	0.31	-1.29	7.66E-06	0.0002	disco-interacting protein 2 homolog A
DIRAS1	-3.16	-1.28	0.38	3.29E-06	0.0002	DIRAS family, GTP-binding RAS-like 1
DIRC2	3.17	0.22	-1.44	4.02E-05	0.0003	disrupted in renal carcinoma 2
DISP2	3.3	0.3	-1.43	4.57E-05	0.0003	dispatched homolog 2 (Drosophila)
DKC1; SNORA56	-3.12	-1.36	0.28	8.37E-06	0.0002	dyskeratosis congenita 1, dyskerin; small nucleolar RNA, H/ACA box 56
DKKL1	3.55	0.39	-1.44	3.07E-06	0.0002	dickkopf-like 1
DLC1	-3.01	-1.31	0.28	1.61E-05	0.0002	DLC1 Rho GTPase activating protein
DLEU2	-3.13	-1.32	0.32	6.42E-06	0.0002	deleted in lymphocytic leukemia 2 (non-protein coding)
DLEU7	-3.13	-1.28	0.36	1.29E-05	0.0002	deleted in lymphocytic leukemia, 7
DLGAP3	-3.03	-1.32	0.28	1.59E-05	0.0002	discs, large (Drosophila) homolog-associated protein 3
DLK1	3.03	0.29	-1.31	4.98E-05	0.0003	delta-like 1 homolog (Drosophila)
DLL1	-3.01	-1.35	0.24	1.19E-05	0.0002	delta-like 1 (Drosophila)
DLX5	3.5	0.43	-1.38	3.79E-05	0.0003	distal-less homeobox 5
DNAH12	3.26	0.28	-1.43	4.95E-06	0.0002	dynein, axonemal, heavy chain 12
DNAI2	-3.19	-1.38	0.29	4.15E-05	0.0003	dynein, axonemal, intermediate chain 2
DNAJA1	3.52	0.43	-1.38	2.57E-06	0.0002	DnaJ (Hsp40) homolog, subfamily A, member 1
DNAJB13	3.26	0.3	-1.4	4.42E-06	0.0002	DnaJ (Hsp40) homolog, subfamily B, member 13
DNAJC19	-3.07	-1.36	0.26	0.0007	0.0027	DnaJ (Hsp40) homolog, subfamily C, member 19
DNAJC30	3.42	0.39	-1.38	1.98E-05	0.0002	DnaJ (Hsp40) homolog, subfamily C, member 30
DNLZ; CARD9	3.1	0.43	-1.2	4.02E-06	0.0002	DNL-type zinc finger; caspase recruitment domain family, member 9
DNM1	3.3	0.32	-1.4	3.89E-06	0.0002	dynamin 1
DNTT	-3.31	-1.36	0.36	3.80E-05	0.0003	DNA nucleotidylexotransferase
DOK1	3.02	0.39	-1.2	9.34E-06	0.0002	docking protein 1
DPH7	-3.05	-1.4	0.21	1.90E-05	0.0002	diphthamide biosynthesis 7
DPM2	3.56	0.43	-1.4	7.61E-06	0.0002	dolichyl-phosphate mannosyltransferase polypeptide 2, regulatory subunit
DPP3	-3.14	-1.37	0.28	3.59E-06	0.0002	dipeptidyl-peptidase 3
DPP7	-3.22	-1.36	0.32	4.23E-06	0.0002	dipeptidyl-peptidase 7
DPY19L1	-3.07	-1.36	0.26	9.63E-06	0.0002	dpy-19-like 1 (C. elegans)
DPYSL2	3.03	0.15	-1.44	1.01E-05	0.0002	dihydropyrimidinase-like 2
DRAM2	3.15	0.21	-1.44	3.61E-06	0.0002	DNA-damage regulated autophagy modulator 2
DRD5	3.02	0.39	-1.2	5.22E-06	0.0002	dopamine receptor D5
DRD5P2	-3.12	-1.36	0.28	2.52E-05	0.0003	dopamine receptor D5 pseudogene 2
DRGX	-3.09	-1.35	0.28	0.0002	0.0009	dorsal root ganglia homeobox
DSEL	-3.07	-1.3	0.32	2.88E-05	0.0003	dermatan sulfate epimerase-like

DSG3	3.34	0.34	-1.4	7.22E-06	0.0002	desmoglein 3
DTD2	3.75	0.46	-1.44	5.03E-06	0.0002	D-tyrosyl-tRNA deacylase 2 (putative)
DUOXA1	-3.11	-1.28	0.36	1.15E-05	0.0002	dual oxidase maturation factor 1
DUOXA2	3.1	0.43	-1.2	4.42E-05	0.0003	dual oxidase maturation factor 2
DUSP5	3.26	0.32	-1.38	9.64E-05	0.0005	dual specificity phosphatase 5
DYDC2	-3.23	-1.38	0.31	8.42E-05	0.0005	DPY30 domain containing 2
DYNC1I1	-3.04	-1.4	0.21	7.39E-06	0.0002	dynein, cytoplasmic 1, intermediate chain 1
E2F2	-3.03	-1.31	0.29	6.18E-05	0.0004	E2F transcription factor 2
EAPP	-3.04	-1.38	0.22	2.90E-05	0.0003	E2F-associated phosphoprotein
EARS2	3.3	0.32	-1.4	6.54E-06	0.0002	glutamyl-tRNA synthetase 2, mitochondrial
EBI3	3.13	0.45	-1.2	1.07E-05	0.0002	Epstein-Barr virus induced 3
ECM1	3.49	0.38	-1.43	6.69E-06	0.0002	extracellular matrix protein 1
EDAR	-3.01	-1.31	0.28	7.03E-05	0.0004	ectodysplasin A receptor
EFEMP2	3.15	0.46	-1.2	4.47E-06	0.0002	EGF containing fibulin-like extracellular matrix protein 2
EGLN1	3.16	0.28	-1.38	5.49E-06	0.0002	egl-9 family hypoxia-inducible factor 1
EGR3	-3.15	-1.37	0.28	4.68E-06	0.0002	early growth response 3
EHD3	3.19	0.47	-1.2	0.0002	0.0009	EH domain containing 3
EHMT1-IT1	-3.01	-1.31	0.28	3.95E-05	0.0003	EHMT1 intronic transcript 1
EIF2B2	-3.18	-1.38	0.29	2.49E-05	0.0002	eukaryotic translation initiation factor 2B, subunit 2 beta, 39kDa
EIF2S2	-3.37	-1.36	0.39	0.0002	0.0010	eukaryotic translation initiation factor 2, subunit 2 beta, 38kDa
EIF3J	-3.12	-1.36	0.28	2.39E-05	0.0002	eukaryotic translation initiation factor 3, subunit J
EIF3L	-3.06	-1.33	0.28	0.0002	0.0011	eukaryotic translation initiation factor 3, subunit L
EIF4E1B	-3.2	-1.31	0.36	0.0003	0.0011	eukaryotic translation initiation factor 4E family member 1B
EIF4G3	3.16	0.28	-1.38	2.41E-05	0.0002	eukaryotic translation initiation factor 4 gamma, 3
EIF4H	-3.07	-1.32	0.3	5.55E-06	0.0002	eukaryotic translation initiation factor 4H
ELMSAN1	3.1	0.43	-1.2	6.29E-06	0.0002	ELM2 and Myb/SANT-like domain containing 1
EMILIN2	3.15	0.28	-1.38	3.71E-06	0.0002	elastin microfibril interfacer 2
EMILIN3	3.3	0.32	-1.4	6.98E-06	0.0002	elastin microfibril interfacer 3
EMP3	-3.05	-1.33	0.28	1.21E-05	0.0002	epithelial membrane protein 3
ENDOG	-3.37	-1.31	0.44	1.24E-05	0.0002	endonuclease G
ENO1	3.41	0.32	-1.44	1.41E-05	0.0002	enolase 1, (alpha)
ENPEP	3.41	0.32	-1.44	3.84E-05	0.0003	glutamyl aminopeptidase (aminopeptidase A)
ENTPD5	3.26	0.32	-1.38	3.38E-05	0.0003	ectonucleoside triphosphate diphosphohydrolase 5
EPHB2	3.36	0.32	-1.43	1.37E-05	0.0002	EPH receptor B2
EPHB4	3.16	0.37	-1.29	2.07E-05	0.0002	EPH receptor B4
EPHX3	3.02	0.39	-1.2	1.08E-05	0.0002	epoxide hydrolase 3
PIN-WFDC6; WFD[illegible]	-3.04	-1.3	0.31	1.69E-05	0.0002	EPPIN-WFDC6 readthrough; WAP four-disulfide core domain 6
EPWW6493	-3.18	-1.34	0.33	0.0001	0.0006	uncharacterized LOC100129831

ERAS	-3.06	-1.37	0.24	4.83E-05	0.0003	ES cell expressed Ras
ERC2-IT1	3.36	0.32	-1.43	5.86E-05	0.0004	ERC2 intronic transcript 1
RCC5; BIVM-ERCC	3.39	0.32	-1.44	5.47E-06	0.0002	excision repair cross-complementation group 5; BIVM-ERCC5 readthrough
ERF	-3.2	-1.31	0.36	9.78E-06	0.0002	Ets2 repressor factor
ERG	3.37	0.32	-1.43	8.46E-06	0.0002	v-ets avian erythroblastosis virus E26 oncogene homolog
ERH	-3.09	-1.4	0.23	0.0001	0.0007	enhancer of rudimentary homolog (Drosophila)
ERI3	-3.04	-1.28	0.32	6.83E-06	0.0002	ERI1 exoribonuclease family member 3
ERVH48-1	-3.11	-1.28	0.36	1.00E-05	0.0002	endogenous retrovirus group 48, member 1
ERVK13-1	-3.18	-1.35	0.32	2.17E-05	0.0002	endogenous retrovirus group K13, member 1
ESPNP	3.41	0.32	-1.44	0.0005	0.0020	espin pseudogene
ESR1	3.49	0.41	-1.39	5.64E-05	0.0004	estrogen receptor 1
ETHE1	3.44	0.34	-1.44	7.00E-06	0.0002	ethylmalonic encephalopathy 1
ETV6	-3.24	-1.33	0.36	6.20E-06	0.0002	ets variant 6
EVC	-3.04	-1.28	0.32	1.15E-05	0.0002	Ellis van Creveld protein
EVX1	3.37	0.32	-1.43	1.40E-05	0.0002	even-skipped homeobox 1
EVX2	3.39	0.36	-1.4	2.87E-06	0.0002	even-skipped homeobox 2
EXD3	-3.07	-1.34	0.28	1.20E-05	0.0002	exonuclease 3-5 domain containing 3
EXO1	3.49	0.38	-1.43	3.31E-06	0.0002	exonuclease 1
EXTL3	3.06	0.32	-1.29	1.30E-05	0.0002	exostosin-like glycosyltransferase 3
F8A2; F8A3; F8A1	-3.04	-1.28	0.32	8.29E-06	0.0002	coagulation factor VIII-associated 2; coagulation factor VIII-associated 3; coagulation factor VIII-associated 1
FAH	-3.01	-1.31	0.28	4.12E-05	0.0003	fumarylacetoacetate hydrolase (fumarylacetoacetase)
FAM101A	-3.08	-1.38	0.24	1.59E-05	0.0002	family with sequence similarity 101, member A
FAM107A	3.39	0.33	-1.43	4.88E-06	0.0002	family with sequence similarity 107, member A
FAM109A	3.43	0.4	-1.38	1.97E-05	0.0002	family with sequence similarity 109, member A
FAM118B	3.1	0.43	-1.2	1.18E-05	0.0002	family with sequence similarity 118, member B
FAM122A	-3.37	-1.43	0.32	3.59E-05	0.0003	family with sequence similarity 122A
FAM135B	-3.09	-1.28	0.35	9.98E-06	0.0002	family with sequence similarity 135, member B
FAM155B	-3.3	-1.37	0.35	1.85E-05	0.0002	family with sequence similarity 155, member B
AM156B; FAM156	-3.21	-1.35	0.34	0.0002	0.0010	family with sequence similarity 156, member B; family with sequence similarity 156, member A
AM156B; FAM156	-3.09	-1.28	0.35	0.0002	0.0010	family with sequence similarity 156, member B; family with sequence similarity 156, member A
FAM162B	-3.25	-1.38	0.32	4.45E-06	0.0002	family with sequence similarity 162, member B
FAM163B	3.33	0.32	-1.41	1.12E-05	0.0002	family with sequence similarity 163, member B
FAM171A1	-3	-1.31	0.27	1.49E-05	0.0002	family with sequence similarity 171, member A1
FAM171B	3.23	0.37	-1.32	5.61E-06	0.0002	family with sequence similarity 171, member B
FAM180B	3.63	0.43	-1.43	6.55E-05	0.0004	family with sequence similarity 180, member B
FAM195A	-3.12	-1.36	0.28	0.0001	0.0006	family with sequence similarity 195, member A
FAM19A4	-3.04	-1.28	0.32	2.59E-05	0.0003	family with sequence similarity 19 (chemokine (C-C motif)-like), member A4
FAM210B	-3.12	-1.36	0.28	2.65E-05	0.0003	family with sequence similarity 210, member B

FAM216B	-3.12	-1.36	0.28	9.52E-06	0.0002	family with sequence similarity 216, member B
FAM221A	3.28	0.32	-1.39	4.37E-06	0.0002	family with sequence similarity 221, member A
FAM230B; FAM230A	-3.05	-1.37	0.23	0.0002	0.0010	family with sequence similarity 230, member B (non-protein coding); family with sequence similarity 230, member A
FAM3D	-3.04	-1.28	0.32	5.12E-05	0.0004	family with sequence similarity 3, member D
FAM45B; FAM45A	-3.44	-1.42	0.36	2.46E-05	0.0002	family with sequence similarity 45, member A pseudogene; family with sequence similarity 45, member A
FAM46A	3.5	0.35	-1.46	4.03E-06	0.0002	family with sequence similarity 46, member A
FAM53A	-3.06	-1.33	0.28	1.11E-05	0.0002	family with sequence similarity 53, member A
FAM81B	3.44	0.32	-1.46	1.02E-05	0.0002	family with sequence similarity 81, member B
FAM83G	-3.41	-1.38	0.39	0.0002	0.0009	family with sequence similarity 83, member G
FAM86B1	-3.24	-1.41	0.29	5.60E-06	0.0002	family with sequence similarity 86, member B1
FAM86EP	-3.22	-1.36	0.32	0.0001	0.0006	family with sequence similarity 86, member A pseudogene
FAM8A1	-3.01	-1.31	0.28	1.97E-05	0.0002	family with sequence similarity 8, member A1
FAM98A	-3.41	-1.38	0.39	2.01E-05	0.0002	family with sequence similarity 98, member A
FAM99A	3.41	0.32	-1.44	6.93E-06	0.0002	family with sequence similarity 99, member A (non-protein coding)
FAR2P1	-3.43	-1.46	0.32	7.11E-05	0.0004	fatty acyl-CoA reductase 2 pseudogene 1
FASTKD1	-3.28	-1.36	0.35	5.04E-05	0.0003	FAST kinase domains 1
FAU; SYVN1	-3.18	-1.28	0.39	0.0001	0.0007	Finkel-Biskis-Reilly murine sarcoma virus (FBR-MuSV) ubiquitously expressed; synovial apoptosis inhibitor 1, synoviolin
FBXL14	-3.03	-1.3	0.3	0.0001	0.0007	F-box and leucine-rich repeat protein 14
FBXL16	-3.09	-1.35	0.28	2.58E-05	0.0003	F-box and leucine-rich repeat protein 16
FBXL19; SETD1A	-3.09	-1.35	0.28	2.57E-05	0.0003	F-box and leucine-rich repeat protein 19; SET domain containing 1A
FBXO16	-3.18	-1.31	0.36	1.03E-05	0.0002	F-box protein 16
FBXO6	3.26	0.32	-1.38	4.74E-05	0.0003	F-box protein 6
FBXW10	-3.16	-1.38	0.28	0.0005	0.0019	F-box and WD repeat domain containing 10
FBXW2	3.4	0.37	-1.39	6.46E-06	0.0002	F-box and WD repeat domain containing 2
FCER1A	-3.14	-1.35	0.3	2.26E-05	0.0002	Fc fragment of IgE, high affinity I, receptor for; alpha polypeptide
FCN2	-3.29	-1.31	0.41	2.11E-05	0.0002	ficolin (collagen/fibrinogen domain containing lectin) 2
FCRL1	-3.05	-1.36	0.24	2.36E-05	0.0002	Fc receptor-like 1
FCRL6	3.47	0.32	-1.47	3.65E-06	0.0002	Fc receptor-like 6
FER1L5	-3.24	-1.33	0.36	8.67E-06	0.0002	fer-1-like family member 5
FGF11	3.24	0.41	-1.29	5.65E-05	0.0004	fibroblast growth factor 11
FGF14	-3.04	-1.28	0.32	0.0002	0.0010	fibroblast growth factor 14
FGF22	3.26	0.32	-1.38	5.09E-06	0.0002	fibroblast growth factor 22
FGF23	3.52	0.43	-1.38	3.94E-06	0.0002	fibroblast growth factor 23
FHL1	-3.18	-1.38	0.29	5.54E-05	0.0004	four and a half LIM domains 1
FHL2	3.25	0.31	-1.39	7.51E-06	0.0002	four and a half LIM domains 2
FIGNL2	-3.01	-1.31	0.28	2.69E-05	0.0003	fidgetin-like 2
FILIP1	3.26	0.41	-1.29	9.92E-06	0.0002	filamin A interacting protein 1
FKBP14	-3.1	-1.36	0.27	4.70E-06	0.0002	FK506 binding protein 14

FKBP4	3.02	0.39	-1.2	1.95E-05	0.0002	FK506 binding protein 4
FKBPL	-3.01	-1.31	0.28	5.87E-06	0.0002	FK506 binding protein like
FKBPL	-3.01	-1.31	0.28	5.87E-06	0.0002	FK506 binding protein like
FLCN; PLD6	-3.22	-1.36	0.32	2.79E-06	0.0002	folliculin; phospholipase D family, member 6
FLJ31715	3.53	0.44	-1.38	7.72E-06	0.0002	uncharacterized FLJ31715
FLJ32154	-3.12	-1.36	0.28	5.02E-05	0.0003	uncharacterized protein FLJ32154
FLJ35816	-3	-1.31	0.28	1.90E-05	0.0002	FLJ35816 protein
FLJ40536	-3.14	-1.28	0.37	0.0004	0.0016	FLJ40536 protein
FLJ42220	-3.37	-1.36	0.39	3.46E-05	0.0003	FLJ42220 protein
FLJ44477	3.15	0.21	-1.44	3.61E-06	0.0002	FLJ44477 protein
FLT4	-3.16	-1.36	0.3	3.92E-06	0.0002	fms-related tyrosine kinase 4
FMO9P	3.13	0.26	-1.39	4.32E-06	0.0002	flavin containing monooxygenase 9 pseudogene
FNDC8	3.24	0.31	-1.38	4.11E-05	0.0003	fibronectin type III domain containing 8
FOXD3	3.37	0.32	-1.43	7.50E-06	0.0002	forkhead box D3
FOXJ1	3.02	0.39	-1.2	1.28E-05	0.0002	forkhead box J1
FOXQ1	-3.14	-1.31	0.34	1.99E-05	0.0002	forkhead box Q1
FPR3	-3.09	-1.35	0.28	0.0001	0.0006	formyl peptide receptor 3
[illegible]1JP; FRG1DP; FRG[illegible]	3.6	0.41	-1.44	8.04E-06	0.0002	FSHD region gene 1 family member J, pseudogene; FSHD region gene 1 family member D, pseudogene; FSHD region gene 1 family member C, pseudogene
FRMD6	3.4	0.38	-1.38	2.13E-05	0.0002	FERM domain containing 6
FSCN2	3.78	0.47	-1.44	5.35E-06	0.0002	fascin actin-bundling protein 2, retinal
FSHB	-3.01	-1.31	0.28	0.0002	0.0008	follicle stimulating hormone, beta polypeptide
FST	3.19	0.47	-1.2	0.0001	0.0006	follistatin
FTCD	-3.31	-1.36	0.36	4.88E-05	0.0003	formimidoyltransferase cyclodeaminase
FUT11	3.46	0.39	-1.4	8.20E-06	0.0002	fucosyltransferase 11 (alpha (1,3) fucosyltransferase)
FXYD1	3.37	0.32	-1.43	3.20E-06	0.0002	FXYD domain containing ion transport regulator 1
FXYD4	3.41	0.32	-1.44	8.09E-06	0.0002	FXYD domain containing ion transport regulator 4
FZD10	-3.09	-1.28	0.35	5.36E-06	0.0002	frizzled class receptor 10
FZD3	-3.04	-1.31	0.29	3.86E-05	0.0003	frizzled class receptor 3
FZD8	3.21	0.24	-1.44	3.00E-06	0.0002	frizzled class receptor 8
GABARAPL1	3.26	0.32	-1.38	5.02E-05	0.0003	GABA(A) receptor-associated protein like 1
GABARAPL3	3.26	0.32	-1.38	7.27E-06	0.0002	GABA(A) receptors associated protein like 3, pseudogene
GAD1	3.33	0.36	-1.38	2.50E-06	0.0002	glutamate decarboxylase 1
GAD2	3.55	0.38	-1.44	2.64E-06	0.0002	glutamate decarboxylase 2
GADD45G	3.11	0.35	-1.29	2.66E-05	0.0003	growth arrest and DNA-damage-inducible, gamma
GAL3ST4	-3.15	-1.31	0.35	1.58E-05	0.0002	galactose-3-O-sulfotransferase 4
GALE	-3.03	-1.3	0.3	0.0001	0.0006	UDP-galactose-4-epimerase
GAN	-3	-1.28	0.31	6.80E-05	0.0004	gigaxonin
GARNL3	-3	-1.28	0.31	1.04E-05	0.0002	GTPase activating Rap/RanGAP domain-like 3

GAS2L1	-3.24	-1.34	0.36	1.45E-05	0.0002	growth arrest-specific 2 like 1
GAS6-AS1	3.31	0.32	-1.4	2.99E-05	0.0003	GAS6 antisense RNA 1
GAS8	3.02	0.39	-1.2	2.59E-05	0.0003	growth arrest-specific 8
GAST	3.2	0.3	-1.38	3.89E-05	0.0003	gastrin
GATA1	-3.13	-1.28	0.36	3.06E-06	0.0002	GATA binding protein 1 (globin transcription factor 1)
GATA5	3.02	0.39	-1.2	3.37E-05	0.0003	GATA binding protein 5
GBGT1	-3.11	-1.36	0.27	9.60E-06	0.0002	globoside alpha-1,3-N-acetylgalactosaminyltransferase 1
GCOM2	-3.06	-1.33	0.28	0.0002	0.0008	GRINL1B complex locus 2, pseudogene
GDF5	-3.08	-1.31	0.31	3.43E-05	0.0003	growth differentiation factor 5
GEMIN2	-3.17	-1.43	0.23	4.26E-05	0.0003	gem nuclear organelle associated protein 2
GEMIN4	3.19	0.47	-1.2	3.41E-05	0.0003	gem nuclear organelle associated protein 4
GFER	3.26	0.32	-1.38	4.97E-06	0.0002	growth factor, augmenter of liver regeneration
GFI1B	-3.03	-1.36	0.24	0.0002	0.0008	growth factor independent 1B transcription repressor
GFRA1	-3.02	-1.34	0.26	0.0001	0.0006	GDNF family receptor alpha 1
GFRA4	3.4	0.38	-1.38	8.10E-06	0.0002	GDNF family receptor alpha 4
GGNBP1	3.41	0.32	-1.44	9.86E-06	0.0002	gametogenetin binding protein 1 (pseudogene)
GGT6	-3.13	-1.28	0.36	1.07E-05	0.0002	gamma-glutamyltransferase 6
GIMAP7	-3.09	-1.38	0.24	4.41E-05	0.0003	GTPase, IMAP family member 7
GIP	3.11	0.32	-1.31	1.58E-05	0.0002	gastric inhibitory polypeptide
GIT2	-3.21	-1.43	0.25	5.18E-05	0.0004	G protein-coupled receptor kinase interacting ArfGAP 2
GJA5	-3.22	-1.36	0.32	1.09E-05	0.0002	gap junction protein alpha 5
GLIPR1	-3.09	-1.32	0.31	3.62E-05	0.0003	GLI pathogenesis-related 1
GLIS3	3.54	0.43	-1.39	6.02E-06	0.0002	GLIS family zinc finger 3
GLTP	3.4	0.29	-1.47	5.51E-05	0.0004	glycolipid transfer protein
GLUD2	-3.43	-1.41	0.37	2.40E-05	0.0002	glutamate dehydrogenase 2
GM140	3.3	0.32	-1.4	6.25E-05	0.0004	uncharacterized LOC100287948
GNAQ	3.93	0.4	-1.57	0.0005	0.0019	guanine nucleotide binding protein (G protein), q polypeptide
GNB4	-3.02	-1.36	0.23	1.20E-05	0.0002	guanine nucleotide binding protein (G protein), beta polypeptide 4
GNE	-3.01	-1.31	0.28	4.80E-05	0.0003	glucosamine (UDP-N-acetyl)-2-epimerase/N-acetylmannosamine kinase
GNG13	3.08	0.42	-1.2	5.26E-06	0.0002	guanine nucleotide binding protein (G protein), gamma 13
GNG4	-3.04	-1.28	0.33	3.34E-06	0.0002	guanine nucleotide binding protein (G protein), gamma 4
GNG8	-3.06	-1.31	0.3	6.54E-05	0.0004	guanine nucleotide binding protein (G protein), gamma 8
GNRHR2	-3.04	-1.39	0.22	4.93E-05	0.0003	gonadotropin releasing hormone receptor 2, pseudogene
GOLGA2	3.21	0.3	-1.38	3.39E-06	0.0002	golgin A2
GOLGA6L2	-3.16	-1.41	0.25	9.15E-06	0.0002	golgin A6 family-like 2
GOLGA7	-3.22	-1.31	0.38	9.32E-05	0.0005	golgin A7
OLGA8CP; GOLGA8	-3.01	-1.31	0.28	7.54E-06	0.0002	golgin A8 family, member C, pseudogene; golgin A8 family, member D, pseudogene
8EP; GOLGA8F; GC	-3.09	-1.31	0.32	5.70E-06	0.0002	golgin A8 family, member C, pseudogene; golgin A8 family, member E, pseudogene; golgin A8 family, member F; golgin A8 family, member G; golgin A8 family, member D, pseudogene

.8EP; GOLGA8F; GC	-3.09	-1.31	0.32	5.70E-06	0.0002	golgin A8 family, member C, pseudogene; golgin A8 family, member E, pseudogene; golgin A8 family, member F; golgin A8 family, member G; golgin A8 family, member D, pseudogene
.8EP; GOLGA8F; GC	-3.06	-1.32	0.29	7.15E-06	0.0002	golgin A8 family, member C, pseudogene; golgin A8 family, member E, pseudogene; golgin A8 family, member F; golgin A8 family, member G; golgin A8 family, member D, pseudogene
DP; GOLGA8CP; G(	-3.06	-1.31	0.3	7.45E-06	0.0002	golgin A8 family, member D, pseudogene; golgin A8 family, member C, pseudogene; golgin A8 family, member E, pseudogene
GOLPH3	-3.17	-1.33	0.33	9.02E-06	0.0002	golgi phosphoprotein 3 (coat-protein)
GP5	3.1	0.21	-1.42	9.98E-06	0.0002	glycoprotein V (platelet)
GPATCH4	-3.01	-1.31	0.28	6.48E-05	0.0004	G-patch domain containing 4
GPIHBP1	3.32	0.3	-1.43	8.52E-06	0.0002	glycosylphosphatidylinositol anchored high density lipoprotein binding protein 1
GPR135; L3HYPDH	-3.04	-1.3	0.31	1.07E-05	0.0002	G protein-coupled receptor 135; L-3-hydroxyproline dehydratase (trans-)
GPR156	3.02	0.3	-1.29	7.80E-06	0.0002	G protein-coupled receptor 156
GPR35	-3.14	-1.3	0.36	7.04E-05	0.0004	G protein-coupled receptor 35
GPR52	-3.12	-1.36	0.28	5.71E-06	0.0002	G protein-coupled receptor 52
GPR85	3.12	0.2	-1.44	2.22E-05	0.0002	G protein-coupled receptor 85
GPSM3	-3.23	-1.41	0.28	8.54E-06	0.0002	G-protein signaling modulator 3
GRAPL; GRAP	3.67	0.43	-1.44	9.51E-06	0.0002	GRB2-related adaptor protein-like; GRB2-related adaptor protein
GRB2	-3.03	-1.3	0.3	0.0001	0.0006	growth factor receptor bound protein 2
GRIN1	3.46	0.39	-1.4	1.99E-05	0.0002	glutamate receptor, ionotropic, N-methyl D-aspartate 1
GRK1	-3.02	-1.35	0.25	6.33E-05	0.0004	G protein-coupled receptor kinase 1
GRK5	3.45	0.41	-1.38	9.50E-06	0.0002	G protein-coupled receptor kinase 5
GRM4	-3.23	-1.32	0.37	2.36E-05	0.0002	glutamate receptor, metabotropic 4
GSDMB	3.2	0.25	-1.43	9.73E-06	0.0002	gasdermin B
GSS	3.24	0.31	-1.38	2.73E-06	0.0002	glutathione synthetase
GSTA2	-3.23	-1.41	0.28	8.54E-05	0.0005	glutathione S-transferase alpha 2
GSTM4; GSTM2	3.25	0.5	-1.2	3.21E-05	0.0003	glutathione S-transferase mu 4; glutathione S-transferase mu 2 (muscle)
GSTP1	3.44	0.32	-1.46	5.76E-05	0.0004	glutathione S-transferase pi 1
GSX1	3.53	0.39	-1.43	2.00E-05	0.0002	GS homeobox 1
GTF2A1	-3.12	-1.34	0.3	2.04E-05	0.0002	general transcription factor IIA 1
GTF3C5	3.01	0.39	-1.2	2.90E-05	0.0003	general transcription factor IIIC subunit 5
GUCY1A3	-3.05	-1.38	0.23	4.26E-06	0.0002	guanylate cyclase 1, soluble, alpha 3
GZMA	-3.17	-1.28	0.38	1.50E-05	0.0002	granzyme A
GZMM	-3.05	-1.28	0.33	5.63E-06	0.0002	granzyme M
H1FX	3.28	0.33	-1.38	5.42E-06	0.0002	H1 histone family, member X
HABP4	-3.03	-1.32	0.28	3.69E-05	0.0003	hyaluronan binding protein 4
HACD2	3.3	0.32	-1.4	6.54E-06	0.0002	3-hydroxyacyl-CoA dehydratase 2
HAGH	3.7	0.51	-1.38	1.44E-05	0.0002	hydroxyacylglutathione hydrolase
HARBI1	-3.01	-1.31	0.28	5.16E-05	0.0004	harbinger transposase derived 1
HAUS1	-3.14	-1.28	0.37	1.39E-05	0.0002	HAUS augmin like complex subunit 1
HAUS2	-3.05	-1.33	0.28	5.47E-06	0.0002	HAUS augmin like complex subunit 2
HAX1	3.14	0.36	-1.29	1.17E-05	0.0002	HCLS1 associated protein X-1

HBB	-3.47	-1.43	0.36	9.34E-05	0.0005	hemoglobin, beta
HBE1	3.4	0.32	-1.44	9.92E-06	0.0002	hemoglobin, epsilon 1
HBZ	-3.08	-1.35	0.28	0.0001	0.0006	hemoglobin, zeta
HCRP1	-3.1	-1.36	0.27	1.46E-05	0.0002	hepatocellular carcinoma-related HCRP1
HDAC8	3.29	0.27	-1.44	7.72E-06	0.0002	histone deacetylase 8
HDHD2	-3.1	-1.36	0.27	9.29E-06	0.0002	haloacid dehalogenase-like hydrolase domain containing 2
HEATR6	3.05	0.16	-1.44	2.46E-05	0.0002	HEAT repeat containing 6
HEBP2	3.32	0.32	-1.41	3.13E-05	0.0003	heme binding protein 2
HECTD4	3.06	0.32	-1.29	3.54E-05	0.0003	HECT domain containing E3 ubiquitin protein ligase 4
HELB	3.28	0.32	-1.39	2.38E-05	0.0002	helicase (DNA) B
HELQ	-3.12	-1.36	0.28	1.03E-05	0.0002	helicase, POLQ-like
HERC5	-3.12	-1.36	0.28	0.0001	0.0006	HECT and RLD domain containing E3 ubiquitin protein ligase 5
HERPUD2	3.01	0.39	-1.2	1.19E-05	0.0002	HERPUD family member 2
HEXDC	3.23	0.26	-1.43	6.62E-06	0.0002	hexosaminidase (glycosyl hydrolase family 20, catalytic domain) containing
HEXIM1	-3.06	-1.33	0.28	5.98E-05	0.0004	hexamethylene bis-acetamide inducible 1
HEXIM2	3.04	0.23	-1.38	1.32E-05	0.0002	hexamethylene bis-acetamide inducible 2
HGS	3.07	0.24	-1.38	5.33E-06	0.0002	hepatocyte growth factor-regulated tyrosine kinase substrate
HHATL; CCDC13	-3.12	-1.33	0.31	2.63E-05	0.0003	hedgehog acyltransferase-like; coiled-coil domain containing 13
HIGD1B	3.34	0.36	-1.38	8.53E-06	0.0002	HIG1 hypoxia inducible domain family, member 1B
HINFP	3.39	0.32	-1.44	0.0002	0.0009	histone H4 transcription factor
HIP1	3.08	0.42	-1.2	0.0001	0.0005	huntingtin interacting protein 1
HIST1H2AC	3.52	0.43	-1.38	1.16E-05	0.0002	histone cluster 1, H2ac
HIST1H2AH	3.4	0.38	-1.38	2.03E-05	0.0002	histone cluster 1, H2ah
HIST1H2AK	3.61	0.47	-1.38	1.46E-05	0.0002	histone cluster 1, H2ak
ST1H2AL; HIST1H2	3	0.38	-1.2	1.22E-05	0.0002	histone cluster 1, H2al; histone cluster 1, H2bn
HIST1H3B	3.57	0.39	-1.44	1.41E-05	0.0002	histone cluster 1, H3b
HIST1H3C	-3.09	-1.28	0.35	2.74E-05	0.0003	histone cluster 1, H3c
ST1H3D; HIST1H2/	3.33	0.32	-1.41	3.73E-06	0.0002	histone cluster 1, H3d; histone cluster 1, H2ad
HIST1H4A	-3.31	-1.36	0.36	2.91E-05	0.0003	histone cluster 1, H4a
HIST1H4D	3.3	0.32	-1.4	0.0001	0.0006	histone cluster 1, H4d
HIST2H2BA; H2BFS	-3.08	-1.3	0.33	5.48E-05	0.0004	histone cluster 2, H2ba (pseudogene); H2B histone family, member S (pseudogene)
HIST2H2BF	3.3	0.32	-1.4	0.0001	0.0006	histone cluster 2, H2bf
HK1	3.36	0.36	-1.39	1.21E-05	0.0002	hexokinase 1
HLA-DMB	-3.06	-1.33	0.28	3.76E-05	0.0003	major histocompatibility complex, class II, DM beta
HLA-DMB	-3.11	-1.36	0.28	4.54E-05	0.0003	major histocompatibility complex, class II, DM beta
HMGB3P1	3.21	0.24	-1.44	1.62E-05	0.0002	high mobility group box 3 pseudogene 1
HMGN2	3.06	0.41	-1.2	3.28E-06	0.0002	high mobility group nucleosomal binding domain 2
HMGN2P46	3.33	0.32	-1.41	2.95E-05	0.0003	high mobility group nucleosomal binding domain 2 pseudogene 46

HMX1	-3.18	-1.41	0.26	9.97E-06	0.0002	H6 family homeobox 1
HNRNPUL1	3.15	0.24	-1.41	2.55E-05	0.0003	heterogeneous nuclear ribonucleoprotein U-like 1
HOXA13	3.41	0.32	-1.44	0.0001	0.0006	homeobox A13
HOXB8	-3.16	-1.31	0.35	2.33E-05	0.0002	homeobox B8
HOXC12	3.13	0.45	-1.2	1.13E-05	0.0002	homeobox C12
HOXD13	3.5	0.42	-1.39	2.43E-05	0.0002	homeobox D13
HOXD4	-3.11	-1.31	0.32	2.22E-05	0.0002	homeobox D4
HP1BP3	3.37	0.32	-1.43	1.80E-05	0.0002	heterochromatin protein 1, binding protein 3
HPCAL1	-3.16	-1.31	0.35	3.56E-06	0.0002	hippocalcin-like 1
HPDL	-3.15	-1.33	0.32	2.12E-05	0.0002	4-hydroxyphenylpyruvate dioxygenase-like
HPS4	-3.16	-1.38	0.28	4.39E-05	0.0003	Hermansky-Pudlak syndrome 4
HPS5	3.2	0.28	-1.4	8.97E-06	0.0002	Hermansky-Pudlak syndrome 5
HPSE	-3.05	-1.34	0.27	1.54E-05	0.0002	heparanase
HRH4	3.3	0.32	-1.4	2.00E-05	0.0002	histamine receptor H4
HRNR; RPTN	-3.43	-1.41	0.36	8.38E-06	0.0002	hornerin; repetin
HS3ST3A1	3.41	0.32	-1.44	1.06E-05	0.0002	heparan sulfate (glucosamine) 3-O-sulfotransferase 3A1
HS3ST5	3.43	0.31	-1.47	3.59E-05	0.0003	heparan sulfate (glucosamine) 3-O-sulfotransferase 5
HSBP1	3.33	0.32	-1.41	4.48E-05	0.0003	heat shock factor binding protein 1
HSF2BP	3.28	0.32	-1.39	1.56E-05	0.0002	heat shock transcription factor 2 binding protein
HSF4; FBXL8	-3.13	-1.28	0.36	3.13E-05	0.0003	heat shock transcription factor 4; F-box and leucine-rich repeat protein 8
HSFX2; HSFX1	3.09	0.34	-1.29	1.59E-05	0.0002	heat shock transcription factor family, X-linked 2; heat shock transcription factor family, X-linked 1
HSFX2; HSFX1	3.09	0.34	-1.29	1.59E-05	0.0002	heat shock transcription factor family, X-linked 2; heat shock transcription factor family, X-linked 1
HSP90AA5P	3.39	0.32	-1.44	6.04E-05	0.0004	heat shock protein 90kDa alpha (cytosolic), class A member 5, pseudogene
HSP90AB4P	-3.05	-1.31	0.3	1.30E-05	0.0002	heat shock protein 90kDa alpha (cytosolic), class B member 4, pseudogene
HSPA1A; HSPA1B	-3.25	-1.31	0.39	8.62E-05	0.0005	heat shock 70kDa protein 1A; heat shock 70kDa protein 1B
HSPA1A; HSPA1B	-3.22	-1.31	0.38	9.83E-05	0.0005	heat shock 70kDa protein 1A; heat shock 70kDa protein 1B
HSPA2	3.19	0.29	-1.39	1.41E-05	0.0002	heat shock 70kDa protein 2
HSPB1	-3.08	-1.39	0.23	1.45E-05	0.0002	heat shock 27kDa protein 1
HSPB3	-3.12	-1.36	0.28	4.91E-05	0.0003	heat shock 27kDa protein 3
HSPB6	-3.16	-1.38	0.28	2.76E-05	0.0003	heat shock protein, alpha-crystallin-related, B6
HSPB7	-3.13	-1.38	0.26	0.0002	0.0011	heat shock 27kDa protein family, member 7 (cardiovascular)
HTATIP2	-3.36	-1.38	0.36	0.0004	0.0016	HIV-1 Tat interactive protein 2
HTATSF1P2	3.52	0.43	-1.38	1.16E-05	0.0002	HIV-1 Tat specific factor 1 pseudogene 2
HTR1B	3.07	0.21	-1.4	6.00E-05	0.0004	5-hydroxytryptamine (serotonin) receptor 1B, G protein-coupled
HTR1D	3.35	0.3	-1.44	0.0001	0.0005	5-hydroxytryptamine (serotonin) receptor 1D, G protein-coupled
HTR1E	-3.24	-1.38	0.32	3.55E-05	0.0003	5-hydroxytryptamine (serotonin) receptor 1E, G protein-coupled
HTR6	-3.06	-1.33	0.28	0.0002	0.0008	5-hydroxytryptamine (serotonin) receptor 6, G protein-coupled
HTRA4	-3.1	-1.3	0.34	1.43E-05	0.0002	HtrA serine peptidase 4

HYAL2; TUSC2	-3.13	-1.38	0.26	2.17E-05	0.0002	hyaluronoglucosaminidase 2; tumor suppressor candidate 2
HYPM	-3.29	-1.31	0.4	5.67E-05	0.0004	huntingtin interacting protein M
IBSP	3.18	0.38	-1.29	4.54E-05	0.0003	integrin-binding sialoprotein
ICAM3	3.08	0.33	-1.29	3.59E-06	0.0002	intercellular adhesion molecule 3
ICE2	-3.24	-1.33	0.36	1.00E-05	0.0002	interactor of little elongation complex ELL subunit 2
IDS	-3.13	-1.32	0.32	5.00E-05	0.0003	iduronate 2-sulfatase
IER3	3.07	0.33	-1.29	7.35E-05	0.0004	immediate early response 3
IFI27L1	-3.13	-1.28	0.36	3.49E-05	0.0003	interferon, alpha-inducible protein 27-like 1
IFI35	-3.01	-1.3	0.29	1.45E-05	0.0002	interferon-induced protein 35
IFIT3	-3.03	-1.33	0.27	5.65E-06	0.0002	interferon-induced protein with tetratricopeptide repeats 3
IFNA16	-3.16	-1.38	0.28	3.53E-05	0.0003	interferon, alpha 16
IFNLR1	3.02	0.39	-1.2	7.43E-06	0.0002	interferon, lambda receptor 1
IFT172	3.26	0.32	-1.38	3.43E-06	0.0002	intraflagellar transport 172
IGFBP2	3.26	0.41	-1.29	5.00E-06	0.0002	insulin like growth factor binding protein 2
IGFBP6	3.16	0.37	-1.29	1.55E-05	0.0002	insulin like growth factor binding protein 6
IGFBPL1	3.42	0.37	-1.4	4.33E-05	0.0003	insulin like growth factor binding protein-like 1
IGFN1	-3.02	-1.3	0.3	3.04E-05	0.0003	immunoglobulin-like and fibronectin type III domain containing 1
'3-53; IGHV3-35; IC	3.38	0.36	-1.4	3.06E-06	0.0002	immunoglobulin heavy constant alpha 1; immunoglobulin heavy variable 3-72; immunoglobulin heavy variable 3-53; immunoglobulin heavy variable 3-35 (non-functional); immunoglobulin heavy variable 3-38 (non-functional); immunoglobulin heavy variable 3/OR16-7 (pseudogene)
GHV3-53; IGHV3-3	3.44	0.35	-1.44	2.92E-06	0.0002	immunoglobulin heavy variable 3-53; immunoglobulin heavy variable 3-35 (non-functional)
GKV1D-33; IGKV1D	-3.23	-1.35	0.35	7.47E-05	0.0004	immunoglobulin kappa variable 1D-43; immunoglobulin kappa variable 1D-33; immunoglobulin kappa variable 1D-17; immunoglobulin kappa variable 1-33
IGKV2D-30	-3.14	-1.37	0.28	1.04E-05	0.0002	immunoglobulin kappa variable 2D-30
KV2D-30; IGKV2D-	-3.15	-1.38	0.27	2.08E-05	0.0002	immunoglobulin kappa variable 2D-30; immunoglobulin kappa variable 2D-24 (non-functional)
GLV5-45; IGLV1-40	3.13	0.26	-1.39	5.67E-06	0.0002	[illegible]
IGSF8	-3.41	-1.37	0.4	1.86E-05	0.0002	immunoglobulin superfamily, member 8
IGSF9	-3.18	-1.28	0.39	3.89E-06	0.0002	immunoglobulin superfamily, member 9
IHH	3.1	0.43	-1.2	6.16E-05	0.0004	indian hedgehog
IKBKE	-3.26	-1.38	0.32	0.0001	0.0006	inhibitor of kappa light polypeptide gene enhancer in B-cells, kinase epsilon
IKZF1	3.41	0.37	-1.4	7.23E-06	0.0002	IKAROS family zinc finger 1
IL12A	-3.01	-1.31	0.28	5.37E-06	0.0002	interleukin 12A
IL17B	3.19	0.47	-1.2	3.68E-06	0.0002	interleukin 17B
IL17RC	3.38	0.33	-1.43	8.37E-06	0.0002	interleukin 17 receptor C
IL22RA2	-3.06	-1.37	0.24	6.85E-06	0.0002	interleukin 22 receptor, alpha 2
IL23A	3.18	0.24	-1.43	8.99E-06	0.0002	interleukin 23, alpha subunit p19
IL27	3.09	0.42	-1.2	0.0002	0.0008	interleukin 27
IL33	-3.15	-1.33	0.32	4.14E-05	0.0003	interleukin 33
IL34	-3.12	-1.36	0.28	2.24E-05	0.0002	interleukin 34
ILDR2	3.25	0.31	-1.39	3.48E-06	0.0002	immunoglobulin-like domain containing receptor 2
IMP4	-3.34	-1.3	0.44	1.04E-05	0.0002	IMP4 homolog, U3 small nucleolar ribonucleoprotein

IMPA1	3.55	0.43	-1.4	1.68E-05	0.0002	inositol(myo)-1(or 4)-monophosphatase 1
ING1	-3.11	-1.28	0.36	4.66E-05	0.0003	inhibitor of growth family member 1
ING5	3.46	0.39	-1.4	5.65E-06	0.0002	inhibitor of growth family member 5
ING5	3.21	0.39	-1.29	1.30E-05	0.0002	inhibitor of growth family member 5
INHBB	3.26	0.32	-1.38	6.24E-06	0.0002	inhibin beta B
INIP	-3.16	-1.38	0.28	4.84E-06	0.0002	INTS3 and NABP interacting protein
INO80E	-3.12	-1.36	0.28	1.14E-05	0.0002	INO80 complex subunit E
INPP5K	3.34	0.36	-1.38	5.15E-05	0.0004	inositol polyphosphate-5-phosphatase K
INSL5	-3.36	-1.38	0.36	0.0001	0.0006	insulin-like 5
INTS4P2; INTS4P1	-3.1	-1.31	0.32	7.21E-06	0.0002	integrator complex subunit 4 pseudogene 2; integrator complex subunit 4 pseudogene 1
INTS6	-3.26	-1.38	0.32	3.98E-05	0.0003	integrator complex subunit 6
IPMK	3.72	0.45	-1.44	2.15E-05	0.0002	inositol polyphosphate multikinase
IPO5	3.21	0.39	-1.29	3.75E-06	0.0002	importin 5
IPO7	3.2	0.3	-1.38	1.73E-05	0.0002	importin 7
IPO9	-3.02	-1.3	0.3	2.32E-05	0.0002	importin 9
IQGAP3	3.2	0.28	-1.4	5.63E-06	0.0002	IQ motif containing GTPase activating protein 3
IQSEC3	3.02	0.39	-1.2	0.0001	0.0006	IQ motif and Sec7 domain 3
IRAK1	-3.08	-1.4	0.23	1.85E-05	0.0002	interleukin 1 receptor associated kinase 1
ISCA2	-3.44	-1.46	0.32	8.92E-06	0.0002	iron-sulfur cluster assembly 2
ISG20	-3.06	-1.32	0.29	5.89E-06	0.0002	interferon stimulated exonuclease gene 20kDa
ISLR	3.26	0.32	-1.38	8.28E-06	0.0002	immunoglobulin superfamily containing leucine-rich repeat
ITGB1	3.46	0.39	-1.4	2.68E-06	0.0002	integrin beta 1
ITPKC	3.42	0.39	-1.38	2.79E-06	0.0002	inositol-trisphosphate 3-kinase C
JPH1	3.49	0.32	-1.48	8.07E-06	0.0002	junctophilin 1
JPH2	3.06	0.32	-1.29	1.78E-05	0.0002	junctophilin 2
JRK	-3.11	-1.31	0.32	0.0002	0.0008	Jrk helix-turn-helix protein
JUNB	3.32	0.44	-1.29	0.0001	0.0006	jun B proto-oncogene
KANK1	-3.09	-1.36	0.26	1.17E-05	0.0002	KN motif and ankyrin repeat domains 1
KAT7	-3.22	-1.28	0.4	1.52E-05	0.0002	K(lysine) acetyltransferase 7
KATNA1	-3.16	-1.38	0.28	0.0001	0.0005	katanin p60 (ATPase containing) subunit A 1
KBTBD13	-3.05	-1.33	0.28	7.55E-05	0.0004	kelch repeat and BTB (POZ) domain containing 13
KBTBD8	-3.05	-1.32	0.29	0.0002	0.0009	kelch repeat and BTB (POZ) domain containing 8
KCNAB3	3.02	0.39	-1.2	6.05E-06	0.0002	potassium channel, voltage gated subfamily A regulatory beta subunit 3
KCNC4	3.38	0.36	-1.4	2.30E-05	0.0002	potassium channel, voltage gated Shaw related subfamily C, member 4
KCNG1	3.21	0.28	-1.4	2.36E-05	0.0002	potassium channel, voltage gated modifier subfamily G, member 1
KCNJ8	-3.12	-1.36	0.28	0.0002	0.0008	potassium channel, inwardly rectifying subfamily J, member 8
KCNK13	3.33	0.33	-1.4	2.05E-05	0.0002	potassium channel, two pore domain subfamily K, member 13
KCNK18	3.37	0.37	-1.38	1.31E-05	0.0002	potassium channel, two pore domain subfamily K, member 18

KCNK9	-3.06	-1.34	0.28	1.04E-05	0.0002	potassium channel, two pore domain subfamily K, member 9
KCNQ2	-3.04	-1.3	0.31	8.27E-05	0.0005	potassium channel, voltage gated KQT-like subfamily Q, member 2
KCNS3	-3.08	-1.36	0.26	3.00E-05	0.0003	potassium voltage-gated channel, modifier subfamily S, member 3
KCTD12	3.52	0.43	-1.38	9.65E-06	0.0002	potassium channel tetramerization domain containing 12
KCTD13	-3.23	-1.41	0.28	0.0001	0.0006	potassium channel tetramerization domain containing 13
KCTD2	-3.03	-1.32	0.28	7.78E-06	0.0002	potassium channel tetramerization domain containing 2
KCTD20	-3.04	-1.28	0.32	9.13E-06	0.0002	potassium channel tetramerization domain containing 20
KCTD21	3.3	0.33	-1.39	4.30E-06	0.0002	potassium channel tetramerization domain containing 21
KCTD4	3.02	0.39	-1.2	0.0001	0.0007	potassium channel tetramerization domain containing 4
KCTD7	-3.4	-1.38	0.38	1.58E-05	0.0002	potassium channel tetramerization domain containing 7
KDM5A	3.1	0.43	-1.2	1.65E-05	0.0002	lysine (K)-specific demethylase 5A
KDM8	-3.04	-1.36	0.24	3.83E-05	0.0003	lysine (K)-specific demethylase 8
KEAP1	-3.11	-1.31	0.33	9.64E-06	0.0002	kelch-like ECH-associated protein 1
KHDRBS3	-3.13	-1.28	0.36	4.10E-05	0.0003	KH domain containing, RNA binding, signal transduction associated 3
KIAA0355	3.18	0.25	-1.42	5.40E-06	0.0002	KIAA0355
KIAA0391	-3.03	-1.36	0.24	5.55E-06	0.0002	KIAA0391
KIAA0895L	-3.43	-1.42	0.36	2.02E-05	0.0002	KIAA0895-like
KIAA1549L	3.16	0.23	-1.43	1.01E-05	0.0002	KIAA1549-like
KIAA1671	-3.26	-1.31	0.39	0.0002	0.0007	KIAA1671
KIAA1683	3.63	0.43	-1.43	7.19E-06	0.0002	KIAA1683
KIAA1755	3.35	0.34	-1.4	5.78E-05	0.0004	KIAA1755
KIAA2013	-3.25	-1.39	0.31	3.12E-05	0.0003	KIAA2013
KIF18B	-3.17	-1.41	0.26	1.66E-05	0.0002	kinesin family member 18B
KIF19	3.37	0.35	-1.4	9.54E-06	0.0002	kinesin family member 19
KIF1C	3.02	0.39	-1.2	0.0002	0.0008	kinesin family member 1C
KIF2C	-3.11	-1.36	0.27	6.51E-06	0.0002	kinesin family member 2C
KIF3B	-3.01	-1.33	0.26	4.45E-06	0.0002	kinesin family member 3B
KIFAP3	-3.11	-1.41	0.23	2.67E-05	0.0003	kinesin-associated protein 3
KIR3DL3; KIR2DL2	3.91	0.49	-1.48	9.25E-06	0.0002	killer cell immunoglobulin-like receptor, three domains, long cytoplasmic tail, 3; killer cell immunoglobulin-like receptor, two domains, long cytoplasmic tail, 2
KIRREL3-AS3	3.1	0.43	-1.2	1.08E-05	0.0002	KIRREL3 antisense RNA 3
KITLG	-3.33	-1.37	0.36	1.05E-05	0.0002	KIT ligand
KLF15	3.42	0.39	-1.38	4.72E-05	0.0003	Kruppel-like factor 15
KLF4	3.26	0.32	-1.38	2.18E-05	0.0002	Kruppel-like factor 4 (gut)
KLF9	3.23	0.3	-1.39	6.53E-06	0.0002	Kruppel-like factor 9
KLHDC4	-3.11	-1.31	0.32	0.0004	0.0017	kelch domain containing 4
KLHDC7A	3.44	0.38	-1.4	7.91E-06	0.0002	kelch domain containing 7A
KLHL11	-3.22	-1.28	0.4	1.95E-05	0.0002	kelch-like family member 11
KLHL22	3.23	0.29	-1.4	3.29E-05	0.0003	kelch-like family member 22

KLHL33	-3.12	-1.41	0.23	3.56E-05	0.0003	kelch-like family member 33
KLK1	3.07	0.42	-1.2	1.57E-05	0.0002	kallikrein 1
KLK10	3.19	0.47	-1.2	5.92E-05	0.0004	kallikrein related peptidase 10
KLK11	3.01	0.39	-1.2	5.22E-06	0.0002	kallikrein related peptidase 11
KLK15	3.16	0.28	-1.38	5.62E-06	0.0002	kallikrein related peptidase 15
KLK9; KLK8	-3.1	-1.38	0.25	4.81E-05	0.0003	kallikrein related peptidase 9; kallikrein related peptidase 8
KPNA2	3.41	0.32	-1.44	3.88E-05	0.0003	karyopherin alpha 2 (RAG cohort 1, importin alpha 1)
KPNA2	3.47	0.32	-1.47	6.93E-05	0.0004	karyopherin alpha 2 (RAG cohort 1, importin alpha 1)
KRT18P20	3.63	0.43	-1.43	1.57E-05	0.0002	keratin 18 pseudogene 20
KRT18P55	-3.01	-1.28	0.31	7.83E-05	0.0005	keratin 18 pseudogene 55
KRT33B	-3.01	-1.38	0.21	1.07E-05	0.0002	keratin 33B, type I
KRT82	3.2	0.28	-1.4	1.97E-05	0.0002	keratin 82, type II
KRT9	3.2	0.39	-1.29	9.60E-06	0.0002	keratin 9, type I
KRTAP12-1	-3.23	-1.41	0.28	1.08E-05	0.0002	keratin associated protein 12-1
RTAP1-4; KRTAP1-	-3.19	-1.4	0.28	0.0001	0.0006	keratin associated protein 1-4; keratin associated protein 1-5
TAP19-6; KRTAP19	-3.06	-1.4	0.22	2.10E-05	0.0002	keratin associated protein 19-6; keratin associated protein 19-7
KRTAP23-1	3.3	0.32	-1.4	8.83E-06	0.0002	keratin associated protein 23-1
KRTAP5-6	-3.04	-1.28	0.32	6.40E-06	0.0002	keratin associated protein 5-6
RTAP5-9; KRTAP5-	3.37	0.35	-1.4	7.23E-05	0.0004	keratin associated protein 5-9; keratin associated protein 5-8
KRTAP6-1	-3.25	-1.28	0.42	2.43E-05	0.0002	keratin associated protein 6-1
KRTAP9-3	3.21	0.27	-1.41	9.45E-06	0.0002	keratin associated protein 9-3
KRTCAP2	-3.01	-1.31	0.28	2.09E-05	0.0002	keratinocyte associated protein 2
KXD1	3.49	0.42	-1.38	8.03E-06	0.0002	KxDL motif containing 1
KY	3.65	0.43	-1.44	3.87E-06	0.0002	kyphoscoliosis peptidase
L3MBTL1	-3.07	-1.33	0.29	1.11E-05	0.0002	l(3)mbt-like 1 (Drosophila)
LACC1	-3.22	-1.36	0.33	8.50E-05	0.0005	laccase (multicopper oxidoreductase) domain containing 1
LAMA4	3.26	0.32	-1.38	9.34E-06	0.0002	laminin, alpha 4
LARP1	3.43	0.4	-1.38	3.80E-06	0.0002	La ribonucleoprotein domain family, member 1
LAYN	3.33	0.32	-1.41	3.35E-06	0.0002	layilin
LCAT; SLC12A4	-3.22	-1.28	0.4	0.0002	0.0010	lecithin-cholesterol acyltransferase; solute carrier family 12 (potassium/chloride transporter), member 4
LCE1E	3.7	0.51	-1.38	4.65E-06	0.0002	late cornified envelope 1E
LCMT1	-3.15	-1.33	0.32	0.0001	0.0005	leucine carboxyl methyltransferase 1
LCMT2	3.16	0.26	-1.4	2.46E-05	0.0002	leucine carboxyl methyltransferase 2
LCN6	-3.1	-1.28	0.35	5.18E-06	0.0002	lipocalin 6
LCP2	-3.11	-1.31	0.33	9.60E-06	0.0002	lymphocyte cytosolic protein 2
LDB1	-3.09	-1.28	0.35	1.15E-05	0.0002	LIM domain binding 1
LDLR	3.1	0.23	-1.4	1.72E-05	0.0002	low density lipoprotein receptor
LDOC1L	-3.27	-1.39	0.32	4.89E-05	0.0003	leucine zipper, down-regulated in cancer 1-like

LENG9	3.33	0.32	-1.41	5.56E-06	0.0002	leukocyte receptor cluster (LRC) member 9
LGALS8-AS1	-3.11	-1.3	0.34	6.73E-05	0.0004	LGALS8 antisense RNA 1
LGALS9C	3.3	0.32	-1.4	4.89E-06	0.0002	lectin, galactoside-binding, soluble, 9C
LGALSL	-3.02	-1.31	0.28	0.0001	0.0006	lectin, galactoside-binding-like
LGI3	-3.37	-1.3	0.46	2.65E-05	0.0003	leucine-rich repeat LGI family, member 3
LIF	-3.24	-1.33	0.36	2.20E-05	0.0002	leukemia inhibitory factor
LILRA1	3.33	0.35	-1.38	2.98E-05	0.0003	leukocyte immunoglobulin-like receptor, subfamily A (with TM domain), member 1
LILRB1	3.63	0.43	-1.43	1.11E-05	0.0002	leukocyte immunoglobulin-like receptor, subfamily B (with TM and ITIM domains), member 1
LIMD1-AS1	-3.72	-1.28	0.61	6.91E-05	0.0004	LIMD1 antisense RNA 1
LIN9	-3.09	-1.31	0.32	1.57E-05	0.0002	lin-9 DREAM MuvB core complex component
LINC00152	3.01	0.21	-1.38	1.03E-05	0.0002	long intergenic non-protein coding RNA 152
LINC00268	-3.16	-1.36	0.3	7.21E-06	0.0002	long intergenic non-protein coding RNA 268
LINC00310	3.11	0.21	-1.43	9.67E-06	0.0002	long intergenic non-protein coding RNA 310
LINC00313	3.16	0.28	-1.38	1.02E-05	0.0002	long intergenic non-protein coding RNA 313
LINC00472	3.11	0.32	-1.31	3.40E-05	0.0003	long intergenic non-protein coding RNA 472
LINC00518	-3.23	-1.43	0.26	1.75E-05	0.0002	long intergenic non-protein coding RNA 518
LINC00523	-3.12	-1.36	0.28	1.14E-05	0.0002	long intergenic non-protein coding RNA 523
LINC00544	3.3	0.32	-1.4	7.07E-05	0.0004	long intergenic non-protein coding RNA 544
LINC00649	-3.14	-1.3	0.36	6.71E-06	0.0002	long intergenic non-protein coding RNA 649
LINC00656	-3.03	-1.32	0.28	6.38E-06	0.0002	long intergenic non-protein coding RNA 656
LINC00998	-3.13	-1.28	0.36	5.08E-05	0.0003	long intergenic non-protein coding RNA 998
NC01106; LINC011	-3.12	-1.34	0.31	2.02E-05	0.0002	long intergenic non-protein coding RNA 1106; long intergenic non-protein coding RNA 1123
LINC01123	3.35	0.42	-1.32	2.06E-05	0.0002	long intergenic non-protein coding RNA 1123
NC01287; ERVFC1-	3.26	0.28	-1.43	7.94E-06	0.0002	long intergenic non-protein coding RNA 1287; endogenous retrovirus group FC1, member 1
LINC01446	3.14	0.36	-1.29	3.93E-06	0.0002	long intergenic non-protein coding RNA 1446
LIPE	3.37	0.32	-1.43	2.68E-05	0.0003	lipase, hormone-sensitive
LLPH	-3.01	-1.28	0.31	4.23E-05	0.0003	LLP homolog, long-term synaptic facilitation (Aplysia)
LMCD1	3.28	0.33	-1.38	2.79E-06	0.0002	LIM and cysteine-rich domains 1
LMOD3	-3.06	-1.33	0.28	6.10E-05	0.0004	leiomodin 3 (fetal)
LOC100049716	3.27	0.24	-1.47	9.80E-06	0.0002	uncharacterized LOC100049716
LOC100128356	-3.03	-1.3	0.3	1.20E-05	0.0002	protein transactivated by hepatitis B virus E antigen
LOC100129884	3.12	0.34	-1.3	9.74E-06	0.0002	uncharacterized LOC100129884
LOC100130264	-3.06	-1.31	0.3	6.27E-06	0.0002	uncharacterized LOC100130264
LOC100130370	-3.1	-1.36	0.27	2.72E-05	0.0003	uncharacterized LOC100130370
LOC100130507	-3.11	-1.31	0.32	1.22E-05	0.0002	uncharacterized LOC100130507
LOC100131195	3.1	0.23	-1.4	3.60E-05	0.0003	uncharacterized LOC100131195
LOC100131510	3.24	0.49	-1.2	4.13E-06	0.0002	uncharacterized LOC100131510
LOC100132014	-3.31	-1.36	0.36	4.97E-05	0.0003	uncharacterized LOC100132014

LOC100132319	-3.07	-1.36	0.25	0.0001	0.0007	uncharacterized LOC100132319
LOC100287792	3.38	0.38	-1.38	9.51E-06	0.0002	uncharacterized LOC100287792
LOC100288208	3.59	0.44	-1.4	2.89E-05	0.0003	uncharacterized LOC100288208
LOC100288884	3.3	0.34	-1.38	3.30E-06	0.0002	uncharacterized LOC100288884
LOC100506730	3.64	0.39	-1.47	3.57E-06	0.0002	uncharacterized LOC100506730
LOC100996325	-3.16	-1.38	0.28	3.40E-05	0.0003	uncharacterized LOC100996325
LOC100996455	3.31	0.29	-1.44	7.38E-06	0.0002	uncharacterized LOC100996455
LOC101927263	3.26	0.32	-1.38	1.62E-05	0.0002	uncharacterized LOC101927263
LOC101928673	-3.13	-1.28	0.36	9.97E-06	0.0002	uncharacterized LOC101928673
LOC101928879	-3.18	-1.36	0.3	4.78E-06	0.0002	COMM domain-containing protein 5-like
LOC101928885	-3.03	-1.28	0.32	1.16E-05	0.0002	uncharacterized LOC101928885
LOC101930026	3.46	0.39	-1.4	4.23E-06	0.0002	uncharacterized LOC101930026
LOC101930026	3.44	0.39	-1.39	5.72E-06	0.0002	uncharacterized LOC101930026
LOC102723462	3.37	0.32	-1.43	1.02E-05	0.0002	uncharacterized LOC102723462
LOC102723968	-3.06	-1.39	0.23	0.0009	0.0034	uncharacterized LOC102723968
)2724970; LOC1001	3.28	0.33	-1.38	5.68E-05	0.0004	putative uncharacterized protein FLJ35883; uncharacterized LOC100133616
)2724970; LOC1001	3.28	0.33	-1.38	5.68E-05	0.0004	putative uncharacterized protein FLJ35883; uncharacterized LOC100133616
102724984; LOC72	3.01	0.21	-1.38	1.21E-05	0.0002	uncharacterized protein KIAA2013; KIAA2013 pseudogene
LOC102725001	3.2	0.28	-1.4	1.06E-05	0.0002	putative ankyrin repeat domain-containing protein 20A5
2725258; DKFZP77	-3.08	-1.3	0.33	0.0002	0.0009	uncharacterized LOC102725258; uncharacterized LOC643162
105369190; LOC17	3.45	0.38	-1.4	1.03E-05	0.0002	uncharacterized LOC105369190; uncharacterized LOC171391
LOC105369520	-3.12	-1.36	0.28	5.86E-06	0.0002	uncharacterized LOC105369520
LOC105370792	3.11	0.22	-1.41	3.13E-06	0.0002	uncharacterized LOC105370792
)5371034; LOC105:	3.52	0.43	-1.38	7.89E-06	0.0002	uncharacterized LOC105371033; uncharacterized LOC105371034; uncharacterized LOC105371035; uncharacterized LOC105371030
)5371034; LOC105:	3.52	0.43	-1.38	7.89E-06	0.0002	uncharacterized LOC105371033; uncharacterized LOC105371034; uncharacterized LOC105371035; uncharacterized LOC105371030
)5371034; LOC105:	3.52	0.43	-1.38	7.89E-06	0.0002	uncharacterized LOC105371033; uncharacterized LOC105371034; uncharacterized LOC105371035; uncharacterized LOC105371030
4; LOC105371035; l	3.73	0.51	-1.39	2.25E-05	0.0002	uncharacterized LOC105371034; uncharacterized LOC105371035; uncharacterized LOC105371030
4; LOC105371035; l	3.56	0.43	-1.4	7.13E-05	0.0004	uncharacterized LOC105371034; uncharacterized LOC105371035; uncharacterized LOC105371030
4; LOC105371035; l	3.56	0.43	-1.4	7.13E-05	0.0004	uncharacterized LOC105371034; uncharacterized LOC105371035; uncharacterized LOC105371030
C105371215; FLJ37	3.26	0.32	-1.38	2.35E-05	0.0002	uncharacterized LOC105371215; uncharacterized LOC642691
)5375091; LOC1002	3.23	0.29	-1.4	1.02E-05	0.0002	uncharacterized LOC105375091; uncharacterized LOC100287869
LOC105378913	3.26	0.32	-1.38	2.73E-05	0.0003	uncharacterized LOC105378913
LOC150596	-3.32	-1.45	0.28	6.67E-05	0.0004	uncharacterized LOC150596
LOC157740	3.86	0.52	-1.43	5.59E-06	0.0002	uncharacterized protein C8orf9
401281; LOC10537	-3.17	-1.31	0.35	2.36E-05	0.0002	uncharacterized LOC401281; uncharacterized LOC105378085
401589; LOC10012	-3.02	-1.28	0.32	1.53E-05	0.0002	uncharacterized LOC401589; uncharacterized LOC100129860
401589; LOC10012	-3.02	-1.28	0.32	1.53E-05	0.0002	uncharacterized LOC401589; uncharacterized LOC100129860
LOC441728	-3.2	-1.31	0.36	9.56E-06	0.0002	golgin-like

LOC494141	-3.19	-1.36	0.31	4.30E-05	0.0003	solute carrier family 25, member 51 pseudogene
LOC554207	-3.06	-1.31	0.3	1.03E-05	0.0002	uncharacterized LOC554207
LOC643549	3.52	0.43	-1.38	3.49E-06	0.0002	uncharacterized LOC643549
LOC644172	-3.08	-1.36	0.26	3.97E-05	0.0003	mitogen-activated protein kinase 8 interacting protein 1 pseudogene
LOC645261	3.59	0.46	-1.38	1.09E-05	0.0002	PP565
LOC646471	-3.31	-1.33	0.4	0.0005	0.0021	uncharacterized LOC646471
LOC653653	3.26	0.32	-1.38	7.03E-05	0.0004	adaptor-related protein complex 1, sigma 2 subunit pseudogene
LOC728283	-3.36	-1.38	0.36	1.33E-05	0.0002	uncharacterized LOC728283
LOC90768	3.42	0.39	-1.38	1.87E-05	0.0002	uncharacterized LOC90768
LOXL3	3.19	0.29	-1.38	2.57E-05	0.0003	lysyl oxidase-like 3
LPAL2	3.1	0.43	-1.2	0.0002	0.0009	lipoprotein, Lp(a)-like 2, pseudogene
LRFN4	3.1	0.43	-1.2	3.13E-06	0.0002	leucine rich repeat and fibronectin type III domain containing 4
LRIG1	-3.21	-1.41	0.27	0.0002	0.0009	leucine-rich repeats and immunoglobulin-like domains 1
LRIT3	-3.01	-1.31	0.28	0.0002	0.0009	leucine-rich repeat, immunoglobulin-like and transmembrane domains 3
LRRC10	-3.01	-1.38	0.21	1.02E-05	0.0002	leucine rich repeat containing 10
LRRC27	3.16	0.22	-1.44	1.03E-05	0.0002	leucine rich repeat containing 27
LRRC34	3.46	0.32	-1.47	2.85E-05	0.0003	leucine rich repeat containing 34
LRRC4	-3.25	-1.38	0.32	2.22E-05	0.0002	leucine rich repeat containing 4
LRRC4C	3.26	0.32	-1.38	9.94E-06	0.0002	leucine rich repeat containing 4C
LRRC57	3.19	0.47	-1.2	3.47E-06	0.0002	leucine rich repeat containing 57
LRRC58	3.06	0.32	-1.29	3.92E-06	0.0002	leucine rich repeat containing 58
LRRC6	3.29	0.3	-1.41	2.57E-05	0.0003	leucine rich repeat containing 6
LRRC8C	3.3	0.43	-1.29	1.07E-05	0.0002	leucine rich repeat containing 8 family, member C
LRRIQ4	3.37	0.28	-1.47	1.13E-05	0.0002	leucine-rich repeats and IQ motif containing 4
LRSAM1	-3.06	-1.33	0.28	3.76E-05	0.0003	leucine rich repeat and sterile alpha motif containing 1
LSM10	-3.36	-1.38	0.36	4.76E-05	0.0003	LSM10, U7 small nuclear RNA associated
LSR	-3.03	-1.34	0.26	0.0001	0.0007	lipolysis stimulated lipoprotein receptor
LSS	3.31	0.32	-1.4	5.18E-06	0.0002	lanosterol synthase (2,3-oxidosqualene-lanosterol cyclase)
LTK	3.1	0.43	-1.2	1.05E-05	0.0002	leukocyte receptor tyrosine kinase
LY6G5B; CSNK2B	-3.06	-1.33	0.28	3.30E-06	0.0002	lymphocyte antigen 6 complex, locus G5B; casein kinase 2, beta polypeptide
LY6G5B; CSNK2B	-3.01	-1.31	0.28	4.19E-06	0.0002	lymphocyte antigen 6 complex, locus G5B; casein kinase 2, beta polypeptide
LYN	-3.04	-1.28	0.32	3.99E-05	0.0003	LYN proto-oncogene, Src family tyrosine kinase
LYRM1	-3.12	-1.31	0.33	0.0002	0.0008	LYR motif containing 1
LYRM2	-3.11	-1.31	0.32	4.29E-05	0.0003	LYR motif containing 2
LZTS1-AS1	-3.3	-1.28	0.44	8.98E-06	0.0002	LZTS1 antisense RNA 1
M1AP	-3.06	-1.33	0.28	9.35E-05	0.0005	meiosis 1 associated protein
MAB21L2	3.27	0.39	-1.32	4.78E-05	0.0003	mab-21-like 2 (C. elegans)
MAFF	3.42	0.39	-1.38	3.80E-06	0.0002	v-maf avian musculoaponeurotic fibrosarcoma oncogene homolog F

MAFG	3.41	0.48	-1.29	2.11E-05	0.0002	v-maf avian musculoaponeurotic fibrosarcoma oncogene homolog G
MAFG	-3.15	-1.36	0.29	3.40E-05	0.0003	v-maf avian musculoaponeurotic fibrosarcoma oncogene homolog G
MAGEA6; MAGEA2I	-3.01	-1.31	0.28	4.90E-05	0.0003	MAGE family member A2; MAGE family member A6; MAGE family member A2B; MAGE family member A3
MAGEA4	3.06	0.3	-1.31	2.02E-05	0.0002	MAGE family member A4
MAGEA6; MAGEA3	-3.01	-1.31	0.28	4.52E-05	0.0003	MAGE family member A6; MAGE family member A3
MAGEB17	-3.23	-1.41	0.28	3.44E-05	0.0003	MAGE family member B17
MAGEB5	3.26	0.32	-1.38	3.72E-05	0.0003	MAGE family member B5
MAGI2	-3.16	-1.38	0.28	5.45E-05	0.0004	membrane associated guanylate kinase, WW and PDZ domain containing 2
MAGIX	-3.26	-1.36	0.34	2.48E-05	0.0002	MAGI family member, X-linked
MALSU1	-3.36	-1.38	0.36	6.92E-05	0.0004	mitochondrial assembly of ribosomal large subunit 1
MAMSTR	-3.15	-1.31	0.34	4.74E-06	0.0002	MEF2 activating motif and SAP domain containing transcriptional regulator
MAN2C1	-3.16	-1.38	0.28	8.35E-05	0.0005	mannosidase, alpha, class 2C, member 1
MAP2K4	-3.05	-1.38	0.23	0.0001	0.0007	mitogen-activated protein kinase kinase 4
MAPK1IP1L	-3.02	-1.37	0.22	4.88E-06	0.0002	mitogen-activated protein kinase 1 interacting protein 1-like
MAPRE1	-3.03	-1.36	0.24	0.0001	0.0006	microtubule-associated protein, RP/EB family, member 1
MAPT-AS1	3.28	0.39	-1.32	5.37E-06	0.0002	MAPT antisense RNA 1
MARC2	3.31	0.32	-1.4	4.58E-05	0.0003	mitochondrial amidoxime reducing component 2
MARVELD3	3.01	0.3	-1.29	4.42E-06	0.0002	MARVEL domain containing 3
MAS1	3.26	0.32	-1.38	3.43E-06	0.0002	MAS1 proto-oncogene, G protein-coupled receptor
MAST2	3.42	0.39	-1.38	2.31E-06	0.0002	microtubule associated serine/threonine kinase 2
MAT2A	-3.39	-1.41	0.35	2.78E-05	0.0003	methionine adenosyltransferase II, alpha
MATN2	-3.22	-1.36	0.32	1.98E-05	0.0002	matrilin 2
MATN3	-3.2	-1.31	0.36	3.95E-05	0.0003	matrilin 3
MATN4	3.25	0.32	-1.38	3.27E-06	0.0002	matrilin 4
MAU2	3.1	0.43	-1.2	3.23E-05	0.0003	MAU2 sister chromatid cohesion factor
MBD3L5; MBD3L2	-3.02	-1.36	0.23	9.36E-06	0.0002	methyl-CpG binding domain protein 3-like 5; methyl-CpG binding domain protein 3-like 2
MBD3L5; MBD3L2	-3.02	-1.36	0.23	1.27E-05	0.0002	methyl-CpG binding domain protein 3-like 5; methyl-CpG binding domain protein 3-like 2
MBL2	3.25	0.32	-1.38	4.50E-06	0.0002	mannose-binding lectin (protein C) 2, soluble
MC1R	3.3	0.28	-1.44	3.91E-05	0.0003	melanocortin 1 receptor (alpha melanocyte stimulating hormone receptor)
MC3R	3.3	0.32	-1.4	2.57E-06	0.0002	melanocortin 3 receptor
MC4R	-3.37	-1.35	0.4	0.0001	0.0006	melanocortin 4 receptor
MCAT	3.46	0.41	-1.38	6.40E-06	0.0002	malonyl-CoA-acyl carrier protein transacylase
MCCD1	-3.2	-1.31	0.36	1.51E-05	0.0002	mitochondrial coiled-coil domain 1
MCCD1	-3.2	-1.31	0.36	1.51E-05	0.0002	mitochondrial coiled-coil domain 1
MCCD1	-3.22	-1.32	0.36	4.07E-05	0.0003	mitochondrial coiled-coil domain 1
MCM3	-3.06	-1.31	0.3	6.11E-06	0.0002	minichromosome maintenance complex component 3
MCM4	3.37	0.32	-1.43	2.31E-05	0.0002	minichromosome maintenance complex component 4
MCMBP	-3.01	-1.31	0.28	1.28E-05	0.0002	minichromosome maintenance complex binding protein

MED11	-3.21	-1.38	0.3	3.80E-05	0.0003	mediator complex subunit 11
MED19	-3.23	-1.41	0.28	1.54E-05	0.0002	mediator complex subunit 19
MEGF8	-3.32	-1.38	0.35	4.93E-05	0.0003	multiple EGF-like-domains 8
METTL6	-3.06	-1.31	0.3	4.41E-05	0.0003	methyltransferase like 6
MEX3B	3.41	0.32	-1.44	3.34E-05	0.0003	mex-3 RNA binding family member B
MFSD14B	-3.16	-1.38	0.28	2.29E-05	0.0002	major facilitator superfamily domain containing 14B
MFSD5	3.59	0.44	-1.4	1.61E-05	0.0002	major facilitator superfamily domain containing 5
MGAT1	-3.15	-1.33	0.32	5.81E-05	0.0004	mannosyl (alpha-1,3-)-glycoprotein beta-1,2-N-acetylglucosaminyltransferase
MGAT3	-3.01	-1.31	0.28	0.0002	0.0008	mannosyl (beta-1,4-)-glycoprotein beta-1,4-N-acetylglucosaminyltransferase
MGAT4EP	3.43	0.4	-1.38	1.80E-05	0.0002	MGAT4 family, member E, pseudogene
MGLL	-3.15	-1.31	0.34	1.37E-05	0.0002	monoglyceride lipase
MICALL1	3.28	0.3	-1.41	1.34E-05	0.0002	MICAL-like 1
MIF	3.3	0.32	-1.4	5.02E-05	0.0003	macrophage migration inhibitory factor (glycosylation-inhibiting factor)
MILR1	-3.04	-1.28	0.32	2.88E-05	0.0003	mast cell immunoglobulin-like receptor 1
MIR103A1	-3.26	-1.38	0.32	8.56E-05	0.0005	microRNA 103a-1
MIR1-1	-3.45	-1.48	0.31	0.0001	0.0006	microRNA 1-1
MIR125B1	3.3	0.52	-1.2	4.53E-05	0.0003	microRNA 125b-1
MIR125B2	-3.01	-1.31	0.28	2.80E-05	0.0003	microRNA 125b-2
MIR135A2	-3.01	-1.31	0.28	5.26E-06	0.0002	microRNA 135a-2
MIR141	-3.26	-1.38	0.32	0.0001	0.0007	microRNA 141
MIR142	3.3	0.28	-1.44	9.49E-06	0.0002	microRNA 142
MIR145; CARMN	-3.11	-1.31	0.32	5.13E-06	0.0002	microRNA 145; cardiac mesoderm enhancer-associated non-coding RNA
MIR150	-3.16	-1.38	0.28	1.06E-05	0.0002	microRNA 150
MIR154	-3.02	-1.36	0.23	6.32E-05	0.0004	microRNA 154
195; MIR497HG; MI	3.53	0.39	-1.43	5.11E-05	0.0004	microRNA 195; mir-497-195 cluster host gene; microRNA 497
MIR196A2	3.09	0.43	-1.2	2.26E-05	0.0002	microRNA 196a-2
MIR200B	-3.01	-1.31	0.28	8.41E-05	0.0005	microRNA 200b
MIR206	-3.01	-1.31	0.28	0.0002	0.0008	microRNA 206
MIR224; GABRE	-3.23	-1.41	0.28	3.29E-05	0.0003	microRNA 224; gamma-aminobutyric acid (GABA) A receptor, epsilon
MIR23A	3.61	0.47	-1.38	6.85E-06	0.0002	microRNA 23a
MIR26A1	-3.21	-1.36	0.32	1.10E-05	0.0002	microRNA 26a-1
MIR299	-3.03	-1.43	0.17	3.87E-05	0.0003	microRNA 299
MIR339	3.2	0.39	-1.29	5.68E-06	0.0002	microRNA 339
MIR92A2	3.26	0.32	-1.38	4.05E-06	0.0002	microRNA 92a-2
MIR93	-3.03	-1.36	0.23	5.43E-05	0.0004	microRNA 93
MIR98	-3.12	-1.36	0.28	2.64E-05	0.0003	microRNA 98
MIS18A	-3.15	-1.33	0.32	0.0001	0.0007	MIS18 kinetochore protein A
MIXL1	3.12	0.26	-1.38	7.81E-06	0.0002	Mix paired-like homeobox

MKRN9P	-3.1	-1.33	0.3	2.54E-05	0.0003	makorin ring finger protein 9, pseudogene
MLLT11	-3.2	-1.31	0.37	2.03E-05	0.0002	myeloid/lymphoid or mixed-lineage leukemia; translocated to, 11
MMAB	-3.01	-1.28	0.31	1.17E-05	0.0002	methylmalonic aciduria (cobalamin deficiency) cblB type
MMP1	3.26	0.28	-1.43	4.38E-06	0.0002	matrix metallopeptidase 1
MMP15	-3.01	-1.31	0.28	2.76E-05	0.0003	matrix metallopeptidase 15 (membrane-inserted)
MMP16	-3.07	-1.36	0.25	2.64E-05	0.0003	matrix metallopeptidase 16 (membrane-inserted)
MNT	3.19	0.29	-1.38	9.99E-06	0.0002	MAX network transcriptional repressor
MOB3C; MKNK1	-3.01	-1.28	0.31	2.81E-05	0.0003	MOB kinase activator 3C; MAP kinase interacting serine/threonine kinase 1
MON1A	3.03	0.4	-1.2	2.40E-05	0.0002	MON1 secretory trafficking family member A
MORC4	3.06	0.32	-1.29	1.62E-05	0.0002	MORC family CW-type zinc finger 4
MOV10	-3.2	-1.31	0.36	4.32E-05	0.0003	Mov10 RISC complex RNA helicase
MPC1L	-3.01	-1.31	0.28	6.87E-05	0.0004	mitochondrial pyruvate carrier 1-like
MPDU1	3.06	0.32	-1.29	1.61E-05	0.0002	mannose-P-dolichol utilization defect 1
MPND	3.26	0.32	-1.38	1.13E-05	0.0002	MPN domain containing
MPRIP	-3.16	-1.31	0.35	4.40E-05	0.0003	myosin phosphatase Rho interacting protein
MRAP	-3.01	-1.31	0.28	5.26E-06	0.0002	melanocortin 2 receptor accessory protein
MROH6	-3.58	-1.46	0.38	1.65E-05	0.0002	maestro heat-like repeat family member 6
MRPL16	3.44	0.4	-1.38	3.00E-06	0.0002	mitochondrial ribosomal protein L16
MRPL2	-3.06	-1.28	0.34	4.37E-06	0.0002	mitochondrial ribosomal protein L2
MRPL20	3.48	0.38	-1.42	1.12E-05	0.0002	mitochondrial ribosomal protein L20
MRPL20	3.48	0.38	-1.42	1.12E-05	0.0002	mitochondrial ribosomal protein L20
MRPL32	-3.19	-1.33	0.34	2.79E-05	0.0003	mitochondrial ribosomal protein L32
MRPL41	-3.07	-1.3	0.32	8.70E-06	0.0002	mitochondrial ribosomal protein L41
MRPL47	3.44	0.32	-1.46	3.45E-06	0.0002	mitochondrial ribosomal protein L47
MRPL9	-3.02	-1.3	0.3	7.74E-05	0.0005	mitochondrial ribosomal protein L9
MRPS16	-3.11	-1.31	0.32	2.22E-05	0.0002	mitochondrial ribosomal protein S16
MRPS17; GBAS	3.3	0.28	-1.44	3.90E-06	0.0002	mitochondrial ribosomal protein S17; glioblastoma amplified sequence
MRPS27	-3.08	-1.31	0.31	3.90E-06	0.0002	mitochondrial ribosomal protein S27
MRPS34	-3	-1.31	0.28	1.70E-05	0.0002	mitochondrial ribosomal protein S34
MRPS7	-3.11	-1.28	0.36	1.99E-05	0.0002	mitochondrial ribosomal protein S7
MSANTD2	3.41	0.37	-1.4	3.54E-05	0.0003	Myb/SANT-like DNA-binding domain containing 2
MSRB3	3.48	0.4	-1.4	4.82E-06	0.0002	methionine sulfoxide reductase B3
MSTO1	-3.24	-1.33	0.36	0.0001	0.0007	misato 1, mitochondrial distribution and morphology regulator
MSX2	3.11	0.17	-1.47	1.32E-05	0.0002	msh homeobox 2
MT1P3	3.56	0.43	-1.4	2.47E-06	0.0002	metallothionein 1 pseudogene 3
MTHFD2	3.56	0.43	-1.4	4.46E-05	0.0003	methylenetetrahydrofolate dehydrogenase (NADP+ dependent) 2, methenyltetrahydrofolate cyclohydrolase
MTMR12	-3.1	-1.36	0.28	1.61E-05	0.0002	myotubularin related protein 12
MTMR9	-3.04	-1.28	0.32	1.15E-05	0.0002	myotubularin related protein 9

MTRF1L	3.26	0.32	-1.38	4.97E-06	0.0002	mitochondrial translational release factor 1-like
MTSS1L	3.16	0.23	-1.43	9.10E-05	0.0005	metastasis suppressor 1-like
MUC19	3.14	0.36	-1.29	4.08E-05	0.0003	mucin 19, oligomeric
MUCL1	3.33	0.32	-1.41	7.21E-06	0.0002	mucin-like 1
MUL1	3.24	0.31	-1.38	7.96E-06	0.0002	mitochondrial E3 ubiquitin protein ligase 1
MVK	3.52	0.43	-1.38	1.23E-05	0.0002	mevalonate kinase
MXRA8	3.7	0.42	-1.47	0.0001	0.0007	matrix-remodelling associated 8
MYBL2	3.21	0.3	-1.38	1.96E-05	0.0002	v-myb avian myeloblastosis viral oncogene homolog-like 2
MYCN	-3.11	-1.31	0.32	1.05E-05	0.0002	v-myc avian myelocytomatosis viral oncogene neuroblastoma derived homolog
MYD88	-3.2	-1.35	0.33	3.71E-05	0.0003	myeloid differentiation primary response 88
MYO19	-3.17	-1.36	0.3	1.13E-05	0.0002	myosin XIX
MYOG	3.43	0.38	-1.4	8.42E-06	0.0002	myogenin (myogenic factor 4)
MYOM1	-3.17	-1.36	0.3	1.08E-05	0.0002	myomesin 1
MZF1	3.54	0.42	-1.4	6.98E-06	0.0002	myeloid zinc finger 1
NAA11	-3.05	-1.36	0.24	1.14E-05	0.0002	N(alpha)-acetyltransferase 11, NatA catalytic subunit
NAA40	-3.01	-1.37	0.22	7.27E-05	0.0004	N(alpha)-acetyltransferase 40, NatD catalytic subunit
NAALADL2	-3.35	-1.42	0.32	4.79E-05	0.0003	N-acetylated alpha-linked acidic dipeptidase-like 2
NABP2	-3.42	-1.41	0.36	0.0003	0.0013	nucleic acid binding protein 2
NACC1	3.35	0.33	-1.41	3.58E-05	0.0003	nucleus accumbens associated 1, BEN and BTB (POZ) domain containing
[illegible]ANOG; NANOGP[illegible]	3.29	0.28	-1.44	3.13E-06	0.0002	Nanog homeobox; Nanog homeobox pseudogene 8
NANOGP1	-3.12	-1.36	0.28	8.37E-06	0.0002	Nanog homeobox pseudogene 1
NANS	-3.12	-1.36	0.28	8.37E-06	0.0002	N-acetylneuraminic acid synthase
NARFL	3.16	0.28	-1.38	1.10E-05	0.0002	nuclear prelamin A recognition factor-like
NBEA	3.37	0.32	-1.43	1.98E-05	0.0002	neurobeachin
NBPF7	-3.06	-1.33	0.28	1.11E-05	0.0002	neuroblastoma breakpoint family, member 7
NBR1	3.21	0.39	-1.29	5.76E-06	0.0002	neighbor of BRCA1 gene 1
NBR2	-3.01	-1.31	0.28	1.21E-05	0.0002	neighbor of BRCA1 gene 2 (non-protein coding)
NCBP3	-3.22	-1.28	0.4	1.13E-05	0.0002	nuclear cap binding subunit 3
NCKAP5	3.47	0.32	-1.47	3.49E-05	0.0003	NCK-associated protein 5
NCLN	3.24	0.3	-1.4	9.42E-06	0.0002	nicalin
NCR3LG1	-3.16	-1.31	0.35	1.50E-05	0.0002	natural killer cell cytotoxicity receptor 3 ligand 1
NDNF	-3.06	-1.31	0.3	3.89E-05	0.0003	neuron-derived neurotrophic factor
NDOR1	-3.14	-1.31	0.34	0.0002	0.0008	NADPH dependent diflavin oxidoreductase 1
NDUFA4L2	3.49	0.38	-1.43	3.29E-06	0.0002	NADH dehydrogenase (ubiquinone) 1 alpha subcomplex, 4-like 2
NDUFA8	-3.01	-1.31	0.28	8.25E-06	0.0002	NADH dehydrogenase (ubiquinone) 1 alpha subcomplex, 8, 19kDa
NDUFAF2	3.1	0.43	-1.2	9.81E-05	0.0005	NADH dehydrogenase (ubiquinone) complex I, assembly factor 2
NDUFS2	-3.12	-1.36	0.28	8.13E-05	0.0005	NADH dehydrogenase (ubiquinone) Fe-S protein 2, 49kDa (NADH-coenzyme Q reductase)
NEIL2	3.58	0.46	-1.38	1.40E-05	0.0002	nei-like DNA glycosylase 2

NEURL2	3.32	0.3	-1.43	8.70E-06	0.0002	neuralized E3 ubiquitin protein ligase 2
NEURL3	-3.01	-1.36	0.23	1.45E-05	0.0002	neuralized E3 ubiquitin protein ligase 3
NEURL4; GPS2	3.21	0.3	-1.38	1.19E-05	0.0002	neuralized E3 ubiquitin protein ligase 4; G protein pathway suppressor 2
NEUROD4	-3.19	-1.32	0.35	1.30E-05	0.0002	neuronal differentiation 4
NFE2	3.37	0.32	-1.43	6.73E-06	0.0002	nuclear factor, erythroid 2
NGRN	3.61	0.45	-1.4	2.41E-06	0.0002	neugrin, neurite outgrowth associated
NIPSNAP1; THOC5	3.16	0.28	-1.38	4.87E-06	0.0002	nipsnap homolog 1 (C. elegans); THO complex 5
NIPSNAP3A	-3.23	-1.41	0.28	8.28E-05	0.0005	nipsnap homolog 3A (C. elegans)
NIPSNAP3B	3.53	0.39	-1.43	7.05E-06	0.0002	nipsnap homolog 3B (C. elegans)
NIT1	3.26	0.41	-1.29	4.89E-05	0.0003	nitrilase 1
NKTR	3.63	0.43	-1.43	4.75E-06	0.0002	natural killer cell triggering receptor
NKX2-2	-3.04	-1.35	0.26	8.83E-06	0.0002	NK2 homeobox 2
NKX3-1	-3.12	-1.36	0.28	3.66E-05	0.0003	NK3 homeobox 1
NLRP4	3.02	0.39	-1.2	4.41E-06	0.0002	NLR family, pyrin domain containing 4
NLRX1	3.14	0.36	-1.29	7.64E-06	0.0002	NLR family member X1
NMB	-3.01	-1.31	0.28	1.97E-05	0.0002	neuromedin B
NME4	-3.28	-1.31	0.41	2.26E-05	0.0002	NME/NM23 nucleoside diphosphate kinase 4
NOB1	-3	-1.32	0.26	2.31E-05	0.0002	NIN1/RPN12 binding protein 1 homolog
NOG	-3.06	-1.34	0.27	9.27E-06	0.0002	noggin
NOP10	-3.23	-1.35	0.34	5.09E-05	0.0003	NOP10 ribonucleoprotein
NOP16	-3.08	-1.31	0.31	1.27E-05	0.0002	NOP16 nucleolar protein
NOV	-3.04	-1.32	0.28	1.11E-05	0.0002	nephroblastoma overexpressed
NOVA2	3.01	0.14	-1.44	4.20E-06	0.0002	neuro-oncological ventral antigen 2
NOX4	-3.01	-1.31	0.28	4.11E-05	0.0003	NADPH oxidase 4
NOXA1	-3.01	-1.31	0.28	6.59E-06	0.0002	NADPH oxidase activator 1
NPAT	-3.16	-1.38	0.28	0.0001	0.0006	nuclear protein, ataxia-telangiectasia locus
NPM2	-3.37	-1.48	0.28	2.74E-05	0.0003	nucleophosmin/nucleoplasmin 2
NPSR1	-3.21	-1.3	0.39	2.62E-05	0.0003	neuropeptide S receptor 1
NPSR1-AS1	3.01	0.3	-1.29	9.90E-06	0.0002	NPSR1 antisense RNA 1
NPTN	-3.21	-1.37	0.31	4.20E-05	0.0003	neuroplastin
NPY1R	-3.06	-1.3	0.32	1.74E-05	0.0002	neuropeptide Y receptor Y1
NQO1	-3.17	-1.34	0.33	0.0002	0.0011	NAD(P)H dehydrogenase, quinone 1
NR2E1	3.08	0.31	-1.31	1.53E-05	0.0002	nuclear receptor subfamily 2, group E, member 1
NR2F1	-3.1	-1.3	0.34	3.15E-05	0.0003	nuclear receptor subfamily 2, group F, member 1
NR5A1	3.16	0.34	-1.31	1.39E-05	0.0002	nuclear receptor subfamily 5, group A, member 1
NRARP	3.25	0.32	-1.38	1.00E-05	0.0002	NOTCH-regulated ankyrin repeat protein
NRSN1	-3.01	-1.3	0.29	9.47E-06	0.0002	neurensin 1
NRSN2	3.15	0.18	-1.47	9.98E-06	0.0002	neurensin 2

NSMCE1	-3.03	-1.32	0.28	0.0002	0.0008	NSE1 homolog, SMC5-SMC6 complex component
NSUN7	-3.12	-1.36	0.28	3.53E-06	0.0002	NOP2/Sun domain family, member 7
NT5M	-3.14	-1.31	0.34	1.83E-05	0.0002	5,3-nucleotidase, mitochondrial
NUDT11	-3.12	-1.36	0.28	8.72E-05	0.0005	nudix hydrolase 11
NUDT9	3.22	0.31	-1.38	9.55E-06	0.0002	nudix hydrolase 9
NUMBL	3.06	0.41	-1.2	1.08E-05	0.0002	numb homolog (Drosophila)-like
NUP54	3.16	0.28	-1.38	1.03E-05	0.0002	nucleoporin 54kDa
NUPR2	-3.02	-1.31	0.29	1.96E-05	0.0002	nuclear protein 2, transcriptional regulator
NWD1	3.41	0.39	-1.38	2.33E-06	0.0002	NACHT and WD repeat domain containing 1
NXPE1	3.2	0.39	-1.29	5.70E-06	0.0002	neurexophilin and PC-esterase domain family, member 1
NXPE4	3.26	0.32	-1.38	9.34E-06	0.0002	neurexophilin and PC-esterase domain family, member 4
OAZ1; SPPL2B	3.3	0.28	-1.44	7.62E-06	0.0002	ornithine decarboxylase antizyme 1; signal peptide peptidase like 2B
OBP2A	-3.53	-1.38	0.44	1.20E-05	0.0002	odorant binding protein 2A
OCEL1	-3.05	-1.36	0.25	3.92E-05	0.0003	occludin/ELL domain containing 1
OCIAD1	3.37	0.32	-1.43	6.59E-06	0.0002	OCIA domain containing 1
OGDHL	3.02	0.39	-1.2	1.95E-05	0.0002	oxoglutarate dehydrogenase-like
OLFM4	-3	-1.37	0.21	1.58E-05	0.0002	olfactomedin 4
OLFML2B	3.17	0.28	-1.39	4.83E-05	0.0003	olfactomedin like 2B
OPALIN	-3.06	-1.33	0.28	7.01E-06	0.0002	oligodendrocytic myelin paranodal and inner loop protein
OPN1SW	-3.31	-1.36	0.36	3.26E-06	0.0002	opsin 1 (cone pigments), short-wave-sensitive
OR10A2	3.37	0.32	-1.43	3.98E-06	0.0002	olfactory receptor, family 10, subfamily A, member 2
OR10G4	-3.41	-1.47	0.3	8.64E-05	0.0005	olfactory receptor, family 10, subfamily G, member 4
OR10G8	-3.16	-1.38	0.28	3.36E-05	0.0003	olfactory receptor, family 10, subfamily G, member 8
OR13A1	-3.05	-1.3	0.31	1.81E-05	0.0002	olfactory receptor, family 13, subfamily A, member 1
OR13C4	3.37	0.32	-1.43	0.0001	0.0007	olfactory receptor, family 13, subfamily C, member 4
OR13C5	-3.33	-1.46	0.28	0.0002	0.0010	olfactory receptor, family 13, subfamily C, member 5
OR13C8	-3.06	-1.33	0.28	1.04E-05	0.0002	olfactory receptor, family 13, subfamily C, member 8
OR13C9	-3.29	-1.35	0.37	2.48E-05	0.0002	olfactory receptor, family 13, subfamily C, member 9
OR1E3	-3.03	-1.32	0.28	9.01E-05	0.0005	olfactory receptor, family 1, subfamily E, member 3 (gene/pseudogene)
OR1L4	3.26	0.32	-1.38	0.0002	0.0008	olfactory receptor, family 1, subfamily L, member 4
OR2A14	-3.12	-1.3	0.34	0.0002	0.0008	olfactory receptor, family 2, subfamily A, member 14
OR2B11	-3.16	-1.38	0.28	1.09E-05	0.0002	olfactory receptor, family 2, subfamily B, member 11
OR2B7P	-3.05	-1.38	0.23	1.40E-05	0.0002	olfactory receptor, family 2, subfamily B, member 7 pseudogene
OR2B8P	-3.01	-1.31	0.28	6.48E-05	0.0004	olfactory receptor, family 2, subfamily B, member 8 pseudogene
OR2D3	-3.06	-1.33	0.28	3.99E-06	0.0002	olfactory receptor, family 2, subfamily D, member 3
OR2G3	-3.04	-1.38	0.22	0.0002	0.0009	olfactory receptor, family 2, subfamily G, member 3
OR2H2; OR2H1	-3.16	-1.38	0.28	0.0002	0.0010	olfactory receptor, family 2, subfamily H, member 2; olfactory receptor, family 2, subfamily H, member 1
OR2T11	-3.36	-1.38	0.36	0.0001	0.0006	olfactory receptor, family 2, subfamily T, member 11 (gene/pseudogene)

OR2T2	-3.06	-1.33	0.28	2.56E-05	0.0003	olfactory receptor, family 2, subfamily T, member 2
OR2T35; OR2T2	-3.06	-1.33	0.28	2.56E-05	0.0003	olfactory receptor, family 2, subfamily T, member 35; olfactory receptor, family 2, subfamily T, member 2
OR2W1	-3.16	-1.38	0.28	4.09E-05	0.0003	olfactory receptor, family 2, subfamily W, member 1
OR2W1	-3.16	-1.38	0.28	4.09E-05	0.0003	olfactory receptor, family 2, subfamily W, member 1
OR2W1	-3.16	-1.38	0.28	4.09E-05	0.0003	olfactory receptor, family 2, subfamily W, member 1
OR4A21P	-3.12	-1.36	0.28	7.16E-05	0.0004	olfactory receptor, family 4, subfamily A, member 21 pseudogene
OR4B1	-3.12	-1.36	0.28	0.0002	0.0008	olfactory receptor, family 4, subfamily B, member 1
OR4D10	-3.12	-1.31	0.33	6.49E-05	0.0004	olfactory receptor, family 4, subfamily D, member 10
OR4D5	3.42	0.39	-1.38	1.88E-05	0.0002	olfactory receptor, family 4, subfamily D, member 5
OR51A8P	-3.1	-1.36	0.27	1.78E-05	0.0002	olfactory receptor, family 51, subfamily A, member 8 pseudogene
OR51J1	-3.26	-1.38	0.32	0.0001	0.0006	olfactory receptor, family 51, subfamily J, member 1 (gene/pseudogene)
OR52B3P	-3.34	-1.36	0.38	1.40E-05	0.0002	olfactory receptor, family 52, subfamily B, member 3 pseudogene
OR52B4	-3.06	-1.3	0.32	1.81E-05	0.0002	olfactory receptor, family 52, subfamily B, member 4 (gene/pseudogene)
OR52D1	-3.06	-1.33	0.28	6.13E-06	0.0002	olfactory receptor, family 52, subfamily D, member 1
OR52E4	-3.26	-1.38	0.32	0.0002	0.0011	olfactory receptor, family 52, subfamily E, member 4
OR52K1	-3.12	-1.31	0.33	0.0002	0.0008	olfactory receptor, family 52, subfamily K, member 1
OR52Z1	3.08	0.22	-1.4	1.60E-05	0.0002	olfactory receptor, family 52, subfamily Z, member 1 (gene/pseudogene)
OR56A3	-3.12	-1.38	0.26	2.63E-05	0.0003	olfactory receptor, family 56, subfamily A, member 3
OR5BB1P	3.26	0.32	-1.38	1.05E-05	0.0002	olfactory receptor, family 5, subfamily BB, member 1 pseudogene
OR5E1P	3.37	0.32	-1.43	1.97E-05	0.0002	olfactory receptor, family 5, subfamily E, member 1 pseudogene
OR5K3	-3.31	-1.36	0.36	4.71E-05	0.0003	olfactory receptor, family 5, subfamily K, member 3
OR5R1	3.26	0.32	-1.38	9.34E-06	0.0002	olfactory receptor, family 5, subfamily R, member 1 (gene/pseudogene)
OR6K6	-3.01	-1.31	0.28	2.91E-05	0.0003	olfactory receptor, family 6, subfamily K, member 6
OR6P1	3.16	0.23	-1.43	4.46E-06	0.0002	olfactory receptor, family 6, subfamily P, member 1
OR7D4	-3.1	-1.36	0.27	4.77E-05	0.0003	olfactory receptor, family 7, subfamily D, member 4
OR7E14P; OR7E145	3.16	0.23	-1.43	4.46E-06	0.0002	olfactory receptor, family 7, subfamily E, member 14 pseudogene; olfactory receptor, family 7, subfamily E, member 145 pseudogene
OR7E162P	-3.15	-1.32	0.33	3.27E-05	0.0003	olfactory receptor, family 7, subfamily E, member 162 pseudogene
OR7G1	-3.28	-1.46	0.25	3.82E-05	0.0003	olfactory receptor, family 7, subfamily G, member 1
OR8A1	3.04	0.14	-1.47	1.24E-05	0.0002	olfactory receptor, family 8, subfamily A, member 1
OR8B8	3.4	0.38	-1.38	5.12E-06	0.0002	olfactory receptor, family 8, subfamily B, member 8
OR8K1	-3.24	-1.33	0.36	1.93E-05	0.0002	olfactory receptor, family 8, subfamily K, member 1
OR8K3	-3.41	-1.36	0.4	7.37E-06	0.0002	olfactory receptor, family 8, subfamily K, member 3 (gene/pseudogene)
ORC1	3.26	0.32	-1.38	9.64E-05	0.0005	origin recognition complex subunit 1
OTOP2	3.2	0.23	-1.44	7.19E-06	0.0002	otopetrin 2
OTUD3	3.01	0.39	-1.2	8.10E-05	0.0005	OTU deubiquitinase 3
OVOL1	3.09	0.23	-1.4	1.09E-05	0.0002	ovo-like zinc finger 1
OXCT2P1; OXCT2	3.46	0.41	-1.38	3.36E-06	0.0002	3-oxoacid CoA-transferase 2 pseudogene 1; 3-oxoacid CoA-transferase 2
OXCT2P1; OXCT2	3.46	0.41	-1.38	3.36E-06	0.0002	3-oxoacid CoA-transferase 2 pseudogene 1; 3-oxoacid CoA-transferase 2

P3H1	3.2	0.28	-1.4	3.27E-05	0.0003	prolyl 3-hydroxylase 1
PABPC3	-3.04	-1.28	0.32	2.50E-05	0.0002	poly(A) binding protein, cytoplasmic 3
PACSIN2	3.07	0.24	-1.38	9.02E-06	0.0002	protein kinase C and casein kinase substrate in neurons 2
PAEP	3.37	0.32	-1.43	8.46E-06	0.0002	progestagen-associated endometrial protein
PAF1	-3.12	-1.36	0.28	2.52E-05	0.0003	PAF1 homolog, Paf1/RNA polymerase II complex component
PAFAH1B2	3.03	0.3	-1.3	2.39E-05	0.0002	platelet-activating factor acetylhydrolase 1b, catalytic subunit 2 (30kDa)
PAG1	-3.07	-1.34	0.28	1.18E-05	0.0002	phosphoprotein membrane anchor with glycosphingolipid microdomains 1
PAIP1	3.37	0.32	-1.43	3.11E-06	0.0002	poly(A) binding protein interacting protein 1
PAK3	-3.16	-1.38	0.28	0.0002	0.0007	p21 protein (Cdc42/Rac)-activated kinase 3
PALLD	3.16	0.28	-1.38	2.96E-06	0.0002	palladin, cytoskeletal associated protein
PAMR1	-3.32	-1.41	0.32	2.89E-05	0.0003	peptidase domain containing associated with muscle regeneration 1
PAN2; CNPY2	3.26	0.32	-1.38	2.75E-05	0.0003	PAN2 poly(A) specific ribonuclease subunit; canopy FGF signaling regulator 2
PAPD7	3.37	0.32	-1.43	3.00E-06	0.0002	PAP associated domain containing 7
PAQR5	3.08	0.19	-1.44	3.21E-06	0.0002	progestin and adipoQ receptor family member V
PAQR9	-3.25	-1.31	0.39	5.13E-06	0.0002	progestin and adipoQ receptor family member IX
PARP2	3.37	0.32	-1.43	3.73E-05	0.0003	poly(ADP-ribose) polymerase 2
PAX6	3.74	0.43	-1.47	3.10E-05	0.0003	paired box 6
PCDH17	-3	-1.33	0.25	2.50E-05	0.0002	protocadherin 17
PCDHB11	3.44	0.32	-1.46	2.69E-05	0.0003	protocadherin beta 11
PCDHB3	3.47	0.32	-1.47	0.0002	0.0008	protocadherin beta 3
PCED1A	3.26	0.32	-1.38	9.94E-06	0.0002	PC-esterase domain containing 1A
PCGF7P	-3.16	-1.38	0.28	5.64E-05	0.0004	polycomb group ring finger 7 pseudogene
PCNP	-3.29	-1.4	0.32	1.48E-05	0.0002	PEST proteolytic signal containing nuclear protein
PCNXL3	-3.12	-1.36	0.28	0.0001	0.0006	pecanex-like 3 (Drosophila)
PDCD2L	3.39	0.32	-1.44	3.30E-06	0.0002	programmed cell death 2-like
PDCD7	-3.03	-1.3	0.3	0.0001	0.0006	programmed cell death 7
PDCL	-3.16	-1.38	0.28	3.20E-05	0.0003	phosducin like
PDDC1	3.55	0.41	-1.41	5.40E-05	0.0004	Parkinson disease 7 domain containing 1
PDE1C	-3.06	-1.33	0.28	1.80E-05	0.0002	phosphodiesterase 1C, calmodulin-dependent 70kDa
PDE2A	3.26	0.32	-1.38	2.68E-06	0.0002	phosphodiesterase 2A, cGMP-stimulated
PDE4DIP; PFN1P2	3.26	0.32	-1.38	9.94E-06	0.0002	phosphodiesterase 4D interacting protein; profilin 1 pseudogene 2
PDGFRL	-3.35	-1.43	0.31	8.10E-06	0.0002	platelet-derived growth factor receptor-like
PDHX	-3.08	-1.38	0.24	2.77E-05	0.0003	pyruvate dehydrogenase complex, component X
PDLIM5	-3.26	-1.38	0.32	2.29E-05	0.0002	PDZ and LIM domain 5
PEBP1	3.67	0.43	-1.44	6.03E-05	0.0004	phosphatidylethanolamine binding protein 1
PEX11A	-3.17	-1.36	0.3	9.64E-06	0.0002	peroxisomal biogenesis factor 11 alpha
PEX14	-3.21	-1.3	0.38	1.00E-05	0.0002	peroxisomal biogenesis factor 14
PEX19	-3.04	-1.28	0.32	2.05E-05	0.0002	peroxisomal biogenesis factor 19

PFKFB4	-3.16	-1.38	0.28	4.84E-06	0.0002	6-phosphofructo-2-kinase/fructose-2,6-biphosphatase 4
PFKM	-3.16	-1.31	0.35	0.0001	0.0006	phosphofructokinase, muscle
PGAM2	-3.03	-1.3	0.3	2.92E-05	0.0003	phosphoglycerate mutase 2 (muscle)
PGPEP1L	3.28	0.32	-1.39	4.87E-05	0.0003	pyroglutamyl-peptidase I-like
PHF2	-3.03	-1.38	0.22	2.03E-05	0.0002	PHD finger protein 2
PHLDA1	-3.04	-1.35	0.26	0.0001	0.0006	pleckstrin homology-like domain, family A, member 1
PHLPP1	-3.01	-1.31	0.28	1.62E-05	0.0002	PH domain and leucine rich repeat protein phosphatase 1
PHOSPHO1	-3.06	-1.33	0.28	9.34E-05	0.0005	phosphatase, orphan 1
PHOX2A	3.26	0.32	-1.38	8.28E-06	0.0002	paired-like homeobox 2a
PHYH	3.39	0.35	-1.41	1.88E-05	0.0002	phytanoyl-CoA 2-hydroxylase
PI4KAP1	-3.24	-1.33	0.36	5.03E-05	0.0003	phosphatidylinositol 4-kinase, catalytic, alpha pseudogene 1
PI4KAP1; PI4KAP2	-3.04	-1.28	0.32	1.28E-05	0.0002	phosphatidylinositol 4-kinase, catalytic, alpha pseudogene 1; phosphatidylinositol 4-kinase, catalytic, alpha pseudogene 2
PIAS4	-3.03	-1.3	0.31	5.06E-05	0.0003	protein inhibitor of activated STAT 4
PIGV	3.06	0.41	-1.2	3.98E-06	0.0002	phosphatidylinositol glycan anchor biosynthesis class V
PIK3CB	3.2	0.3	-1.38	1.12E-05	0.0002	phosphatidylinositol-4,5-bisphosphate 3-kinase, catalytic subunit beta
RIG2P-PILRB; STAG	3.05	0.32	-1.29	3.70E-06	0.0002	paired immunoglobin-like type 2 receptor beta; STAG3L5P-PVRIG2P-PILRB readthrough; stromal antigen 3-like 5 pseudogene; poliovirus receptor related immunoglobulin domain containing 2, pseudogene; paired immunoglobin-like type 2 receptor alpha
PINX1	3.2	0.25	-1.43	3.42E-06	0.0002	PIN2/TERF1 interacting, telomerase inhibitor 1
PIP5K1A	-3.13	-1.28	0.36	0.0002	0.0008	phosphatidylinositol-4-phosphate 5-kinase, type I, alpha
PIP5KL1	-3.18	-1.28	0.39	0.0002	0.0010	phosphatidylinositol-4-phosphate 5-kinase-like 1
PIR	-3.26	-1.38	0.32	8.66E-05	0.0005	pirin
PKD1	-3.2	-1.31	0.36	0.0003	0.0011	polycystic kidney disease 1 (autosomal dominant)
PKDCC	-3.04	-1.28	0.32	1.32E-05	0.0002	protein kinase domain containing, cytoplasmic
PLA2G2E	-3.09	-1.35	0.28	1.33E-05	0.0002	phospholipase A2, group IIE
PLA2G4D	3.53	0.39	-1.43	1.21E-05	0.0002	phospholipase A2, group IVD (cytosolic)
PLA2G7	-3.01	-1.31	0.28	4.44E-06	0.0002	phospholipase A2, group VII (platelet-activating factor acetylhydrolase, plasma)
PLBD1	-3.16	-1.36	0.3	0.0002	0.0009	phospholipase B domain containing 1
PLEKHG4	-3.14	-1.33	0.32	4.57E-06	0.0002	pleckstrin homology domain containing, family G (with RhoGef domain) member 4
PLPPR5	-3.09	-1.35	0.28	1.64E-05	0.0002	phospholipid phosphatase related 5
PLVAP	-3.2	-1.31	0.36	4.31E-06	0.0002	plasmalemma vesicle associated protein
PMS2P7; PMS2P10	-3.04	-1.31	0.3	3.54E-05	0.0003	PMS1 homolog 2, mismatch repair system component pseudogene 2; PMS1 homolog 2, mismatch repair system component pseudogene 7; PMS1 homolog 2, mismatch repair system component pseudogene 10; PMS1 homolog 2, mismatch repair system component pseudogene 6
2P7; PMS2P10; PM	-3.08	-1.32	0.3	5.42E-06	0.0002	PMS1 homolog 2, mismatch repair system component pseudogene 2; PMS1 homolog 2, mismatch repair system component pseudogene 7; PMS1 homolog 2, mismatch repair system component pseudogene 10; PMS1 homolog 2, mismatch repair system component pseudogene 6; PMS1 homolog 2, mismatch repair system component pseudogene 5
PNMA1	-3.13	-1.28	0.36	2.63E-05	0.0003	paraneoplastic Ma antigen 1
POLK	-3.16	-1.38	0.28	2.09E-05	0.0002	polymerase (DNA directed) kappa
POLN; HAUS3	3.25	0.4	-1.3	3.64E-05	0.0003	polymerase (DNA directed) nu; HAUS augmin like complex subunit 3
POLR1B	-3.23	-1.41	0.28	6.30E-06	0.0002	polymerase (RNA) I polypeptide B
POLR1E	3.26	0.32	-1.38	3.86E-05	0.0003	polymerase (RNA) I polypeptide E
POLR2H	-3.04	-1.28	0.32	2.82E-05	0.0003	polymerase (RNA) II (DNA directed) polypeptide H
POLR2J	3.53	0.44	-1.38	3.17E-06	0.0002	polymerase (RNA) II (DNA directed) polypeptide J, 13.3kDa

R2J4; POLR2J3; POL	-3.02	-1.31	0.28	5.11E-05	0.0004	polymerase (RNA) II (DNA directed) polypeptide J4, pseudogene; polymerase (RNA) II (DNA directed) polypeptide J3; polymerase (RNA) II (DNA directed) polypeptide J2
POLR2K	-3.21	-1.36	0.32	2.23E-05	0.0002	polymerase (RNA) II (DNA directed) polypeptide K, 7.0kDa
POM121L3P	3.6	0.38	-1.47	9.14E-05	0.0005	POM121 transmembrane nucleoporin-like 3, pseudogene
POMC	-3.2	-1.38	0.29	2.27E-05	0.0002	proopiomelanocortin
POMGNT1	-3.06	-1.31	0.3	1.01E-05	0.0002	protein O-linked mannose N-acetylglucosaminyltransferase 1 (beta 1,2-)
POSTN	-3.03	-1.32	0.28	4.03E-06	0.0002	periostin, osteoblast specific factor
POTEG; POTEM	-3.78	-1.48	0.44	0.0002	0.0010	POTE ankyrin domain family, member G; POTE ankyrin domain family, member M
POU1F1	3.49	0.41	-1.39	2.61E-06	0.0002	POU class 1 homeobox 1
POU3F2	-3.09	-1.28	0.35	3.62E-05	0.0003	POU class 3 homeobox 2
POU5F1B	3.41	0.32	-1.44	1.73E-05	0.0002	POU class 5 homeobox 1B
POU5F1P4	3.57	0.39	-1.44	1.73E-05	0.0002	POU class 5 homeobox 1 pseudogene 4
POU6F2	3.36	0.36	-1.39	2.13E-05	0.0002	POU class 6 homeobox 2
PPARA	3.23	0.4	-1.29	2.46E-05	0.0002	peroxisome proliferator-activated receptor alpha
PPFIA3	3.38	0.36	-1.4	4.69E-05	0.0003	protein tyrosine phosphatase, receptor type, f polypeptide (PTPRF), interacting protein (liprin), alpha 3
PPIAL4F; PPIAL4E	3.06	0.32	-1.29	5.41E-06	0.0002	peptidylprolyl isomerase A (cyclophilin A)-like 4F; peptidylprolyl isomerase A (cyclophilin A)-like 4E
PPIAL4F; PPIAL4E	3.06	0.32	-1.29	5.41E-06	0.0002	peptidylprolyl isomerase A (cyclophilin A)-like 4F; peptidylprolyl isomerase A (cyclophilin A)-like 4E
PPM1E	-3.13	-1.32	0.32	7.70E-06	0.0002	protein phosphatase, Mg2+/Mn2+ dependent, 1E
PPM1N	-3.36	-1.38	0.36	2.39E-05	0.0002	protein phosphatase, Mg2+/Mn2+ dependent, 1N (putative)
PPP1R14D	3.22	0.26	-1.43	9.54E-06	0.0002	protein phosphatase 1, regulatory (inhibitor) subunit 14D
PPP1R26	-3.14	-1.37	0.28	1.71E-05	0.0002	protein phosphatase 1, regulatory subunit 26
PPP1R2P3	-3.16	-1.41	0.25	5.38E-05	0.0004	protein phosphatase 1, regulatory (inhibitor) subunit 2 pseudogene 3
PPP1R32	-3.01	-1.31	0.28	1.19E-05	0.0002	protein phosphatase 1, regulatory subunit 32
PPP1R35	-3.12	-1.36	0.28	8.17E-06	0.0002	protein phosphatase 1, regulatory subunit 35
PPP1R3B	3.02	0.39	-1.2	8.32E-06	0.0002	protein phosphatase 1, regulatory subunit 3B
PPP1R7	3.11	0.21	-1.43	3.69E-05	0.0003	protein phosphatase 1, regulatory subunit 7
PPP1R9A	-3.1	-1.37	0.26	9.93E-06	0.0002	protein phosphatase 1, regulatory subunit 9A
PPY2P	-3.06	-1.35	0.26	3.43E-05	0.0003	pancreatic polypeptide 2, pseudogene
PQBP1	-3.04	-1.28	0.32	1.23E-05	0.0002	polyglutamine binding protein 1
PRAF2	-3.16	-1.4	0.26	7.15E-06	0.0002	PRA1 domain family, member 2
F19; PRAMEF18; PR	-3.17	-1.36	0.3	2.83E-05	0.0003	PRAME family member 19; PRAME family member 18; PRAME family member 22
F19; PRAMEF18; PR	-3.17	-1.36	0.3	2.83E-05	0.0003	PRAME family member 19; PRAME family member 18; PRAME family member 22
PRAMEF8; PRAMEF	3.02	0.39	-1.2	1.06E-05	0.0002	PRAME family member 8; PRAME family member 7
PRAMEF8; PRAMEF	3.02	0.39	-1.2	1.06E-05	0.0002	PRAME family member 8; PRAME family member 7
PRAMEF8; PRAMEF	3.02	0.39	-1.2	1.06E-05	0.0002	PRAME family member 8; PRAME family member 7
PRDM11	3.28	0.28	-1.44	8.65E-06	0.0002	PR domain containing 11
PRDM12	-3.13	-1.28	0.36	7.26E-05	0.0004	PR domain containing 12
PRDM2	3.41	0.32	-1.44	1.23E-05	0.0002	PR domain containing 2, with ZNF domain
PRDM7	-3.06	-1.38	0.23	0.0002	0.0008	PR domain containing 7

PRELID3A	-3.12	-1.36	0.28	1.14E-05	0.0002	PRELI domain containing 3A
PRIM1	3.41	0.32	-1.44	1.73E-05	0.0002	primase, DNA, polypeptide 1 (49kDa)
PRKAA1	-3.03	-1.31	0.29	9.33E-06	0.0002	protein kinase, AMP-activated, alpha 1 catalytic subunit
PRKAB1	-3.01	-1.31	0.28	6.75E-05	0.0004	protein kinase, AMP-activated, beta 1 non-catalytic subunit
PRKAR1A	-3.01	-1.31	0.28	1.09E-05	0.0002	protein kinase, cAMP-dependent, regulatory, type I, alpha
PRKAR1B	3.1	0.43	-1.2	0.0001	0.0007	protein kinase, cAMP-dependent, regulatory, type I, beta
PRKAR2A	-3.12	-1.35	0.29	1.23E-05	0.0002	protein kinase, cAMP-dependent, regulatory, type II, alpha
PRLR	-3.06	-1.28	0.33	1.05E-05	0.0002	prolactin receptor
PROCR	-3.11	-1.32	0.32	9.47E-05	0.0005	protein C receptor, endothelial
PROM2	-3.13	-1.28	0.36	1.74E-05	0.0002	prominin 2
PRORY	-3.13	-1.28	0.36	0.0001	0.0006	proline rich, Y-linked
PROS1	3.11	0.43	-1.2	0.0001	0.0006	protein S (alpha)
PRPF40B	-3.07	-1.38	0.24	2.62E-05	0.0003	PRP40 homolog, pre-mRNA processing factor B
PRR21	-3.01	-1.31	0.28	2.98E-05	0.0003	proline rich 21
PRR26	-3.1	-1.33	0.3	2.93E-05	0.0003	proline rich 26
PRRT1	3.1	0.25	-1.38	7.65E-05	0.0004	proline-rich transmembrane protein 1
PRRT1	3.1	0.25	-1.38	7.65E-05	0.0004	proline-rich transmembrane protein 1
PRSS12	-3.04	-1.28	0.32	3.99E-05	0.0003	protease, serine, 12 (neurotrypsin, motopsin)
PRSS33	-3.03	-1.36	0.24	3.68E-05	0.0003	protease, serine, 33
PRSS35	-3.13	-1.36	0.28	1.13E-05	0.0002	protease, serine 35
PRSS3P2; PRSS2	3.42	0.37	-1.4	2.80E-05	0.0003	protease, serine 3 pseudogene 2; protease, serine, 2 (trypsin 2)
PRSS54	3.26	0.3	-1.4	4.07E-06	0.0002	protease, serine, 54
PRSS55	-3.01	-1.31	0.28	4.12E-05	0.0003	protease, serine, 55
PRSS8	3.22	0.31	-1.38	1.60E-05	0.0002	protease, serine, 8
PSD	-3.17	-1.32	0.34	8.05E-06	0.0002	pleckstrin and Sec7 domain containing
PSD4	3.42	0.38	-1.39	1.25E-05	0.0002	pleckstrin and Sec7 domain containing 4
PSG8; PSG10P	-3.01	-1.31	0.28	3.24E-05	0.0003	pregnancy specific beta-1-glycoprotein 8; pregnancy specific beta-1-glycoprotein 10, pseudogene
PSG9	-3.01	-1.31	0.28	1.82E-05	0.0002	pregnancy specific beta-1-glycoprotein 9
PSMB10	3.04	0.4	-1.2	0.0004	0.0016	proteasome subunit beta 10
PSMB9	3.16	0.28	-1.38	7.35E-06	0.0002	proteasome subunit beta 9
PSMB9	3.16	0.28	-1.38	7.35E-06	0.0002	proteasome subunit beta 9
PSMB9	3.16	0.28	-1.38	7.35E-06	0.0002	proteasome subunit beta 9
PSMD2	-3.16	-1.38	0.28	6.72E-06	0.0002	proteasome 26S subunit, non-ATPase 2
PSMD3	-3.2	-1.31	0.36	2.30E-05	0.0002	proteasome 26S subunit, non-ATPase 3
PSMD7	3.06	0.23	-1.38	3.88E-05	0.0003	proteasome 26S subunit, non-ATPase 7
PSORS1C2	-3.01	-1.31	0.28	1.07E-05	0.0002	psoriasis susceptibility 1 candidate 2
PSPN	-3.02	-1.36	0.23	3.18E-05	0.0003	persephin
PSRC1	3.13	0.35	-1.29	4.84E-05	0.0003	proline/serine-rich coiled-coil 1

PTBP2	3.23	0.25	-1.44	3.57E-06	0.0002	polypyrimidine tract binding protein 2
PTF1A	3.19	0.47	-1.2	1.99E-05	0.0002	pancreas specific transcription factor, 1a
PTGES2	3.3	0.32	-1.4	9.55E-06	0.0002	prostaglandin E synthase 2
PTPN21	3.02	0.39	-1.2	1.99E-05	0.0002	protein tyrosine phosphatase, non-receptor type 21
PTPN3	-3.03	-1.31	0.29	1.05E-05	0.0002	protein tyrosine phosphatase, non-receptor type 3
PTPRCAP; CORO1E	3.02	0.39	-1.2	1.45E-05	0.0002	protein tyrosine phosphatase, receptor type, C-associated protein; coronin, actin binding protein, 1B
PTPRH	3.61	0.47	-1.38	1.59E-05	0.0002	protein tyrosine phosphatase, receptor type, H
PTPRU	3.09	0.34	-1.29	7.66E-06	0.0002	protein tyrosine phosphatase, receptor type, U
PTRH2	-3.03	-1.36	0.24	1.60E-05	0.0002	peptidyl-tRNA hydrolase 2
PUS1	-3.07	-1.28	0.34	2.90E-05	0.0003	pseudouridylate synthase 1
PUS10	3.3	0.32	-1.4	4.22E-06	0.0002	pseudouridylate synthase 10
PYDC2	-3.1	-1.41	0.22	7.66E-05	0.0004	pyrin domain containing 2
PZP	-3.12	-1.36	0.28	2.92E-05	0.0003	pregnancy-zone protein
QARS	-3.16	-1.38	0.28	3.36E-05	0.0003	glutaminyl-tRNA synthetase
RAB11A	3.26	0.32	-1.38	5.53E-05	0.0004	RAB11A, member RAS oncogene family
RAB11FIP2	3.26	0.32	-1.38	5.09E-06	0.0002	RAB11 family interacting protein 2 (class I)
RAB24; MXD3	3.37	0.32	-1.43	4.44E-06	0.0002	RAB24, member RAS oncogene family; MAX dimerization protein 3
RAB37	3.42	0.39	-1.38	9.19E-06	0.0002	RAB37, member RAS oncogene family
RAB3C	-3.15	-1.38	0.27	1.45E-05	0.0002	RAB3C, member RAS oncogene family
RAB42	3.47	0.38	-1.41	3.88E-05	0.0003	RAB42, member RAS oncogene family
RAB43P1	3.32	0.34	-1.39	1.46E-05	0.0002	RAB43 pseudogene 1
RAB5A	-3.04	-1.28	0.32	4.46E-05	0.0003	RAB5A, member RAS oncogene family
RAB5B	-3.15	-1.35	0.31	5.71E-05	0.0004	RAB5B, member RAS oncogene family
RAB5C	-3.16	-1.32	0.34	5.83E-05	0.0004	RAB5C, member RAS oncogene family
RAB7A	-3.27	-1.36	0.35	1.50E-05	0.0002	RAB7A, member RAS oncogene family
RABL2B	-3.06	-1.33	0.28	0.0001	0.0006	RAB, member of RAS oncogene family-like 2B
RABL2B; RABL2A	-3.18	-1.35	0.32	0.0007	0.0028	RAB, member of RAS oncogene family-like 2B; RAB, member of RAS oncogene family-like 2A
RABL3	3.13	0.26	-1.38	1.74E-05	0.0002	RAB, member of RAS oncogene family-like 3
RAD1	-3.17	-1.39	0.28	8.10E-05	0.0005	RAD1 checkpoint DNA exonuclease
RAD51C	-3.02	-1.38	0.21	4.74E-05	0.0003	RAD51 paralog C
RAD54L	3.1	0.43	-1.2	1.09E-05	0.0002	RAD54-like (S. cerevisiae)
RAET1G	3.52	0.43	-1.38	2.02E-05	0.0002	retinoic acid early transcript 1G
RANBP17	3.14	0.26	-1.39	5.65E-06	0.0002	RAN binding protein 17
RANBP3	3.25	0.38	-1.31	1.00E-05	0.0002	RAN binding protein 3
RANBP9	-3.11	-1.31	0.32	4.74E-06	0.0002	RAN binding protein 9
RAPH1	3.13	0.2	-1.44	1.02E-05	0.0002	Ras association (RalGDS/AF-6) and pleckstrin homology domains 1
RARRES2	-3.09	-1.28	0.35	7.20E-05	0.0004	retinoic acid receptor responder (tazarotene induced) 2
RASA4	-3.37	-1.48	0.28	1.94E-05	0.0002	RAS p21 protein activator 4

RASL10A	-3.08	-1.32	0.3	0.0008	0.0029	RAS-like, family 10, member A
RASSF4	-3.22	-1.41	0.27	1.01E-05	0.0002	Ras association (RalGDS/AF-6) domain family member 4
RB1	3.24	0.3	-1.4	4.13E-06	0.0002	retinoblastoma 1
BAK; RBAK-RBAKD	3.3	0.32	-1.4	9.55E-06	0.0002	RB-associated KRAB zinc finger; RBAK-RBAKDN readthrough
RBFA	3.4	0.45	-1.31	3.61E-06	0.0002	ribosome binding factor A (putative)
RBM39	3.47	0.32	-1.47	2.03E-05	0.0002	RNA binding motif protein 39
Y1B; RBMY1D; RBM	3.53	0.39	-1.43	1.97E-05	0.0002	RNA binding motif protein, Y-linked, family 1, member B; RNA binding motif protein, Y-linked, family 1, member D; RNA binding motif protein, Y-linked, family 1, member E
2FP; RBMY1D; RBM	3.42	0.39	-1.38	5.27E-06	0.0002	RNA binding motif protein, Y-linked, family 1, member B; RNA binding motif protein, Y-linked, family 2, member F pseudogene; RNA binding motif protein, Y-linked, family 1, member D; RNA binding motif protein, Y-linked, family 1, member E; RNA binding motif protein, Y-linked, family 1, member A3 pseudogene
RBPJL	-3.23	-1.31	0.38	1.08E-05	0.0002	recombination signal binding protein for immunoglobulin kappa J region-like
RCAN1	3.18	0.23	-1.44	1.04E-05	0.0002	regulator of calcineurin 1
RCAN2	-3.22	-1.28	0.4	7.59E-06	0.0002	regulator of calcineurin 2
RDM1	-3.01	-1.31	0.28	3.95E-05	0.0003	RAD52 motif containing 1
RECQL4	-3.1	-1.34	0.29	2.71E-05	0.0003	RecQ helicase-like 4
REG3A	-3.07	-1.42	0.2	2.13E-05	0.0002	regenerating islet-derived 3 alpha
RELN	-3.13	-1.41	0.23	1.13E-05	0.0002	reelin
REPS2	3.1	0.25	-1.38	3.76E-05	0.0003	RALBP1 associated Eps domain containing 2
RER1	-3.26	-1.38	0.32	0.0001	0.0006	retention in endoplasmic reticulum sorting receptor 1
RETN	3.37	0.32	-1.43	2.42E-06	0.0002	resistin
REXO1L8P	-3.03	-1.28	0.32	4.11E-06	0.0002	REX1, RNA exonuclease 1 homolog-like 8, pseudogene
REXO1L8P	-3.03	-1.28	0.32	4.11E-06	0.0002	REX1, RNA exonuclease 1 homolog-like 8, pseudogene
REXO1L8P	-3.03	-1.28	0.32	4.11E-06	0.0002	REX1, RNA exonuclease 1 homolog-like 8, pseudogene
RFFL	3.39	0.32	-1.44	3.05E-06	0.0002	ring finger and FYVE-like domain containing E3 ubiquitin protein ligase
RFK	3.02	0.22	-1.38	4.01E-05	0.0003	riboflavin kinase
RFPL1	-3.34	-1.35	0.39	2.34E-05	0.0002	ret finger protein-like 1
RFT1	3.61	0.47	-1.38	0.0001	0.0006	RFT1 homolog
RGMA	3.5	0.42	-1.38	2.16E-06	0.0002	repulsive guidance molecule family member a
RGS10	-3.43	-1.41	0.36	7.10E-06	0.0002	regulator of G-protein signaling 10
RHOB	3.06	0.41	-1.2	5.39E-06	0.0002	ras homolog family member B
RHOD	-3.04	-1.28	0.32	1.06E-05	0.0002	ras homolog family member D
RHOXF1	3.43	0.48	-1.3	1.87E-05	0.0002	Rhox homeobox family, member 1
RIOK1	3.05	0.41	-1.2	5.71E-05	0.0004	RIO kinase 1
RIPPLY1	-3.01	-1.31	0.28	5.26E-06	0.0002	ripply transcriptional repressor 1
RIPPLY2	-3.12	-1.36	0.28	5.13E-05	0.0004	ripply transcriptional repressor 2
RIT1	-3.02	-1.41	0.18	1.18E-05	0.0002	Ras-like without CAAX 1
RITA1	-3.14	-1.37	0.28	6.22E-05	0.0004	RBPJ interacting and tubulin associated 1
RLN3; IL27RA	-3.04	-1.34	0.26	1.52E-05	0.0002	relaxin 3; interleukin 27 receptor, alpha
RNASE9	-3.13	-1.33	0.31	1.73E-05	0.0002	ribonuclease, RNase A family, 9 (non-active)
C17orf49; RNASEK-	-3.16	-1.38	0.28	2.29E-05	0.0002	ribonuclease, RNase K; chromosome 17 open reading frame 49; RNASEK-C17orf49 readthrough

SEK; RNASEK-C17c	-3.31	-1.36	0.36	1.46E-05	0.0002	ribonuclease, RNase K; RNASEK-C17orf49 readthrough
RNF13	-3.09	-1.35	0.28	3.66E-06	0.0002	ring finger protein 13
RNF165	-3.04	-1.31	0.29	3.85E-05	0.0003	ring finger protein 165
RNF166	-3.18	-1.28	0.39	4.42E-05	0.0003	ring finger protein 166
RNF167	3.02	0.39	-1.2	3.79E-05	0.0003	ring finger protein 167
RNF183	3.51	0.41	-1.4	3.46E-05	0.0003	ring finger protein 183
RNF185-AS1	3.67	0.43	-1.44	1.45E-05	0.0002	RNF185 antisense RNA 1
RNF219	3.2	0.25	-1.43	1.29E-05	0.0002	ring finger protein 219
RNF25	3.35	0.31	-1.44	5.00E-06	0.0002	ring finger protein 25
RNF8	3.34	0.34	-1.4	1.85E-05	0.0002	ring finger protein 8, E3 ubiquitin protein ligase
RNMTL1	3.02	0.39	-1.2	1.06E-05	0.0002	RNA methyltransferase like 1
RNU6-63P	-3.04	-1.28	0.32	3.42E-05	0.0003	RNA, U6 small nuclear 63, pseudogene
RNU6-76P	3.01	0.29	-1.3	3.66E-06	0.0002	RNA, U6 small nuclear 76, pseudogene
L-4; RNU1-2; RNU1	-3.25	-1.43	0.27	9.26E-06	0.0002	RNA, variant U1 small nuclear 18; RNA, U1 small nuclear 3; RNA, U1 small nuclear 4; RNA, U1 small nuclear 2; RNA, U1 small nuclear 1; RNA, U1 small nuclear 28, pseudogene; RNA, U1 small nuclear 27, pseudogene
L-4; RNU1-2; RNU1	-3.25	-1.43	0.27	9.26E-06	0.0002	RNA, variant U1 small nuclear 18; RNA, U1 small nuclear 3; RNA, U1 small nuclear 4; RNA, U1 small nuclear 2; RNA, U1 small nuclear 1; RNA, U1 small nuclear 28, pseudogene; RNA, U1 small nuclear 27, pseudogene
L-4; RNU1-2; RNU1	-3.36	-1.48	0.27	0.0001	0.0007	RNA, variant U1 small nuclear 18; RNA, U1 small nuclear 3; RNA, U1 small nuclear 4; RNA, U1 small nuclear 2; RNA, U1 small nuclear 1; RNA, U1 small nuclear 28, pseudogene; RNA, U1 small nuclear 27, pseudogene
L-4; RNU1-2; RNU1	-3.36	-1.48	0.27	0.0001	0.0007	RNA, variant U1 small nuclear 18; RNA, U1 small nuclear 3; RNA, U1 small nuclear 4; RNA, U1 small nuclear 2; RNA, U1 small nuclear 1; RNA, U1 small nuclear 28, pseudogene; RNA, U1 small nuclear 27, pseudogene
L-4; RNU1-2; RNU1	-3.36	-1.48	0.27	0.0001	0.0007	RNA, variant U1 small nuclear 18; RNA, U1 small nuclear 3; RNA, U1 small nuclear 4; RNA, U1 small nuclear 2; RNA, U1 small nuclear 1; RNA, U1 small nuclear 28, pseudogene; RNA, U1 small nuclear 27, pseudogene
L-4; RNU1-2; RNU1	-3.36	-1.48	0.27	0.0001	0.0007	RNA, variant U1 small nuclear 18; RNA, U1 small nuclear 3; RNA, U1 small nuclear 4; RNA, U1 small nuclear 2; RNA, U1 small nuclear 1; RNA, U1 small nuclear 28, pseudogene; RNA, U1 small nuclear 27, pseudogene
L-4; RNU1-2; RNU1	-3.36	-1.48	0.27	0.0001	0.0007	RNA, variant U1 small nuclear 18; RNA, U1 small nuclear 3; RNA, U1 small nuclear 4; RNA, U1 small nuclear 2; RNA, U1 small nuclear 1; RNA, U1 small nuclear 28, pseudogene; RNA, U1 small nuclear 27, pseudogene
NU1-2; RNU1-1; RI	-3.13	-1.28	0.36	0.0003	0.0012	RNA, variant U1 small nuclear 18; RNA, U1 small nuclear 3; RNA, U1 small nuclear 4; RNA, U1 small nuclear 2; RNA, U1 small nuclear 1; RNA, U1 small nuclear 28, pseudogene; RNA, U1 small nuclear 27, pseudogene; RNA, U1 small nuclear 13, pseudogene
NU1-2; RNU1-1; RI	-3.13	-1.28	0.36	0.0003	0.0012	RNA, variant U1 small nuclear 18; RNA, U1 small nuclear 3; RNA, U1 small nuclear 4; RNA, U1 small nuclear 2; RNA, U1 small nuclear 1; RNA, U1 small nuclear 28, pseudogene; RNA, U1 small nuclear 27, pseudogene; RNA, U1 small nuclear 13, pseudogene
RNVU1-19	-3.15	-1.36	0.29	3.87E-05	0.0003	RNA, variant U1 small nuclear 19
RNVU1-6	3.54	0.44	-1.38	9.42E-06	0.0002	RNA, variant U1 small nuclear 6
RP9	3.56	0.54	-1.29	7.33E-06	0.0002	retinitis pigmentosa 9 (autosomal dominant)
RPF2	3.55	0.39	-1.44	2.67E-06	0.0002	ribosome production factor 2 homolog
RPL13	-3.36	-1.38	0.36	1.39E-05	0.0002	ribosomal protein L13
RPL13AP3	-3.08	-1.31	0.31	1.36E-05	0.0002	ribosomal protein L13a pseudogene 3
RPL15; RPL15P3	-3.01	-1.32	0.27	1.97E-05	0.0002	ribosomal protein L15; ribosomal protein L15 pseudogene 3
PL17-C18orf32; SN	3.3	0.32	-1.4	1.55E-05	0.0002	ribosomal protein L17; RPL17-C18orf32 readthrough; small nucleolar RNA, C/D box 58B
RPL22P19	3.43	0.35	-1.43	1.32E-05	0.0002	ribosomal protein L22 pseudogene 19
RPL26L1	-3.04	-1.28	0.32	0.0002	0.0009	ribosomal protein L26-like 1
RPL29P33	3.26	0.28	-1.43	2.18E-05	0.0002	ribosomal protein L29 pseudogene 33
RPL3	-3.04	-1.28	0.32	2.57E-05	0.0003	ribosomal protein L3
RPL31	3.63	0.38	-1.48	2.67E-05	0.0003	ribosomal protein L31
RPL31P57	-3.43	-1.41	0.36	8.47E-06	0.0002	ribosomal protein L31 pseudogene 57
RPL35	-3.16	-1.31	0.35	3.20E-05	0.0003	ribosomal protein L35
RPL35A	-3.06	-1.28	0.33	5.84E-05	0.0004	ribosomal protein L35a

RPL4	3.39	0.36	-1.4	2.98E-05	0.0003	ribosomal protein L4
RPL6; RPL6P27	-3.01	-1.28	0.31	2.10E-05	0.0002	ribosomal protein L6; ribosomal protein L6 pseudogene 27
RPLP0	3.19	0.47	-1.2	1.99E-05	0.0002	ribosomal protein, large, P0
RPLP2	-3.13	-1.31	0.33	2.20E-05	0.0002	ribosomal protein, large, P2
RPS10P7	3.53	0.39	-1.43	1.46E-05	0.0002	ribosomal protein S10 pseudogene 7
RPS11	3.09	0.33	-1.3	6.55E-05	0.0004	ribosomal protein S11
RPS11P5	3.37	0.32	-1.43	8.46E-06	0.0002	ribosomal protein S11 pseudogene 5
RPS19BP1	3.26	0.31	-1.4	5.08E-06	0.0002	ribosomal protein S19 binding protein 1
RPS2; RPS2P46	3.47	0.32	-1.47	2.83E-06	0.0002	ribosomal protein S2; ribosomal protein S2 pseudogene 46
RPS25	-3.2	-1.31	0.36	0.0001	0.0006	ribosomal protein S25
RPS3A; SNORD73A	-3.11	-1.31	0.32	2.67E-05	0.0003	ribosomal protein S3A; small nucleolar RNA, C/D box 73A
RPS6KA2	-3.33	-1.41	0.32	3.26E-05	0.0003	ribosomal protein S6 kinase, 90kDa, polypeptide 2
RPS6KA5	-3.04	-1.28	0.32	8.94E-05	0.0005	ribosomal protein S6 kinase, 90kDa, polypeptide 5
RRAGA	-3.33	-1.37	0.36	5.26E-05	0.0004	Ras-related GTP binding A
RRM2	-3.05	-1.32	0.29	4.39E-06	0.0002	ribonucleotide reductase M2
RSPO3	-3.12	-1.36	0.28	6.00E-05	0.0004	R-spondin 3
RSPO4	-3.37	-1.31	0.44	2.40E-05	0.0002	R-spondin 4
RTF1	-3.16	-1.38	0.28	8.00E-06	0.0002	RTF1 homolog, Paf1/RNA polymerase II complex component
RTL1	-3.3	-1.4	0.32	2.69E-05	0.0003	retrotransposon-like 1
RTN1	-3.02	-1.28	0.31	1.83E-05	0.0002	reticulon 1
RTN4R	-3.09	-1.33	0.29	6.87E-05	0.0004	reticulon 4 receptor
RTN4RL1	3.26	0.32	-1.38	5.09E-06	0.0002	reticulon 4 receptor-like 1
RUBCN	-3.06	-1.33	0.28	2.84E-05	0.0003	RUN domain and cysteine-rich domain containing, Beclin 1-interacting protein
RUFY2	-3.22	-1.36	0.32	1.68E-05	0.0002	RUN and FYVE domain containing 2
RUNDC3B	3.31	0.33	-1.4	5.20E-06	0.0002	RUN domain containing 3B
RUNX1T1	3.51	0.41	-1.4	6.35E-06	0.0002	runt-related transcription factor 1; translocated to, 1 (cyclin D-related)
DD3; TMEM56-RW	-3.26	-1.38	0.32	5.20E-05	0.0004	RWD domain containing 3; TMEM56-RWDD3 readthrough
RXFP4	3.28	0.33	-1.38	3.58E-06	0.0002	relaxin/insulin-like family peptide receptor 4
S100A13	3.06	0.23	-1.38	3.45E-06	0.0002	S100 calcium binding protein A13
S100A5	-3.23	-1.38	0.31	8.51E-06	0.0002	S100 calcium binding protein A5
S100A6	-3	-1.33	0.25	2.19E-05	0.0002	S100 calcium binding protein A6
S100A9	3.37	0.32	-1.43	3.83E-05	0.0003	S100 calcium binding protein A9
S1PR1	-3.03	-1.32	0.28	1.62E-05	0.0002	sphingosine-1-phosphate receptor 1
SAMD1	3.52	0.43	-1.38	4.85E-06	0.0002	sterile alpha motif domain containing 1
SAMD9L	3.37	0.32	-1.43	5.90E-05	0.0004	sterile alpha motif domain containing 9-like
SAP30L	3.11	0.26	-1.38	1.22E-05	0.0002	SAP30-like
D1; MSH5; MSH5-S	-3.11	-1.38	0.26	2.67E-05	0.0003	suppressor APC domain containing 1; mutS homolog 5; MSH5-SAPCD1 readthrough (NMD candidate)
SAPCD2	-3.09	-1.28	0.35	1.65E-05	0.0002	suppressor APC domain containing 2

SAYSD1	3.3	0.32	-1.4	3.29E-06	0.0002	SAYSVFN motif domain containing 1
SBNO2	-3.02	-1.33	0.26	1.27E-05	0.0002	strawberry notch homolog 2 (Drosophila)
SCAF11	3.07	0.24	-1.38	8.25E-06	0.0002	SR-related CTD-associated factor 11
SCAPER	-3.21	-1.38	0.3	9.66E-06	0.0002	S-phase cyclin A-associated protein in the ER
NA16; MIR6516; SN	3.04	0.19	-1.41	1.18E-05	0.0002	small Cajal body-specific RNA 16; microRNA 6516; small nucleolar RNA host gene 20
SCARNA17	3.39	0.56	-1.2	3.14E-06	0.0002	small Cajal body-specific RNA 17
SCARNA5	3.32	0.26	-1.47	7.47E-06	0.0002	small Cajal body-specific RNA 5
SCG5	-3.19	-1.38	0.29	6.35E-05	0.0004	secretogranin V
SCGB2A1	-3.12	-1.36	0.28	0.0001	0.0007	secretoglobin, family 2A, member 1
SCGB3A2	-3.28	-1.43	0.28	1.34E-05	0.0002	secretoglobin, family 3A, member 2
SCN5A	-3.01	-1.31	0.28	7.59E-06	0.0002	sodium channel, voltage gated, type V alpha subunit
SCN7A	-3.22	-1.28	0.41	8.64E-06	0.0002	sodium channel, voltage gated, type VII alpha subunit
SCNM1	-3.12	-1.36	0.28	4.00E-05	0.0003	sodium channel modifier 1
SCP2D1	-3.11	-1.31	0.32	5.24E-06	0.0002	SCP2 sterol-binding domain containing 1
SDC3	-3.02	-1.28	0.31	3.34E-05	0.0003	syndecan 3
SDF2L1	-3.15	-1.37	0.28	7.40E-06	0.0002	stromal cell-derived factor 2-like 1
SDHAF2	-3.26	-1.38	0.32	5.11E-05	0.0004	succinate dehydrogenase complex assembly factor 2
SDHAP2; SDHAP1	-3.03	-1.33	0.27	6.60E-05	0.0004	succinate dehydrogenase complex subunit A, flavoprotein pseudogene 2; succinate dehydrogenase complex subunit A, flavoprotein pseudogene 1
SEC22B	-3.16	-1.38	0.28	6.09E-05	0.0004	SEC22 homolog B, vesicle trafficking protein (gene/pseudogene)
SEC61A1	3.05	0.23	-1.38	7.53E-06	0.0002	Sec61 translocon alpha 1 subunit
SELP	-3.06	-1.33	0.28	1.25E-05	0.0002	selectin P (granule membrane protein 140kDa, antigen CD62)
SEMA3C	-3.11	-1.31	0.32	3.71E-05	0.0003	sema domain, immunoglobulin domain (Ig), short basic domain, secreted, (semaphorin) 3C
SEMA5A	-3.2	-1.31	0.36	0.0001	0.0006	sema domain, seven thrombospondin repeats (type 1 and type 1-like), transmembrane domain (TM) and short cytoplasmic domain, (semaphorin) 5A
SEMA6C	-3.14	-1.37	0.28	5.50E-05	0.0004	sema domain, transmembrane domain (TM), and cytoplasmic domain, (semaphorin) 6C
SEMA6D	3.01	0.29	-1.3	1.77E-05	0.0002	sema domain, transmembrane domain (TM), and cytoplasmic domain, (semaphorin) 6D
SENP1	3.26	0.32	-1.38	6.88E-06	0.0002	SUMO1/sentrin specific peptidase 1
ENP3; SENP3-EIF4A	3.34	0.36	-1.38	1.71E-05	0.0002	SUMO1/sentrin/SMT3 specific peptidase 3; SENP3-EIF4A1 readthrough (NMD candidate)
SEPHS1	3.36	0.46	-1.29	0.0001	0.0007	selenophosphate synthetase 1
SEPN1	3.13	0.24	-1.4	5.26E-06	0.0002	selenoprotein N, 1
SEPT10	-3.16	-1.31	0.35	0.0005	0.0021	septin 10
SEPT11	-3.16	-1.38	0.28	5.64E-05	0.0004	septin 11
SEPT14	-3.12	-1.36	0.28	2.39E-05	0.0002	septin 14
SEPT3; WBP2NL	-3.23	-1.36	0.33	2.15E-05	0.0002	septin 3; WBP2 N-terminal like
SERGEF	-3.03	-1.32	0.28	1.25E-05	0.0002	secretion regulating guanine nucleotide exchange factor
SERPINA13P	-3.13	-1.28	0.36	6.94E-05	0.0004	serpin peptidase inhibitor, clade A (alpha-1 antiproteinase, antitrypsin), member 13, pseudogene
SERPINC1	-3.2	-1.31	0.36	4.46E-05	0.0003	serpin peptidase inhibitor, clade C (antithrombin), member 1
SERPINF1	3.06	0.23	-1.38	6.21E-06	0.0002	serpin peptidase inhibitor, clade F (alpha-2 antiplasmin, pigment epithelium derived factor), member 1
SERTAD2	-3.06	-1.31	0.3	2.83E-05	0.0003	SERTA domain containing 2

SERTM1	3.3	0.32	-1.4	2.61E-06	0.0002	serine-rich and transmembrane domain containing 1
SETD1B	-3	-1.28	0.31	0.0002	0.0009	SET domain containing 1B
SETD6	-3.12	-1.36	0.28	7.39E-06	0.0002	SET domain containing 6
SETDB1	-3.07	-1.34	0.28	9.85E-05	0.0005	SET domain, bifurcated 1
SF3B2	-3.23	-1.41	0.28	6.26E-06	0.0002	splicing factor 3b subunit 2
SF3B6	-3.22	-1.32	0.36	8.17E-05	0.0005	splicing factor 3b subunit 6
SFPQ	-3.37	-1.48	0.28	0.0001	0.0007	splicing factor proline/glutamine-rich
SFXN2	-3.33	-1.33	0.4	4.62E-05	0.0003	sideroflexin 2
SGIP1	-3.04	-1.38	0.22	0.0002	0.0008	SH3-domain GRB2-like (endophilin) interacting protein 1
SGK494	-3	-1.36	0.22	2.86E-05	0.0003	uncharacterized serine/threonine-protein kinase SgK494
SGSM2	3.12	0.44	-1.2	5.86E-06	0.0002	small G protein signaling modulator 2
SH2D3C	3.06	0.41	-1.2	7.53E-06	0.0002	SH2 domain containing 3C
SH2D4A	-3.01	-1.31	0.28	5.24E-06	0.0002	SH2 domain containing 4A
SH3PXD2B	-3.14	-1.37	0.28	4.31E-05	0.0003	SH3 and PX domains 2B
SH3RF1	-3.13	-1.36	0.28	2.64E-05	0.0003	SH3 domain containing ring finger 1
SHB	3.3	0.32	-1.4	4.06E-05	0.0003	Src homology 2 domain containing adaptor protein B
SHISA6	3.57	0.32	-1.51	4.63E-06	0.0002	shisa family member 6
SIAH3	-3.26	-1.35	0.36	0.0002	0.0008	siah E3 ubiquitin protein ligase family member 3
SIGLEC10	3.57	0.39	-1.44	1.11E-05	0.0002	sialic acid binding Ig-like lectin 10
SIGMAR1	-3.11	-1.32	0.32	9.87E-06	0.0002	sigma non-opioid intracellular receptor 1
SIPA1L1	-3.16	-1.47	0.19	1.08E-05	0.0002	signal-induced proliferation-associated 1 like 1
SIPA1L2	3.13	0.26	-1.38	5.22E-05	0.0004	signal-induced proliferation-associated 1 like 2
SIRT1	-3.03	-1.3	0.31	5.09E-05	0.0003	sirtuin 1
SIRT3	3.03	0.31	-1.29	7.55E-05	0.0004	sirtuin 3
SIVA1	3.02	0.39	-1.2	1.27E-05	0.0002	SIVA1, apoptosis-inducing factor
SIX6	3.42	0.39	-1.38	7.13E-05	0.0004	SIX homeobox 6
SKA3	3.06	0.32	-1.29	9.65E-06	0.0002	spindle and kinetochore associated complex subunit 3
SKIDA1	-3.24	-1.34	0.36	1.24E-05	0.0002	SKI/DACH domain containing 1
SKIV2L	3.19	0.24	-1.44	3.06E-06	0.0002	SKI2 homolog, superkiller viralicidic activity 2-like
SKIV2L	3.11	0.19	-1.44	3.97E-06	0.0002	SKI2 homolog, superkiller viralicidic activity 2-like
SKIV2L	3.11	0.19	-1.44	3.97E-06	0.0002	SKI2 homolog, superkiller viralicidic activity 2-like
SKOR2	3.51	0.43	-1.38	4.35E-06	0.0002	SKI family transcriptional corepressor 2
SLAMF7	-3.05	-1.31	0.3	1.69E-05	0.0002	SLAM family member 7
SLC12A7	3.01	0.19	-1.4	4.11E-05	0.0003	solute carrier family 12 (potassium/chloride transporter), member 7
SLC12A8	-3.19	-1.37	0.3	9.10E-06	0.0002	solute carrier family 12, member 8
SLC17A2	3.52	0.43	-1.38	6.31E-06	0.0002	solute carrier family 17, member 2
SLC18A2	-3.25	-1.37	0.33	3.54E-05	0.0003	solute carrier family 18 (vesicular monoamine transporter), member 2
SLC1A7	-3.19	-1.33	0.34	8.91E-06	0.0002	solute carrier family 1 (glutamate transporter), member 7

SLC20A1	-3.01	-1.36	0.23	6.60E-06	0.0002	solute carrier family 20 (phosphate transporter), member 1
SLC25A29	3.01	0.14	-1.44	2.01E-05	0.0002	solute carrier family 25 (mitochondrial carnitine/acylcarnitine carrier), member 29
SLC25A30	3.3	0.32	-1.4	1.49E-05	0.0002	solute carrier family 25, member 30
SLC25A6	3.02	0.39	-1.2	1.11E-05	0.0002	solute carrier family 25 (mitochondrial carrier; adenine nucleotide translocator), member 6
SLC25A6	3.02	0.39	-1.2	1.11E-05	0.0002	solute carrier family 25 (mitochondrial carrier; adenine nucleotide translocator), member 6
SLC26A3	-3.44	-1.46	0.32	1.84E-05	0.0002	solute carrier family 26 (anion exchanger), member 3
SLC29A4	3.37	0.34	-1.41	0.0002	0.0009	solute carrier family 29 (equilibrative nucleoside transporter), member 4
SLC2A11	3.06	0.32	-1.29	2.69E-05	0.0003	solute carrier family 2 (facilitated glucose transporter), member 11
SLC2A13	3.42	0.39	-1.38	9.75E-06	0.0002	solute carrier family 2 (facilitated glucose transporter), member 13
SLC34A3	-3.06	-1.33	0.28	0.0002	0.0009	solute carrier family 34 (type II sodium/phosphate cotransporter), member 3
SLC35D3	-3.12	-1.36	0.28	3.66E-05	0.0003	solute carrier family 35, member D3
SLC37A4	3.39	0.32	-1.44	5.09E-06	0.0002	solute carrier family 37 (glucose-6-phosphate transporter), member 4
SLC38A2	-3.15	-1.38	0.27	8.13E-06	0.0002	solute carrier family 38, member 2
SLC38A5	-3.14	-1.37	0.28	2.22E-05	0.0002	solute carrier family 38, member 5
SLC39A13	-3.06	-1.4	0.22	9.37E-06	0.0002	solute carrier family 39 (zinc transporter), member 13
SLC44A1	3.4	0.35	-1.41	8.58E-06	0.0002	solute carrier family 44 (choline transporter), member 1
SLC5A6	-3.1	-1.28	0.35	4.80E-06	0.0002	solute carrier family 5 (sodium/multivitamin and iodide cotransporter), member 6
SLC7A1	3.06	0.41	-1.2	1.02E-05	0.0002	solute carrier family 7 (cationic amino acid transporter, y+ system), member 1
SLC7A14	3.59	0.37	-1.47	1.15E-05	0.0002	solute carrier family 7, member 14
SLC9A1	-3.08	-1.36	0.26	9.48E-06	0.0002	solute carrier family 9, subfamily A (NHE1, cation proton antiporter 1), member 1
SLCO2A1	-3.12	-1.36	0.28	7.02E-06	0.0002	solute carrier organic anion transporter family, member 2A1
SLCO3A1	3.26	0.32	-1.38	1.73E-05	0.0002	solute carrier organic anion transporter family, member 3A1
SLFN13	-3.13	-1.36	0.28	2.61E-05	0.0003	schlafen family member 13
SLX1A-SULT1A3; S	3.54	0.4	-1.43	4.28E-06	0.0002	SLX1 homolog A, structure-specific endonuclease subunit; sulfotransferase family 1A member 4; SLX1 homolog B, structure-specific endonuclease subunit; SLX1A-SULT1A3 readthrough (NMD candidate); SLX1B-SULT1A4 readthrough (NMD candidate); sulfotransferase family 1A member 3
SLX1B-SULT1A4; S	3.54	0.4	-1.43	1.45E-05	0.0002	SLX1 homolog A, structure-specific endonuclease subunit; sulfotransferase family 1A member 4; SLX1 homolog B, structure-specific endonuclease subunit; SLX1B-SULT1A4 readthrough (NMD candidate); SLX1A-SULT1A3 readthrough (NMD candidate); sulfotransferase family 1A member 3
SMARCC2	-3.07	-1.36	0.25	6.06E-06	0.0002	SWI/SNF related, matrix associated, actin dependent regulator of chromatin, subfamily c, member 2
SMARCD1	-3.01	-1.42	0.17	7.78E-05	0.0005	SWI/SNF related, matrix associated, actin dependent regulator of chromatin, subfamily d, member 1
SMCR8	-3.12	-1.36	0.28	4.67E-06	0.0002	Smith-Magenis syndrome chromosome region, candidate 8
SMG9	3.01	0.3	-1.29	2.28E-05	0.0002	SMG9 nonsense mediated mRNA decay factor
SMIM10L2A	3.3	0.32	-1.4	8.00E-05	0.0005	small integral membrane protein 10 like 2A
SMIM10L2B	-3.16	-1.31	0.35	9.15E-06	0.0002	small integral membrane protein 10 like 2B
SMIM11P1	-3.12	-1.36	0.28	9.52E-06	0.0002	small integral membrane protein 11 pseudogene 1
SMIM20	-3.37	-1.38	0.37	0.0001	0.0007	small integral membrane protein 20
SMPD1	3.34	0.34	-1.4	3.19E-06	0.0002	sphingomyelin phosphodiesterase 1, acid lysosomal
SMR3A; SMR3B	-3.29	-1.4	0.32	1.37E-05	0.0002	submaxillary gland androgen regulated protein 3A; submaxillary gland androgen regulated protein 3B
SMU1	-3.09	-1.41	0.22	1.09E-05	0.0002	smu-1 suppressor of mec-8 and unc-52 homolog (C. elegans)
SNAP25	-3.08	-1.38	0.24	9.13E-05	0.0005	synaptosome associated protein 25kDa
SNAPIN	-3.14	-1.3	0.36	1.91E-05	0.0002	SNAP-associated protein

SNORA22	-3.21	-1.34	0.35	1.24E-05	0.0002	small nucleolar RNA, H/ACA box 22
SNORA24; SNHG8	-3.16	-1.38	0.28	1.09E-05	0.0002	small nucleolar RNA, H/ACA box 24; small nucleolar RNA host gene 8
SNORA28	3.26	0.32	-1.38	3.43E-06	0.0002	small nucleolar RNA, H/ACA box 28
SNORA52; RPLP2	-3	-1.33	0.25	1.08E-05	0.0002	small nucleolar RNA, H/ACA box 52; ribosomal protein, large, P2
SNORA64; RPS2	-3.06	-1.33	0.28	3.92E-05	0.0003	small nucleolar RNA, H/ACA box 64; ribosomal protein S2
SNORA71A	3.02	0.39	-1.2	7.18E-05	0.0004	small nucleolar RNA, H/ACA box 71A
SNORA71C	-3.16	-1.38	0.28	0.0001	0.0007	small nucleolar RNA, H/ACA box 71C
SNORA71D	-3.22	-1.36	0.32	0.0001	0.0007	small nucleolar RNA, H/ACA box 71D
SNORD104	-3.04	-1.28	0.32	0.0002	0.0008	small nucleolar RNA, C/D box 104
SNORD115-25	3.3	0.32	-1.4	8.83E-06	0.0002	small nucleolar RNA, C/D box 115-25
SNORD115-3	3.78	0.44	-1.48	5.46E-06	0.0002	small nucleolar RNA, C/D box 115-3
SNORD116-18	3.05	0.19	-1.41	0.0002	0.0010	small nucleolar RNA, C/D box 116-18
D116-19; SNORD1	-3.12	-1.36	0.28	2.65E-05	0.0003	small nucleolar RNA, C/D box 116-19; small nucleolar RNA, C/D box 116-17
D116-19; SNORD1	-3.12	-1.36	0.28	2.65E-05	0.0003	small nucleolar RNA, C/D box 116-19; small nucleolar RNA, C/D box 116-17
SNORD116-24	-3.01	-1.31	0.28	4.82E-05	0.0003	small nucleolar RNA, C/D box 116-24
IORD13; SNORD13	-3.29	-1.31	0.4	3.10E-05	0.0003	small nucleolar RNA, C/D box 13; small nucleolar RNA, C/D box 13 pseudogene 3
SNORD43; RPL3	-3.01	-1.31	0.28	5.69E-06	0.0002	small nucleolar RNA, C/D box 43; ribosomal protein L3
SNORD95; GNB2L1	-3.13	-1.28	0.36	3.88E-05	0.0003	small nucleolar RNA, C/D box 95; guanine nucleotide binding protein (G protein), beta polypeptide 2-like 1
SNRPA1	3.42	0.39	-1.38	5.60E-05	0.0004	small nuclear ribonucleoprotein polypeptide A
SNRPF	3.06	0.21	-1.4	1.52E-05	0.0002	small nuclear ribonucleoprotein polypeptide F
SNX12	-3.27	-1.28	0.43	2.94E-05	0.0003	sorting nexin 12
SNX18	-3.07	-1.31	0.31	8.68E-06	0.0002	sorting nexin 18
SNX31	3.04	0.22	-1.38	0.0004	0.0016	sorting nexin 31
SNX9	-3.25	-1.38	0.32	0.0002	0.0009	sorting nexin 9
SOHLH1	-3.14	-1.37	0.28	2.94E-05	0.0003	spermatogenesis and oogenesis specific basic helix-loop-helix 1
SORBS1	-3.09	-1.36	0.26	1.58E-05	0.0002	sorbin and SH3 domain containing 1
SOS2	-3.02	-1.41	0.18	9.04E-05	0.0005	SOS Ras/Rho guanine nucleotide exchange factor 2
SOST	3.26	0.32	-1.38	0.0002	0.0010	sclerostin
SOX18	-3.15	-1.32	0.33	2.92E-05	0.0003	SRY box 18
SOX4	3.12	0.26	-1.38	5.84E-06	0.0002	SRY box 4
SOX6	-3.16	-1.38	0.28	3.05E-05	0.0003	SRY box 6
SP8	-3.17	-1.38	0.28	1.49E-05	0.0002	Sp8 transcription factor
SPAG6	-3.09	-1.28	0.35	1.43E-05	0.0002	sperm associated antigen 6
SPAG7	-3.03	-1.31	0.29	6.29E-05	0.0004	sperm associated antigen 7
SPAG8	3.15	0.45	-1.2	1.53E-05	0.0002	sperm associated antigen 8
SPANXB1	3.09	0.34	-1.29	1.01E-05	0.0002	SPANX family, member B1
SPANXB1	3.09	0.34	-1.29	1.01E-05	0.0002	SPANX family, member B1
SPATA16	-3.1	-1.39	0.24	4.56E-05	0.0003	spermatogenesis associated 16

C1; SPATA31C2; SP	3.11	0.35	-1.29	8.94E-06	0.0002	SPATA31 subfamily C, member 1; SPATA31 subfamily C, member 2; SPATA31 subfamily A, member 1
SPATA31D5P	-3.2	-1.33	0.35	4.91E-06	0.0002	SPATA31 subfamily D, member 5, pseudogene
SPATA41	-3.07	-1.3	0.32	2.06E-05	0.0002	spermatogenesis associated 41 (non-protein coding)
SPCS1	3.12	0.21	-1.43	3.41E-05	0.0003	signal peptidase complex subunit 1
SPCS3	-3.03	-1.35	0.25	8.38E-06	0.0002	signal peptidase complex subunit 3
SPDEF	3.31	0.32	-1.4	6.53E-05	0.0004	SAM pointed domain containing ETS transcription factor
SPDYE3	3.37	0.32	-1.43	2.68E-05	0.0003	speedy/RINGO cell cycle regulator family member E3
SPECC1	-3.01	-1.28	0.31	3.47E-06	0.0002	sperm antigen with calponin homology and coiled-coil domains 1
SPEM1	3.18	0.28	-1.39	7.66E-06	0.0002	spermatid maturation 1
SPEN	3.19	0.47	-1.2	4.02E-06	0.0002	spen family transcriptional repressor
SPHK1	-3.06	-1.33	0.28	1.80E-05	0.0002	sphingosine kinase 1
SPIB	3.35	0.36	-1.38	2.83E-05	0.0003	Spi-B transcription factor (Spi-1/PU.1 related)
SPOCD1	3.26	0.32	-1.38	2.18E-05	0.0002	SPOC domain containing 1
SPRR2B	-3.2	-1.36	0.32	1.73E-05	0.0002	small proline-rich protein 2B
SPRR2D	-3.16	-1.38	0.28	5.74E-05	0.0004	small proline-rich protein 2D
SPTB	3.37	0.32	-1.43	9.00E-06	0.0002	spectrin, beta, erythrocytic
SPTBN4	3.3	0.29	-1.44	3.90E-06	0.0002	spectrin, beta, non-erythrocytic 4
SRBD1	-3.11	-1.31	0.32	8.85E-05	0.0005	S1 RNA binding domain 1
SREBF1	-3.01	-1.31	0.28	1.19E-05	0.0002	sterol regulatory element binding transcription factor 1
SRGAP3	3.26	0.41	-1.29	3.59E-06	0.0002	SLIT-ROBO Rho GTPase activating protein 3
SRP54	-3.18	-1.43	0.24	6.77E-05	0.0004	signal recognition particle 54kDa
SRSF4	3.61	0.45	-1.4	3.14E-06	0.0002	serine/arginine-rich splicing factor 4
SSB	-3.03	-1.31	0.29	0.0009	0.0035	Sjogren syndrome antigen B (autoantigen La)
SSH3	-3.19	-1.37	0.3	1.04E-05	0.0002	slingshot protein phosphatase 3
SSR4	-3.15	-1.28	0.38	1.00E-05	0.0002	signal sequence receptor, delta
SSTR1	3.1	0.43	-1.2	0.0002	0.0009	somatostatin receptor 1
X4B; SSX4; SSX2B; S	3.36	0.35	-1.4	3.70E-06	0.0002	synovial sarcoma, X breakpoint 4B; synovial sarcoma, X breakpoint 4; synovial sarcoma, X breakpoint 2B; synovial sarcoma, X breakpoint 2
X4B; SSX4; SSX2B; S	3.46	0.35	-1.44	8.26E-06	0.0002	synovial sarcoma, X breakpoint 4B; synovial sarcoma, X breakpoint 4; synovial sarcoma, X breakpoint 2B; synovial sarcoma, X breakpoint 2
ST5	3.42	0.39	-1.38	7.28E-06	0.0002	suppression of tumorigenicity 5
ST6GAL1	-3.01	-1.31	0.28	0.0001	0.0006	ST6 beta-galactosamide alpha-2,6-sialyltranferase 1
ST6GAL2	3.28	0.33	-1.38	8.31E-06	0.0002	ST6 beta-galactosamide alpha-2,6-sialyltranferase 2
STAC3; R3HDM2	-3.19	-1.38	0.29	1.73E-05	0.0002	SH3 and cysteine rich domain 3; R3H domain containing 2
STAR	-3	-1.32	0.26	5.00E-06	0.0002	steroidogenic acute regulatory protein
STAT3	3.36	0.34	-1.4	1.80E-05	0.0002	signal transducer and activator of transcription 3 (acute-phase response factor)
STAU1	-3.2	-1.31	0.36	3.70E-05	0.0003	staufen double-stranded RNA binding protein 1
STEAP1B	3.52	0.43	-1.38	5.12E-06	0.0002	STEAP family member 1B
STGC3	3.37	0.32	-1.43	9.00E-06	0.0002	uncharacterized STGC3
STIM1	3.21	0.39	-1.29	0.0002	0.0010	stromal interaction molecule 1

STK11IP	3.16	0.36	-1.3	3.41E-05	0.0003	serine/threonine kinase 11 interacting protein
STK31	-3.03	-1.32	0.28	1.51E-05	0.0002	serine/threonine kinase 31
STK32A	3.28	0.31	-1.4	1.82E-05	0.0002	serine/threonine kinase 32A
STK32B	3.18	0.38	-1.29	6.97E-06	0.0002	serine/threonine kinase 32B
STK33	-3.18	-1.31	0.36	3.91E-06	0.0002	serine/threonine kinase 33
STK39	-3.21	-1.34	0.34	0.0001	0.0007	serine threonine kinase 39
STMN1	-3.07	-1.3	0.32	6.96E-06	0.0002	stathmin 1
STRN	-3.01	-1.31	0.28	4.80E-05	0.0003	striatin, calmodulin binding protein
SUCLG1	3.03	0.31	-1.29	1.75E-05	0.0002	succinate-CoA ligase, alpha subunit
SUFU	3.23	0.39	-1.3	5.97E-06	0.0002	suppressor of fused homolog (Drosophila)
SULT1B1	3.06	0.23	-1.38	5.37E-06	0.0002	sulfotransferase family 1B member 1
SULT2A1	3.1	0.34	-1.29	6.77E-06	0.0002	sulfotransferase family 2A member 1
SUZ12	3.51	0.41	-1.4	5.44E-06	0.0002	SUZ12 polycomb repressive complex 2 subunit
SVIL	3.15	0.28	-1.38	6.23E-06	0.0002	supervillin
SYCE1; SPRNP1	-3.01	-1.31	0.28	7.90E-06	0.0002	synaptonemal complex central element protein 1; shadow of prion protein homolog (zebrafish) pseudogene 1
SYCE2	3.25	0.26	-1.44	2.20E-05	0.0002	synaptonemal complex central element protein 2
SYN3	3.54	0.44	-1.38	3.95E-06	0.0002	synapsin III
SYT12	-3.05	-1.31	0.29	9.47E-06	0.0002	synaptotagmin XII
SYT2	-3.16	-1.38	0.28	9.36E-06	0.0002	synaptotagmin II
SYT5	3.39	0.32	-1.44	4.46E-05	0.0003	synaptotagmin V
SZRD1	-3.22	-1.33	0.36	1.24E-05	0.0002	SUZ RNA binding domain containing 1
TAAR2	-3.2	-1.31	0.37	4.67E-06	0.0002	trace amine associated receptor 2
TAC3	-3.02	-1.33	0.26	2.95E-05	0.0003	tachykinin 3
TACO1	-3.18	-1.35	0.32	2.37E-05	0.0002	translational activator of mitochondrially encoded cytochrome c oxidase I
TAF5L	3.26	0.32	-1.38	3.46E-06	0.0002	TAF5-like RNA polymerase II, p300/CBP-associated factor (PCAF)-associated factor, 65kDa
TAGLN	3.56	0.45	-1.38	2.31E-06	0.0002	transgelin
TALDO1	-3.04	-1.28	0.32	0.0001	0.0006	transaldolase 1
TANGO6	3.29	0.4	-1.31	1.29E-05	0.0002	transport and golgi organization 6 homolog
TARDBP	-3.1	-1.36	0.27	1.78E-05	0.0002	TAR DNA binding protein
TAS2R1	3.53	0.39	-1.43	8.76E-06	0.0002	taste receptor, type 2, member 1
TAS2R46	-3.24	-1.37	0.32	0.0002	0.0008	taste receptor, type 2, member 46
TAS2R60	-3.16	-1.38	0.28	3.36E-05	0.0003	taste receptor, type 2, member 60
TBC1D22A-AS1	-3.36	-1.38	0.36	4.77E-05	0.0003	TBC1D22A antisense RNA 1
TBC1D25	3.37	0.32	-1.43	3.11E-06	0.0002	TBC1 domain family, member 25
TBC1D3I; TBC1D3I	-3.04	-1.28	0.32	1.00E-05	0.0002	TBC1 domain family, member 3C; TBC1 domain family, member 3; TBC1 domain family, member 3H; TBC1 domain family, member 3L; TBC1 domain family, member 3I; TBC1 domain family, member 3E; TBC1 domain family, member 3J; TBC1 domain family, member 3B; TBC1 domain family, member 3K; chemokine (C-C motif) ligand 3 pseudogene 1
TBC1D3I; TBC1D3I	-3.09	-1.28	0.35	1.07E-05	0.0002	TBC1 domain family, member 3C; TBC1 domain family, member 3; TBC1 domain family, member 3H; TBC1 domain family, member 3L; TBC1 domain family, member 3I; TBC1 domain family, member 3E; TBC1 domain family, member 3J; TBC1 domain family, member 3B; TBC1 domain family, member 3K; chemokine (C-C motif) ligand 3 pseudogene 1
TBCC	-3.11	-1.41	0.23	5.75E-06	0.0002	tubulin folding cofactor C
TBKBP1	3.31	0.32	-1.4	3.85E-06	0.0002	TBK1 binding protein 1

TBX2	3.34	0.36	-1.38	1.48E-05	0.0002	T-box 2
TBX4	-3.06	-1.33	0.28	9.17E-06	0.0002	T-box 4
TCEAL8	-3.09	-1.28	0.35	9.84E-06	0.0002	transcription elongation factor A (SII)-like 8
TCEB1	-3.06	-1.33	0.28	4.58E-05	0.0003	transcription elongation factor B (SIII), polypeptide 1 (15kDa, elongin C)
TCF20	-3.15	-1.32	0.33	2.80E-05	0.0003	transcription factor 20 (AR1)
TCF7	3.13	0.36	-1.29	4.50E-05	0.0003	transcription factor 7 (T-cell specific, HMG-box)
TCHH	3.23	0.3	-1.39	4.17E-06	0.0002	trichohyalin
TCTEX1D4	3.26	0.32	-1.38	2.18E-05	0.0002	Tctex1 domain containing 4
TDG	-3.28	-1.28	0.44	0.0002	0.0009	thymine DNA glycosylase
TEKT4	3.06	0.41	-1.2	2.29E-05	0.0002	tektin 4
TEKT4P2	-3.38	-1.36	0.39	0.0001	0.0006	tektin 4 pseudogene 2
TEKT4P2; MAFIP	-3.32	-1.41	0.32	0.0002	0.0007	tektin 4 pseudogene 2; MAFF interacting protein (pseudogene)
TENM2	3.29	0.32	-1.4	1.27E-05	0.0002	teneurin transmembrane protein 2
TESC	3.18	0.38	-1.29	7.61E-06	0.0002	tescalcin
TET1	3.18	0.38	-1.29	3.69E-05	0.0003	tet methylcytosine dioxygenase 1
TET2	-3.16	-1.38	0.28	3.42E-06	0.0002	tet methylcytosine dioxygenase 2
TEX19	3.26	0.32	-1.38	3.43E-06	0.0002	testis expressed 19
TEX26	-3.07	-1.34	0.28	1.68E-05	0.0002	testis expressed 26
TEX261	-3.08	-1.31	0.31	4.12E-06	0.0002	testis expressed 261
28; TEX28P2; TEX2	3.12	0.35	-1.29	4.67E-05	0.0003	testis expressed 28; testis expressed 28 pseudogene 2; testis expressed 28 pseudogene 1
28; TEX28P2; TEX2	3.12	0.35	-1.29	4.67E-05	0.0003	testis expressed 28; testis expressed 28 pseudogene 2; testis expressed 28 pseudogene 1
TFAM	3.26	0.32	-1.38	0.0001	0.0005	transcription factor A, mitochondrial
TFAP4	3.43	0.36	-1.42	5.19E-05	0.0004	transcription factor AP-4 (activating enhancer binding protein 4)
TFDP1	-3.13	-1.33	0.32	2.67E-05	0.0003	transcription factor Dp-1
TFDP2	3.22	0.26	-1.43	2.85E-05	0.0003	transcription factor Dp-2 (E2F dimerization partner 2)
TFR2	3.14	0.21	-1.44	2.83E-05	0.0003	transferrin receptor 2
TGFB3	3.08	0.23	-1.39	3.50E-05	0.0003	transforming growth factor beta 3
TGM5	3.29	0.3	-1.41	3.53E-05	0.0003	transglutaminase 5
THAP2	3.53	0.38	-1.44	1.26E-05	0.0002	THAP domain containing, apoptosis associated protein 2
THOC3	-3.14	-1.3	0.36	8.23E-06	0.0002	THO complex 3
THOC3	-3.09	-1.28	0.35	1.00E-05	0.0002	THO complex 3
THOC6	-3.06	-1.33	0.28	4.23E-06	0.0002	THO complex 6
THRA; NR1D1	-3	-1.31	0.27	4.79E-06	0.0002	thyroid hormone receptor, alpha; nuclear receptor subfamily 1, group D, member 1
THTPA	-3.46	-1.31	0.48	3.70E-05	0.0003	thiamine triphosphatase
TIGD5	-3.19	-1.3	0.38	1.63E-05	0.0002	tigger transposable element derived 5
TIMM10B	-3.12	-1.36	0.28	3.66E-05	0.0003	translocase of inner mitochondrial membrane 10 homolog B (yeast)
TIMP2; CEP295NL	3.37	0.31	-1.44	1.31E-05	0.0002	TIMP metallopeptidase inhibitor 2; CEP295 N-terminal like
TIRAP	-3.38	-1.36	0.4	2.60E-05	0.0003	toll-interleukin 1 receptor (TIR) domain containing adaptor protein

TISP43; LOC646743	-3.5	-1.38	0.43	0.0002	0.0009	uncharacterized LOC150527; uncharacterized LOC646743
TISP43; LOC646743	-3.5	-1.38	0.43	0.0002	0.0009	uncharacterized LOC150527; uncharacterized LOC646743
TJP2	3.25	0.32	-1.38	9.28E-06	0.0002	tight junction protein 2
TLDC2	-3.52	-1.37	0.44	4.39E-05	0.0003	TBC/LysM-associated domain containing 2
TLR8	-3.11	-1.38	0.25	3.70E-05	0.0003	toll-like receptor 8
TM7SF2	-3.12	-1.36	0.28	5.86E-06	0.0002	transmembrane 7 superfamily member 2
TM9SF3	-3.01	-1.31	0.28	4.50E-05	0.0003	transmembrane 9 superfamily member 3
TMED1	3.19	0.47	-1.2	1.65E-05	0.0002	transmembrane p24 trafficking protein 1
TMEM104	3.22	0.26	-1.43	8.24E-06	0.0002	transmembrane protein 104
TMEM109	-3.01	-1.31	0.28	1.28E-05	0.0002	transmembrane protein 109
TMEM11	3.48	0.4	-1.4	3.34E-05	0.0003	transmembrane protein 11
110-MUSTN1; TME	-3.03	-1.28	0.32	3.56E-06	0.0002	TMEM110-MUSTN1 readthrough; transmembrane protein 110
TMEM120A	-3.05	-1.35	0.26	2.63E-05	0.0003	transmembrane protein 120A
TMEM134	-3.04	-1.28	0.32	1.11E-05	0.0002	transmembrane protein 134
TMEM136	-3.17	-1.36	0.3	5.72E-05	0.0004	transmembrane protein 136
TMEM14EP	-3.07	-1.38	0.24	7.28E-05	0.0004	transmembrane protein 14E, pseudogene
TMEM151A	-3.14	-1.28	0.37	4.06E-05	0.0003	transmembrane protein 151A
TMEM17	-3.03	-1.32	0.28	3.61E-06	0.0002	transmembrane protein 17
TMEM174	-3.01	-1.3	0.29	4.16E-06	0.0002	transmembrane protein 174
TMEM175	-3.09	-1.36	0.26	1.82E-05	0.0002	transmembrane protein 175
TMEM18	3.02	0.39	-1.2	6.10E-05	0.0004	transmembrane protein 18
TMEM199	3.47	0.39	-1.4	6.94E-06	0.0002	transmembrane protein 199
TMEM200B	3.1	0.43	-1.2	1.41E-05	0.0002	transmembrane protein 200B
TMEM217	-3.02	-1.36	0.23	3.09E-05	0.0003	transmembrane protein 217
TMEM221	-3.03	-1.28	0.32	1.07E-05	0.0002	transmembrane protein 221
TMEM266	-3.09	-1.28	0.35	5.30E-05	0.0004	transmembrane protein 266
TMEM41A	-3.14	-1.35	0.3	1.34E-05	0.0002	transmembrane protein 41A
TMEM55B	-3.07	-1.34	0.28	3.65E-05	0.0003	transmembrane protein 55B
TMEM61	3.02	0.39	-1.2	7.80E-05	0.0005	transmembrane protein 61
TMEM62	-3.03	-1.31	0.29	4.66E-06	0.0002	transmembrane protein 62
TMEM88	3.34	0.44	-1.3	3.63E-06	0.0002	transmembrane protein 88
TMEM97	3.09	0.23	-1.4	9.80E-06	0.0002	transmembrane protein 97
TMOD4; VPS72	-3.07	-1.28	0.34	2.81E-05	0.0003	tropomodulin 4 (muscle); vacuolar protein sorting 72 homolog (S. cerevisiae)
TMPRSS13	-3.09	-1.36	0.26	1.18E-05	0.0002	transmembrane protease, serine 13
TMPRSS5	-3.06	-1.33	0.28	5.43E-06	0.0002	transmembrane protease, serine 5
TMPRSS7	-3.04	-1.38	0.22	1.10E-05	0.0002	transmembrane protease, serine 7
4XP6; TMSB4X; TMS	3.3	0.32	-1.4	3.06E-06	0.0002	thymosin beta 4, X-linked pseudogene 6; thymosin beta 4, X-linked; thymosin beta 4, X-linked pseudogene 4
TMUB1	3.26	0.32	-1.38	1.55E-05	0.0002	transmembrane and ubiquitin-like domain containing 1

TNFAIP3	-3.1	-1.38	0.25	9.99E-06	0.0002	tumor necrosis factor, alpha-induced protein 3
TNFRSF10C	-3.03	-1.28	0.32	1.34E-05	0.0002	tumor necrosis factor receptor superfamily, member 10c, decoy without an intracellular domain
TNFRSF21	3.19	0.27	-1.4	6.62E-06	0.0002	tumor necrosis factor receptor superfamily, member 21
TNFSF4	-3.07	-1.31	0.31	3.66E-06	0.0002	tumor necrosis factor (ligand) superfamily, member 4
TNIP1	3.44	0.38	-1.4	4.19E-05	0.0003	TNFAIP3 interacting protein 1
TNK2-AS1	3.38	0.44	-1.31	3.40E-05	0.0003	TNK2 antisense RNA 1
TNNC2	3.11	0.35	-1.29	1.29E-05	0.0002	troponin C type 2 (fast)
TNNI2	3.05	0.41	-1.2	1.88E-05	0.0002	troponin I type 2 (skeletal, fast)
TNPO1	3.66	0.48	-1.39	3.69E-06	0.0002	transportin 1
TOB1	3.49	0.41	-1.39	7.46E-06	0.0002	transducer of ERBB2, 1
TOM1L1	-3.08	-1.32	0.3	9.99E-06	0.0002	target of myb1 like 1 membrane trafficking protein
TOMM5; FBXO10	3.26	0.32	-1.38	2.68E-06	0.0002	translocase of outer mitochondrial membrane 5 homolog (yeast); F-box protein 10
TOMM6; PRICKLE4	-3.24	-1.28	0.42	5.10E-05	0.0003	translocase of outer mitochondrial membrane 6 homolog (yeast); prickle homolog 4
TOMM70A	-3.16	-1.38	0.28	3.36E-05	0.0003	translocase of outer mitochondrial membrane 70 homolog A (S. cerevisiae)
TONSL	-3.23	-1.41	0.28	4.12E-05	0.0003	tonsoku-like, DNA repair protein
TOPAZ1	-3.2	-1.31	0.36	7.59E-06	0.0002	testis and ovary specific PAZ domain containing 1
TOX3	-3.1	-1.32	0.31	2.48E-05	0.0002	TOX high mobility group box family member 3
TP53TG1	3.4	0.32	-1.44	1.63E-05	0.0002	TP53 target 1 (non-protein coding)
TP73-AS1	-3.01	-1.31	0.28	7.90E-06	0.0002	TP73 antisense RNA 1
TPGS2	3.29	0.43	-1.29	1.62E-05	0.0002	tubulin polyglutamylase complex subunit 2
TPH2	-3.05	-1.28	0.33	6.93E-06	0.0002	tryptophan hydroxylase 2
TPM4	-3.17	-1.28	0.39	0.0005	0.0019	tropomyosin 4
TPRG1L	3.48	0.36	-1.44	3.64E-05	0.0003	tumor protein p63 regulated 1-like
TPTE	-3.12	-1.36	0.28	1.21E-05	0.0002	transmembrane phosphatase with tensin homology
TRABD2B	3.26	0.32	-1.38	3.18E-05	0.0003	TraB domain containing 2B
TRAK1	3.16	0.23	-1.43	2.56E-05	0.0003	trafficking protein, kinesin binding 1
TRANK1	-3.02	-1.33	0.26	2.44E-05	0.0002	tetratricopeptide repeat and ankyrin repeat containing 1
TRIB3	-3.1	-1.3	0.34	8.83E-05	0.0005	tribbles pseudokinase 3
TRIM24	3.3	0.32	-1.4	3.06E-05	0.0003	tripartite motif containing 24
TRIM29	-3.03	-1.3	0.3	3.88E-05	0.0003	tripartite motif containing 29
TRIM31	3.37	0.32	-1.43	7.19E-06	0.0002	tripartite motif containing 31
TRIM40	-3.04	-1.28	0.32	7.67E-06	0.0002	tripartite motif containing 40
TRIM42	3.1	0.43	-1.2	9.30E-06	0.0002	tripartite motif containing 42
TRIM52	-3.18	-1.32	0.35	3.13E-05	0.0003	tripartite motif containing 52
[illegible]M6-TRIM34; TRIM3	-3.01	-1.31	0.28	2.69E-05	0.0003	tripartite motif containing 6; TRIM6-TRIM34 readthrough; tripartite motif containing 34; tripartite motif containing 78, pseudogene
TRIM66	3.31	0.34	-1.39	3.07E-06	0.0002	tripartite motif containing 66
TRIM9	3.04	0.4	-1.2	8.30E-06	0.0002	tripartite motif containing 9
TRIML2	-3.02	-1.38	0.21	4.09E-05	0.0003	tripartite motif family like 2

TRPV2	-3.08	-1.38	0.24	4.92E-05	0.0003	transient receptor potential cation channel, subfamily V, member 2
TSC1	-3.03	-1.32	0.28	4.39E-06	0.0002	tuberous sclerosis 1
TSC22D2	3.09	0.21	-1.42	2.64E-05	0.0003	TSC22 domain family, member 2
TSEN15	-3.05	-1.37	0.24	5.03E-05	0.0003	TSEN15 tRNA splicing endonuclease subunit
TSPAN1	-3.1	-1.38	0.25	1.41E-05	0.0002	tetraspanin 1
TSPAN2	-3.12	-1.36	0.28	0.0001	0.0007	tetraspanin 2
TSPAN33	-3.01	-1.31	0.28	3.24E-05	0.0003	tetraspanin 33
TSPAN4	-3.16	-1.34	0.32	0.0001	0.0006	tetraspanin 4
TSPAN5	-3.11	-1.36	0.27	5.58E-05	0.0004	tetraspanin 5
TSSK6	-3.03	-1.31	0.29	1.19E-05	0.0002	testis-specific serine kinase 6
TST	-3.36	-1.35	0.4	0.0004	0.0016	thiosulfate sulfurtransferase (rhodanese)
TTC7A	3.3	0.31	-1.41	4.89E-05	0.0003	tetratricopeptide repeat domain 7A
TTC9	3.04	0.4	-1.2	0.0002	0.0007	tetratricopeptide repeat domain 9
TUBA1C	-3.09	-1.28	0.35	0.0001	0.0006	tubulin, alpha 1c
TUBA8; PEX26	-3.14	-1.31	0.34	8.11E-06	0.0002	tubulin, alpha 8; peroxisomal biogenesis factor 26
TUBB2A	3.41	0.32	-1.44	3.53E-06	0.0002	tubulin, beta 2A class IIa
TUBGCP2	-3.21	-1.41	0.27	1.60E-05	0.0002	tubulin, gamma complex associated protein 2
TULP1	3.36	0.36	-1.39	4.09E-06	0.0002	tubby like protein 1
TVP23A	-3.06	-1.33	0.28	7.53E-06	0.0002	trans-golgi network vesicle protein 23 homolog A (S. cerevisiae)
TWIST1	3.32	0.43	-1.3	9.05E-05	0.0005	twist family bHLH transcription factor 1
TYW1B	3.26	0.32	-1.38	9.04E-05	0.0005	tRNA-yW synthesizing protein 1 homolog B (S. cerevisiae)
U2AF2	-3.07	-1.28	0.34	1.48E-05	0.0002	U2 small nuclear RNA auxiliary factor 2
UAP1	3.53	0.39	-1.43	1.05E-05	0.0002	UDP-N-acetylglucosamine pyrophosphorylase 1
UBA5	-3.13	-1.28	0.36	1.07E-05	0.0002	ubiquitin-like modifier activating enzyme 5
UBBP4	-3.37	-1.43	0.32	4.46E-05	0.0003	ubiquitin B pseudogene 4
UBE2D4	-3.11	-1.31	0.32	2.32E-05	0.0002	ubiquitin-conjugating enzyme E2D 4 (putative)
UBE2DNL	-3.21	-1.28	0.4	6.59E-05	0.0004	ubiquitin conjugating enzyme E2D N-terminal like (pseudogene)
UBE2E4P	-3.2	-1.31	0.36	5.55E-05	0.0004	ubiquitin-conjugating enzyme E2E 4 pseudogene
UBE2G2	3.37	0.32	-1.43	5.51E-05	0.0004	ubiquitin conjugating enzyme E2G 2
UBE2H	3.75	0.47	-1.44	2.93E-06	0.0002	ubiquitin conjugating enzyme E2H
UBE2W	-3.16	-1.36	0.3	9.34E-05	0.0005	ubiquitin-conjugating enzyme E2W (putative)
UBE2Z	-3.16	-1.38	0.28	5.64E-05	0.0004	ubiquitin-conjugating enzyme E2Z
UBIAD1	-3.04	-1.28	0.33	5.61E-05	0.0004	UbiA prenyltransferase domain containing 1
UBL3	-3.43	-1.41	0.36	0.0001	0.0006	ubiquitin-like 3
UBL7	-3.13	-1.36	0.28	5.52E-05	0.0004	ubiquitin-like 7
UCN3	3.41	0.32	-1.44	9.86E-06	0.0002	urocortin 3
UFSP1	-3.29	-1.31	0.4	1.48E-05	0.0002	UFM1-specific peptidase 1 (inactive)
UHRF1	3.13	0.44	-1.2	5.84E-05	0.0004	ubiquitin-like with PHD and ring finger domains 1

ULK1	3.34	0.33	-1.41	2.65E-05	0.0003	unc-51 like autophagy activating kinase 1
UMODL1	3.12	0.26	-1.38	5.04E-06	0.0002	uromodulin-like 1
UNC45A	-3.21	-1.33	0.35	5.35E-06	0.0002	unc-45 myosin chaperone A
UNC80	3.1	0.25	-1.38	5.87E-05	0.0004	unc-80 homolog (C. elegans)
UNK	-3.06	-1.28	0.34	7.00E-05	0.0004	unkempt family zinc finger
UPK2	-3.01	-1.31	0.28	4.44E-06	0.0002	uroplakin 2
UPP2	-3.06	-1.36	0.25	4.20E-06	0.0002	uridine phosphorylase 2
UQCC2	-3.14	-1.35	0.3	6.97E-06	0.0002	ubiquinol-cytochrome c reductase complex assembly factor 2
URAD	-3.23	-1.41	0.28	0.0001	0.0006	ureidoimidazoline (2-oxo-4-hydroxy-4-carboxy-5-) decarboxylase
UROD	-3.1	-1.33	0.3	4.63E-05	0.0003	uroporphyrinogen decarboxylase
USMG5; MIR1307	-3.17	-1.33	0.33	2.36E-05	0.0002	up-regulated during skeletal muscle growth 5 homolog (mouse); microRNA 1307
USP21	3.3	0.32	-1.4	3.89E-06	0.0002	ubiquitin specific peptidase 21
USP22	3.01	0.3	-1.29	8.23E-06	0.0002	ubiquitin specific peptidase 22
USP27X	3.35	0.32	-1.42	1.15E-05	0.0002	ubiquitin specific peptidase 27, X-linked
UST	-3.01	-1.31	0.28	6.59E-06	0.0002	uronyl-2-sulfotransferase
UTS2R	-3.24	-1.31	0.39	1.54E-05	0.0002	urotensin 2 receptor
VANGL1	-3.05	-1.34	0.27	3.99E-06	0.0002	VANGL planar cell polarity protein 1
VANGL2	3.42	0.39	-1.38	4.42E-05	0.0003	VANGL planar cell polarity protein 2
VASN	-3.04	-1.28	0.32	2.88E-05	0.0003	vasorin
VAV1	3.06	0.41	-1.2	2.57E-05	0.0003	vav 1 guanine nucleotide exchange factor
VCPIP1	-3.09	-1.35	0.28	5.80E-05	0.0004	valosin containing protein (p97)/p47 complex interacting protein 1
VEGFA	3.57	0.43	-1.4	3.30E-05	0.0003	vascular endothelial growth factor A
VGLL3	-3.09	-1.35	0.28	9.49E-05	0.0005	vestigial-like family member 3
VNN1	3.21	0.39	-1.29	1.01E-05	0.0002	vanin 1
VPS51	3.05	0.32	-1.29	1.74E-05	0.0002	vacuolar protein sorting 51 homolog (S. cerevisiae)
VPS53	-3.13	-1.38	0.26	9.47E-06	0.0002	vacuolar protein sorting 53 homolog (S. cerevisiae)
VRK3	3.19	0.47	-1.2	7.24E-06	0.0002	vaccinia related kinase 3
VTRNA1-1	-3.01	-1.36	0.23	3.95E-05	0.0003	vault RNA 1-1
VWF	-3.01	-1.31	0.28	1.98E-05	0.0002	von Willebrand factor
WBP2NL	3.26	0.32	-1.38	7.27E-06	0.0002	WBP2 N-terminal like
WDPCP	3.37	0.32	-1.43	3.11E-06	0.0002	WD repeat containing planar cell polarity effector
WDR13	-3.11	-1.31	0.32	1.23E-05	0.0002	WD repeat domain 13
WDR36	3.3	0.32	-1.4	3.06E-05	0.0003	WD repeat domain 36
WDR45	-3.07	-1.32	0.29	3.88E-05	0.0003	WD repeat domain 45
WDR86	3.02	0.39	-1.2	6.54E-06	0.0002	WD repeat domain 86
WDR86	3.19	0.29	-1.38	9.78E-05	0.0005	WD repeat domain 86
WDR91	-3.11	-1.31	0.32	1.15E-05	0.0002	WD repeat domain 91
WDR97	-3.07	-1.3	0.32	5.58E-06	0.0002	WD repeat domain 97

WFIKKN1	3.42	0.39	-1.38	4.40E-06	0.0002	WAP, follistatin/kazal, immunoglobulin, kunitz and netrin domain containing 1
WHAMMP3	-3.22	-1.36	0.33	3.52E-05	0.0003	WAS protein homolog associated with actin, golgi membranes and microtubules pseudogene 3
WIPI1	-3.08	-1.37	0.25	3.15E-05	0.0003	WD repeat domain, phosphoinositide interacting 1
WNK2	3.06	0.32	-1.29	5.59E-06	0.0002	WNK lysine deficient protein kinase 2
WNK4	-3.24	-1.37	0.32	2.29E-05	0.0002	WNK lysine deficient protein kinase 4
WNT16	-3.06	-1.28	0.33	6.40E-05	0.0004	wingless-type MMTV integration site family, member 16
WT1	-3.15	-1.33	0.32	8.10E-06	0.0002	Wilms tumor 1
WWOX	3.11	0.34	-1.29	1.23E-05	0.0002	WW domain containing oxidoreductase
XIRP1	-3.05	-1.34	0.27	0.0004	0.0016	xin actin binding repeat containing 1
XPA	-3.15	-1.31	0.34	4.50E-05	0.0003	xeroderma pigmentosum, complementation group A
XPO4	-3.17	-1.37	0.29	2.53E-05	0.0003	exportin 4
XPO7	-3.27	-1.43	0.28	0.0002	0.0007	exportin 7
XRRA1	-3.09	-1.35	0.28	0.0001	0.0007	X-ray radiation resistance associated 1
YBX1	-3.18	-1.28	0.39	5.45E-05	0.0004	Y box binding protein 1
YIPF1	-3.16	-1.38	0.28	3.83E-05	0.0003	Yip1 domain family member 1
YWHAG	-3.1	-1.37	0.26	3.20E-05	0.0003	tyrosine 3-monooxygenase/tryptophan 5-monooxygenase activation protein, gamma
ZAP70	3.26	0.32	-1.38	4.40E-06	0.0002	zeta chain of T cell receptor associated protein kinase 70kDa
ZAR1	3.15	0.27	-1.38	3.63E-06	0.0002	zygote arrest 1
ZBED6; ZC3H11A	-3.31	-1.36	0.36	7.58E-06	0.0002	zinc finger, BED-type containing 6; zinc finger CCCH-type containing 11A
ZBTB10	3.15	0.45	-1.2	1.11E-05	0.0002	zinc finger and BTB domain containing 10
ZBTB34	3.3	0.32	-1.4	8.63E-06	0.0002	zinc finger and BTB domain containing 34
ZBTB6	-3.17	-1.36	0.3	6.02E-06	0.0002	zinc finger and BTB domain containing 6
ZBTB7C	3.73	0.5	-1.4	4.54E-06	0.0002	zinc finger and BTB domain containing 7C
ZBTB9; SYNGAP1	-3.13	-1.28	0.36	5.03E-06	0.0002	zinc finger and BTB domain containing 9; synaptic Ras GTPase activating protein 1
ZCCHC12	-3.29	-1.32	0.4	5.79E-06	0.0002	zinc finger, CCHC domain containing 12
ZCCHC16	-3.01	-1.31	0.28	2.80E-05	0.0003	zinc finger, CCHC domain containing 16
ZCCHC24	-3.03	-1.32	0.27	1.52E-05	0.0002	zinc finger, CCHC domain containing 24
ZCCHC3	3.29	0.32	-1.4	1.57E-05	0.0002	zinc finger, CCHC domain containing 3
ZDHHC1	-3.05	-1.36	0.25	7.73E-05	0.0005	zinc finger, DHHC-type containing 1
ZDHHC14	-3.17	-1.31	0.35	1.15E-05	0.0002	zinc finger, DHHC-type containing 14
ZER1	3.23	0.29	-1.4	3.51E-06	0.0002	zyg-11 related, cell cycle regulator
ZFP90	-3.11	-1.36	0.28	1.14E-05	0.0002	ZFP90 zinc finger protein
ZG16B	-3.18	-1.35	0.32	6.29E-05	0.0004	zymogen granule protein 16B
ZIK1	3.45	0.4	-1.38	2.59E-05	0.0003	zinc finger protein interacting with K protein 1
ZKSCAN1	-3.04	-1.28	0.32	9.53E-05	0.0005	zinc finger with KRAB and SCAN domains 1
ZKSCAN7	-3.3	-1.38	0.34	0.0001	0.0006	zinc finger with KRAB and SCAN domains 7
ZMAT1	3.42	0.39	-1.38	9.09E-05	0.0005	zinc finger, matrin-type 1
ZMYND12	-3.04	-1.37	0.23	1.06E-05	0.0002	zinc finger, MYND-type containing 12

ZNF157	-3.01	-1.31	0.28	1.19E-05	0.0002	zinc finger protein 157
ZNF174	-3.15	-1.44	0.21	3.56E-05	0.0003	zinc finger protein 174
ZNF175	-3.07	-1.34	0.28	0.0002	0.0010	zinc finger protein 175
ZNF182; ZNF630	-3.04	-1.31	0.29	4.27E-06	0.0002	zinc finger protein 182; zinc finger protein 630
ZNF212	-3.01	-1.31	0.28	0.0001	0.0007	zinc finger protein 212
ZNF217	3.52	0.43	-1.38	2.72E-06	0.0002	zinc finger protein 217
ZNF227	3.46	0.32	-1.47	2.27E-05	0.0002	zinc finger protein 227
ZNF23	-3.2	-1.31	0.36	8.45E-06	0.0002	zinc finger protein 23
ZNF250; COMMD5	-3.11	-1.31	0.32	2.22E-05	0.0002	zinc finger protein 250; COMM domain containing 5
ZNF256	3.56	0.38	-1.46	1.21E-05	0.0002	zinc finger protein 256
ZNF29P	3.67	0.43	-1.44	1.08E-05	0.0002	zinc finger protein 29, pseudogene
ZNF3	3.25	0.31	-1.39	9.25E-06	0.0002	zinc finger protein 3
ZNF317	3.02	0.39	-1.2	0.0003	0.0014	zinc finger protein 317
ZNF322	-3.06	-1.34	0.27	1.98E-05	0.0002	zinc finger protein 322
ZNF34	-3.14	-1.35	0.3	7.73E-06	0.0002	zinc finger protein 34
ZNF341	-3.01	-1.31	0.28	5.61E-05	0.0004	zinc finger protein 341
ZNF345	3.3	0.32	-1.4	1.01E-05	0.0002	zinc finger protein 345
ZNF37BP	-3.13	-1.4	0.25	0.0001	0.0006	zinc finger protein 37B, pseudogene
ZNF408	-3.11	-1.31	0.32	0.0001	0.0005	zinc finger protein 408
ZNF410	-3.15	-1.38	0.27	9.84E-06	0.0002	zinc finger protein 410
ZNF417	-3.22	-1.39	0.3	0.0002	0.0009	zinc finger protein 417
ZNF439	-3.43	-1.41	0.36	4.25E-05	0.0003	zinc finger protein 439
ZNF460	-3.18	-1.41	0.26	5.67E-05	0.0004	zinc finger protein 460
ZNF516	-3.26	-1.38	0.32	2.58E-05	0.0003	zinc finger protein 516
ZNF534	-3.15	-1.3	0.36	3.67E-06	0.0002	zinc finger protein 534
ZNF543	3.35	0.32	-1.42	7.07E-06	0.0002	zinc finger protein 543
ZNF551	-3.04	-1.28	0.32	5.12E-05	0.0004	zinc finger protein 551
ZNF563	-3.26	-1.38	0.32	8.51E-06	0.0002	zinc finger protein 563
ZNF564	3.03	0.4	-1.2	6.43E-06	0.0002	zinc finger protein 564
ZNF566	-3.16	-1.38	0.28	0.0001	0.0006	zinc finger protein 566
580; ZNF581; CCD(	-3.05	-1.36	0.24	3.41E-05	0.0003	zinc finger protein 580; zinc finger protein 581; coiled-coil domain containing 106
ZNF582	-3.31	-1.38	0.34	6.15E-06	0.0002	zinc finger protein 582
ZNF586	-3.19	-1.37	0.3	1.56E-05	0.0002	zinc finger protein 586
ZNF587; UBE2CP5	3.15	0.23	-1.43	1.11E-05	0.0002	zinc finger protein 587; ubiquitin conjugating enzyme E2C pseudogene 5
ZNF592	3.1	0.25	-1.38	3.77E-05	0.0003	zinc finger protein 592
ZNF624	-3.45	-1.42	0.36	5.47E-05	0.0004	zinc finger protein 624
IF625-ZNF20; ZNF6	3.02	0.17	-1.43	4.38E-06	0.0002	ZNF625-ZNF20 readthrough (NMD candidate); zinc finger protein 625
ZNF668	3.34	0.36	-1.38	1.06E-05	0.0002	zinc finger protein 668

ZNF671	3.19	0.47	-1.2	2.13E-05	0.0002	zinc finger protein 671
ZNF697	-3.26	-1.38	0.32	3.78E-06	0.0002	zinc finger protein 697
ZNF71	3.67	0.43	-1.44	9.62E-06	0.0002	zinc finger protein 71
ZNF716	3.84	0.52	-1.43	1.34E-05	0.0002	zinc finger protein 716
ZNF720	3.19	0.47	-1.2	1.66E-05	0.0002	zinc finger protein 720
ZNF721; ABCA11P	-3.16	-1.38	0.28	8.64E-05	0.0005	zinc finger protein 721; ATP binding cassette subfamily A member 11, pseudogene
ZNF750	3.2	0.28	-1.4	7.83E-06	0.0002	zinc finger protein 750
ZNF764	3.03	0.4	-1.2	1.19E-05	0.0002	zinc finger protein 764
ZNF770	-3.19	-1.4	0.28	5.12E-06	0.0002	zinc finger protein 770
ZNF774	-3.03	-1.41	0.19	0.0001	0.0006	zinc finger protein 774
ZNF787	-3.22	-1.28	0.4	9.60E-06	0.0002	zinc finger protein 787
ZNF793	-3.22	-1.36	0.32	5.16E-05	0.0004	zinc finger protein 793
ZNF799	3.26	0.32	-1.38	3.18E-05	0.0003	zinc finger protein 799
ZNF80	3.32	0.35	-1.38	5.76E-05	0.0004	zinc finger protein 80
ZNF804B	-3.05	-1.28	0.33	1.50E-05	0.0002	zinc finger protein 804B
ZNF85	-3.12	-1.36	0.28	4.27E-05	0.0003	zinc finger protein 85
ZNF852	3.44	0.38	-1.4	1.57E-05	0.0002	zinc finger protein 852
ZNF862	-3.04	-1.28	0.32	0.0002	0.0008	zinc finger protein 862
ZNF90	3.33	0.34	-1.4	1.94E-05	0.0002	zinc finger protein 90
ZNHIT6	-3.2	-1.42	0.26	3.39E-05	0.0003	zinc finger, HIT-type containing 6
ZSCAN5B	3.34	0.36	-1.38	3.00E-06	0.0002	zinc finger and SCAN domain containing 5B
ZSWIM4	-3.02	-1.36	0.23	1.34E-05	0.0002	zinc finger, SWIM-type containing 4
ZW10	3.26	0.32	-1.38	2.68E-06	0.0002	zw10 kinetochore protein
ZWINT	-3.04	-1.28	0.32	2.03E-05	0.0002	ZW10 interacting kinetochore protein

Table. S2. Enrichment results of top ten Process Networks regulated by colostrum exosome capsulated oligosaccharides.

	Process Networks	Total	pValue	Min FDR	p-value	FDR	In Data	Network Objects from Active Data
1	Inflammation_TREM1 signaling	145	6.714E-05	9.266E-03	6.714E-05	9.266E-03	9	IRAK2, IRAK1/2, tBid, I-kB, MEK1/2, ITGB1, MEK2(MAP2K2), jBid, Bid
2	Inflammation_NK cell cytotoxicity	164	1.731E-04	1.195E-02	1.731E-04	1.195E-02	9	FADD, NFKBIE, Perforin, I-kB, MEK1/2, KIR3DL1, MEK2(MAP2K2), KIR3DL3, Bid
3	Inflammation_IL-4 signaling	115	5.082E-04	2.338E-02	5.082E-04	2.338E-02	7	tBid, p70 S6 kinase1, I-kB, MEK1/2, MEK2(MAP2K2), jBid, Bid
4	Inflammation_Neutrophil activation	215	1.253E-03	3.958E-02	1.253E-03	3.958E-02	9	Rac2, FADD, tBid, NFKBIE, I-kB, MEK1/2, MEK2(MAP2K2), Bid, ICAM4
5	Immune response_BCR pathway	137	1.434E-03	3.958E-02	1.434E-03	3.958E-02	7	Kappa chain (Ig light chain), p70 S6 kinase1, NFKBIE, Profilin, I-kB, MEK1/2, MEK2(MAP2K2)
6	Apoptosis_Apoptosis stimulation by external signals	144	1.911E-03	4.395E-02	1.911E-03	4.395E-02	7	FADD, tBid, TWEAK(TNFSF12), I-kB, MEK2(MAP2K2), jBid, Bid
7	Inflammation_Amphoterin signaling	118	3.236E-03	5.821E-02	3.236E-03	5.821E-02	6	IRAK2, IRAK1/2, NFKBIE, I-kB, MEK1/2, MEK2(MAP2K2)
8	Inflammation_IL-6 signaling	119	3.374E-03	5.821E-02	3.374E-03	5.821E-02	6	NFKBIE, AGP1 (ORM1), I-kB, MEK1/2, HDL proteins, MEK2(MAP2K2)
9	Cell adhesion_Integrin-mediated cell-matrix adhesion	214	4.703E-03	7.212E-02	4.703E-03	7.212E-02	8	Profilin I, RASGRF1, Tubulin alpha, Profilin, MEK1/2, ITGB1, MEK2(MAP2K2), RhoGDI alpha
10	Immune response_Phagocytosis	222	5.853E-03	7.343E-02	5.853E-03	7.343E-02	8	ILT2, Profilin I, APOA2, NFKBIE, Profilin, I-kB, ITGB1, HDL proteins

Table. S3. Enrichment results of top ten Pathway Maps regulated by colostrum exosome capsulated oligosaccharides.

#	Maps	Total	pValue	Min FDR	p-value	FDR	In Data	Network Objects from Active Data
1	Apoptosis and survival_TNFR1 signaling pathway	43	2.386E-05	1.117E-02	2.386E-05	1.117E-02	5	FADD, tBid, I-kB, jBid, Bid
2	IGF family signaling in colorectal cancer	60	1.215E-04	1.924E-02	1.215E-04	1.924E-02	5	COX-2 (PTGS2), p70 S6 kinase1, I-kB, MEK1/2, MAFG
3	Apoptosis and survival_Granzyme B signaling	32	1.233E-04	1.924E-02	1.233E-04	1.924E-02	4	tBid, Tubulin alpha, Perforin, Bid
4	Cytoskeleton remodeling_Substance P mediated membrane blebbing	16	2.698E-04	2.815E-02	2.698E-04	2.815E-02	3	Tubulin alpha, Dynamin-1, Dynamin
5	Apoptosis and survival_Apoptotic TNF-family pathways	42	3.609E-04	2.815E-02	3.609E-04	2.815E-02	4	FADD, tBid, TWEAK(TNFSF12), Bid
6	Apoptosis and survival_Anti-apoptotic TNFs/NF-kB/Bcl-2 pathway	42	3.609E-04	2.815E-02	3.609E-04	2.815E-02	4	IRAK1/2, Sequestosome 1(p62), TWEAK(TNFSF12), I-kB
7	Expression targets of Tissue factor signaling in cancer	22	7.163E-04	4.789E-02	7.163E-04	4.789E-02	3	NFKBIE, ITGB1, CTGF
8	Immune response_TLR2 and TLR4 signaling pathways	57	1.161E-03	6.793E-02	1.161E-03	6.793E-02	4	IRAK2, COX-2 (PTGS2), I-kB, MEK1/2
9	Glucocorticoid-induced elevation of intraocular pressure as glaucoma risk f	62	1.590E-03	8.269E-02	1.590E-03	8.269E-02	4	COX-2 (PTGS2), SENP1, ITGB1, RhoGDI alpha
10	Apoptosis and survival_Role of IAP-proteins in apoptosis	31	1.983E-03	9.283E-02	1.983E-03	9.283E-02	3	FADD, tBid, Bid
